# On-surface synthesis and interfacial charge redistribution of open-shell [3]triangulene-fused porphyrins on Au(111)

**DOI:** 10.1093/nsr/nwag155

**Published:** 2026-03-11

**Authors:** Miguel Martínez García, Feifei Xiang, Andres Ortega-Guerrero, Manish Kumar, Nicolò Bassi, Carlo A Pignedoli, Pascal Ruffieux, Tomas Torres, Pavel Jelínek, Roman Fasel, Giovanni Bottari

**Affiliations:** Departamento de Química Orgánica, Universidad Autónoma de Madrid, Campus de Cantoblanco, Madrid 28049, Spain; IMDEA Nanociencia, Campus de Cantoblanco, Madrid 28049, Spain; nanotech@surfaces Laboratory, Empa-Swiss Federal Laboratories for Materials Science and Technology, Dübendorf 8600, Switzerland; School of Advanced Manufacturing Engineering, Nanjing University, Suzhou 215163, China; nanotech@surfaces Laboratory, Empa-Swiss Federal Laboratories for Materials Science and Technology, Dübendorf 8600, Switzerland; Institute of Physics, Czech Academy of Sciences, Prague 16200, Czech Republic; nanotech@surfaces Laboratory, Empa-Swiss Federal Laboratories for Materials Science and Technology, Dübendorf 8600, Switzerland; nanotech@surfaces Laboratory, Empa-Swiss Federal Laboratories for Materials Science and Technology, Dübendorf 8600, Switzerland; nanotech@surfaces Laboratory, Empa-Swiss Federal Laboratories for Materials Science and Technology, Dübendorf 8600, Switzerland; Departamento de Química Orgánica, Universidad Autónoma de Madrid, Campus de Cantoblanco, Madrid 28049, Spain; IMDEA Nanociencia, Campus de Cantoblanco, Madrid 28049, Spain; Institute for Advanced Research in Chemical Sciences (IAdChem), Universidad Autónoma de Madrid, Madrid 28049, Spain; Institute of Physics, Czech Academy of Sciences, Prague 16200, Czech Republic; nanotech@surfaces Laboratory, Empa-Swiss Federal Laboratories for Materials Science and Technology, Dübendorf 8600, Switzerland; Department of Chemistry, Biochemistry and Pharmaceutical Sciences, University of Bern, Bern 3012, Switzerland; Departamento de Química Orgánica, Universidad Autónoma de Madrid, Campus de Cantoblanco, Madrid 28049, Spain; IMDEA Nanociencia, Campus de Cantoblanco, Madrid 28049, Spain; Institute for Advanced Research in Chemical Sciences (IAdChem), Universidad Autónoma de Madrid, Madrid 28049, Spain

**Keywords:** graphene, magnetism, many-body states, scanning tunneling microscopy, charge transfer

## Abstract

The on-surface synthesis of porphyrin-nanographene (Por-NG) hybrids enables systematic control of *π*-electron magnetism in organic materials, yet the spin behavior of these systems remains difficult to predict because the porphyrin core perturbs the graphene lattice. We report the fabrication and electronic characterization of ZnPors fused with two and four [3]triangulene units (i.e. **ZnPorT_2_** and **ZnPorT_4_**, respectively) on Au(111). Rapid thermal annealing maximizes the yield of discrete hybrids by suppressing surface diffusion and unwanted lateral fusion. Scanning tunneling microscopy and spectroscopy, supported by theory, show that both **ZnPorT_2_** and **ZnPorT_4_** exhibit diradical character with an antiferromagnetically coupled ground state. The hybrids undergo interfacial charge transfer to the metallic substrate: **ZnPorT_2_** donates one electron, forming open-shell **ZnPorT_2_^•^^+^**, while **ZnPorT_4_** donates two electrons, affording closed-shell **ZnPorT_4_^2+^**. Despite this charge transfer, the multireference character of the frontier orbitals remains in **ZnPorT_2_^•^^+^**. The results establish an efficient route to complex Por-NG hybrids and clarify how molecular design and interfacial charge transfer shape their magnetic properties, an essential step toward functional magnetic nanoarchitectures.

## Teaser

On-surface synthesis of ZnPor–triangulene hybrids enables controllable π-magnetism. Scanning tunnelling microscopy and theoretical calculations reveal an antiferromagnetic coupled ground state and charge-transfer–tuned multireference states, advancing the design of magnetic carbon-based nano-architectures.

## INTRODUCTION

The emergence of *π*-magnetism in all-carbon nanographenes (NGs), propelled by their intrinsically weak spin–orbit coupling and long spin-coherence times, has established these systems at the forefront of molecular spintronics and quantum materials research. In such open-shell nanostructures, the magnetic character arises from the presence of unpaired *π*-electrons, which can be engineered through sublattice imbalance [1–3], topological frustration [4,5], Coulomb repulsion [6], incorporation of non-hexagonal rings [7] and/or the presence of heteroatoms [8].

Over the last decade, on-surface chemistry involving appropriate, solution-synthesized NG precursors has enabled the fabrication of a myriad of surface-supported, open-shell NGs featuring multiple spin states [9–11] and tunable exchange interactions [1,4,12,13], fostering fundamental studies of spin phenomena.

Among the diverse strategies for creating novel open-shell NGs, fusing them with porphyrins (Pors) emerges as a particularly promising approach [14,15]. Pors are highly appealing platforms for this purpose due to their large aromatic 18 *π*-electron conjugated system, and structural versatility [16]. Furthermore, their ability to chelate transition metals within the central cavity potentially enables *π–*d electron interactions in the resulting Por-NG hybrids [17–19]. A logical route to fabricate such surface-supported hybrids involves preparing, through solution chemistry, Pors functionalized at their *meso* positions with molecular fragments that, upon on-surface activation, yield open-shell NG moieties fused to the Por macrocycle. However, a significant challenge arises when addressing the magnetic features of such hybrids. While the magnetic ground states of pure NGs can often be predicted using Lieb’s theorem [20], and the empirical Clar’s sextet rule [21] can also provide useful information on the most contributed resonance structures, these models become inadequate for Por-NG hybrids. The Por core, with its four pyrrolic rings and annulene-like conjugation, disrupts the ideal bipartite lattice, making the spin configuration of the resulting hybrid challenging to predict. Previously, Pors fused with two or four phenalenyl units (i.e. the smallest non-Kekulé polyaromatic hydrocarbon) were prepared [22,23]. The hybrid with two phenalenyl units exhibited a ferromagnetic ground state, with the two spins primarily localized on the Por backbone. In contrast, the Por-NG system featuring four phenalenyl fragments underwent interfacial charge transfer with the substrate, resulting in an *S* = 1/2 spin state. Given the wide variation among the reported Por-NG systems, there is a clear need to develop more adequate guidelines and theoretical frameworks to better understand the magnetic ground states of such nanostructures.

To advance the field of open-shell *π*-extended Pors, we report here the on-surface synthesis and electronic characterization of **ZnPorT_2_** and **ZnPorT_4_**, two Pors fused with two and four [3]triangulenes, zigzag-edged triangular graphene fragments that possess a triplet ground state arising from their two delocalized unpaired electrons [1]. Using a combination of scanning tunneling microscopy (STM)/spectroscopy (STS) and theoretical calculations, we demonstrate that both Por-NG hybrids feature a diradical character with an antiferromagnetically coupled ground state. Interfacial charge transfer with the Au(111) substrate quenches one spin in **ZnPorT_2_** and two spins in **ZnPorT_4_**. Despite the charge transfer perturbation, **ZnPorT_2_** exhibits strong multireference characters in its frontier orbitals, as is evidenced by many-body calculations. This work not only enriches the family of Por-NG hybrids but also provides a versatile platform for understanding and controlling spin interactions in complex, multiradical systems.

## RESULTS AND DISCUSSION

### Solution synthesis of ZnPorT_2_ and ZnPorT_4_ precursors

**Scheme 1.**
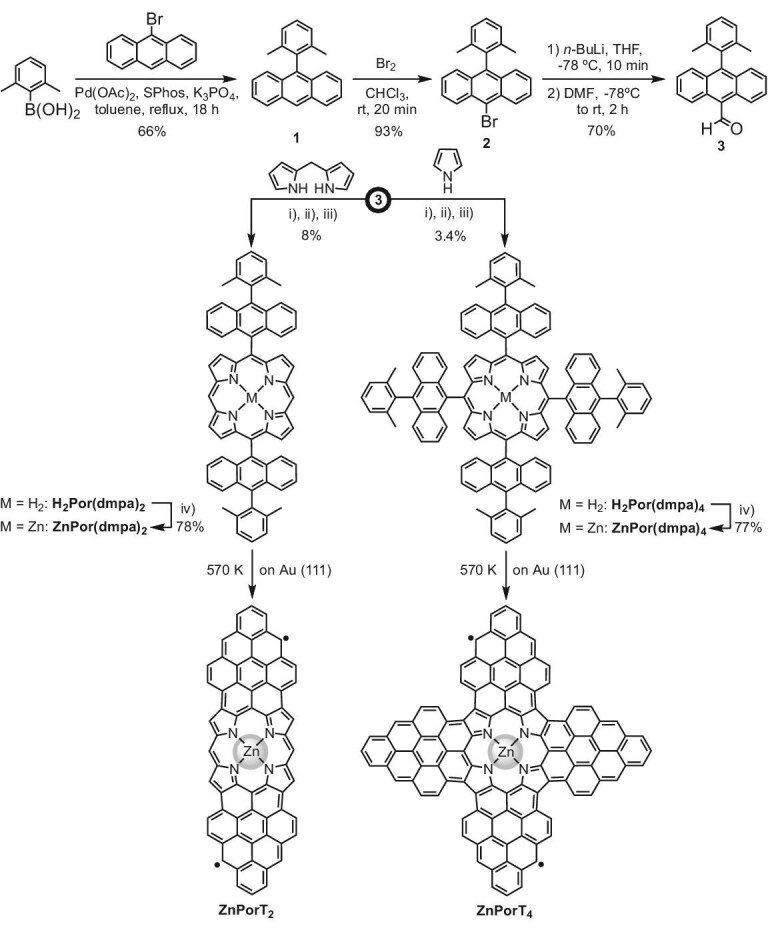
Synthetic routes towards **ZnPorT_2_** and **ZnPorT_4_**. Reagents and conditions: (i) BF_3_(OEt_2_), CHCl_3_, RT; (ii) DDQ, reflux, (iii) NEt_3_, RT; (iv) Zn(OAc)_2_, THF, reflux. DDQ = 2,3-dichloro-5,6-dicyano-1,4-benzoquinone.

The synthetic route to prepare **ZnPorT_2_** and **ZnPorT_4_** started with a Pd-catalyzed Suzuki–Miyaura cross-coupling reaction between commercially available 9-bromoanthracene and 2,6-dimethylphenylboronic acid, affording derivative **1** (Scheme 1). The selective bromination of **1** at position 10 of its anthryl moiety yielded compound **2**, which was transformed into aldehyde **3** by a formylation reaction. Compound **3** was then used as a common reactant for the synthesis of both *D*_2h_-symmetric **H_2_Por(dmpa)_2_**and *D*_4h_-symmetric **H_2_Por(dmpa)_4_**, precursors of **ZnPorT_2_** and **ZnPorT_4_**, respectively, which were obtained by condensation of **3** with *meso*-*H*-dipyrromethane [in the case of **H_2_Por(dmpa)_2_**] and pyrrole [in the case of **H_2_Por(dmpa)_4_**] (see Section S2 of the Supplementary data for more details). Subsequent metalation of **H_2_Por(dmpa)_2_** and **H_2_Por(dmpa)_4_** with Zn(OAc)_2_ in refluxing tetrahydrofuran afforded the corresponding zinc metalated derivatives **ZnPor(dmpa)_2_** and **ZnPor(dmpa)_4_**, respectively. **ZnPor(dmpa)_2_** and **ZnPor(dmpa)_4_**, as well as their precursors, were fully characterized by several spectroscopic and spectrometric techniques (see Section S2).

### On-surface synthesis of ZnPorT_2_ and ZnPorT_4_

**Figure 1.**
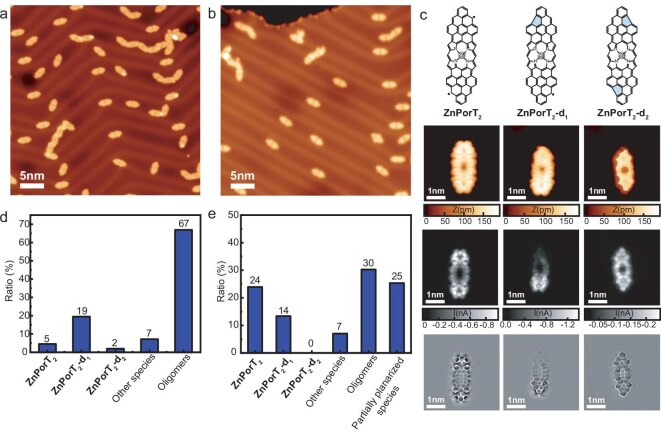
Large-scale scanning tunneling microscopy (STM) images obtained at 4.5 K after depositing **ZnPor(dmpa)_2_** on a clean Au(111) substrate kept at (a) 570 K (i.e. hot deposition protocol) and (b) room temperature (RT), followed by annealing to 570 K with an average heating rate of 108 K/min and keeping the sample at the final temperature for 3 min (i.e. flash annealing protocol). (c) Chemical structure of **ZnPorT_2_, ZnPorT_2_-d_1_** and **ZnPorT_2_-d_2_** (top row) and their corresponding high-resolution STM images (second row), bond-resolved STM (BR-STM) images acquired by a CO-functionalized tip in constant height (CH) mode (third row), and their Laplace-filtered images (bottom row). Statistical analysis of the different species obtained from (d) the hot deposition and (e) the flash annealed samples. In each sample, more than 130 molecules from different regions were considered. Scanning parameters: (a) *U* = −1.5 V, *I* = 40 pA; (b) *U* = − 0.01 V, *I* = 100 pA; (c) STM: *U* = −0.01 V, *I* = 100 pA; BR-STM: *U* = −5 mV, *I* = 200 pA before switching off the feedback. In (c) (top row), the five-member rings in **ZnPorT_2_-d_1_** and **ZnPorT_2_-d_2_** have been filled in light blue for easier identification.

To obtain **ZnPorT_2_**, derivative **ZnPor(dmpa)_2_** was deposited on a clean Au(111) surface kept at 570 K (i.e. hot deposition protocol) to promote the surface-assisted intramolecular cyclodehydrogenation reaction (Scheme 1). Large-scale STM imaging reveals the presence of fully planarized, ‘elongated’ units, along with irregular chain-type structures formed by intermolecular coupling (Fig. 1a). A closer analysis of the monomeric species suggests the formation of three main structures, which could be tentatively assigned to *D*_2h_-symmetric **ZnPorT_2_** and ‘defective’ species **ZnPorT_2_-d_1_** and **ZnPorT_2_-d_2_**(Fig. 1c, first and second rows from the top). The formation of the two latter species, featuring one or two pentagon rings on one or both of the Por triangulene moieties, respectively, are due to the loss of a methyl group during the surface-promoted cyclodehydrogenation reaction, a process previously observed during the on-surface synthesis of other nanographene systems [5,24]. Bond-resolved STM (BR-STM) measurements using a CO-functionalized tip in constant height (CH) mode confirmed the chemical structure of the three *π*-extended Pors (Fig. 1c, third and bottom rows from the top).

To minimize the intermolecular covalent coupling and reduce the methyl ‘extrusion’ side-reaction, thus increasing the yield of **ZnPorT_2_**, an alternative preparation protocol was employed. This involved the deposition of **ZnPor(dmpa)_2_** on Au(111) kept at room temperature (RT) followed by a rapid increase of the sample temperature to 570 K (i.e. average heating rate of ∼108 K/min), and keeping the sample at the final temperature for 3 min (i.e. flash annealing protocol). Large-scale STM imaging of the resulting samples suggests a reduction of Por cross-linking reactions with respect to the hot deposition protocol (Fig. 1b). Statistical analysis of the flash-annealed sample shows a relative increase in the formation of target **ZnPorT_2_** with respect to the hot deposition protocol (i.e. 24% vs. 5%), together with a substantial decrease of the relative abundance of oligomeric species (i.e. 30% vs. 67%) (Fig. 1d and e). These findings could be rationalized, taking into account the lower diffusion length of **ZnPorT_2_** and other reactive species as a consequence of the relatively short annealing time, which reduces the cross-linking between reactive, individual molecules, thus favoring the formation of ‘intact’ species [6,25]. Using the flash annealing protocol, the formation of defective species **ZnPorT_2_-d_1_** and **ZnPorT_2_-d_2_** is also reduced but only by 7%, suggesting that the loss of methyl groups has an activation energy close to that of the intramolecular cyclodehydrogenation, and therefore cannot be completely avoided. The loss of methyl groups during the thermal deposition process cannot be ruled out either. The appearance of partially planarized species (i.e. 25%) upon flash annealing can be attributed to the shorter heating treatment, which in turn reduces the ‘yield’ of the intramolecular cyclodehydrogenation reaction.

**Figure 2.**
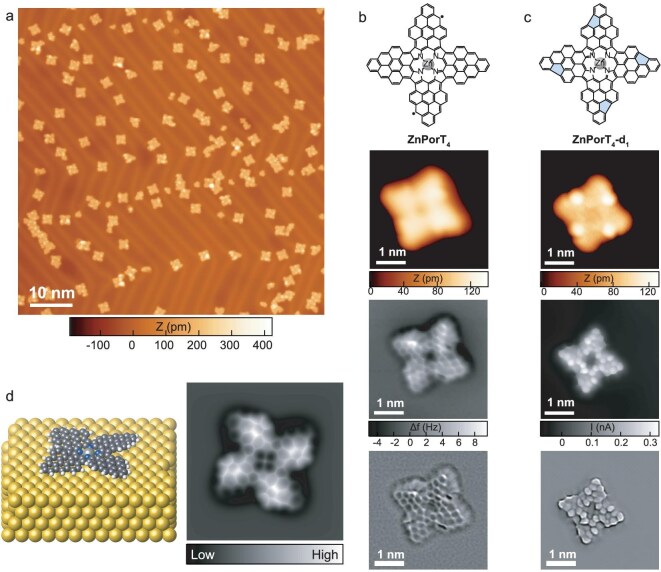
(a) Large-scale STM image obtained from the sample prepared by depositing **ZnPor(dmpa)_4_** on an Au(111) substrate kept at RT, followed by annealing to 570 K with an average heating rate of 108 K/min for 3 min (i.e. flash-annealing protocol). (b) Chemical structure of **ZnPorT_4_** (top row) and its corresponding high-resolution STM (second row), nc-AFM (third row) and Laplace-filtered images (bottom row). (c) Chemical structure of **ZnPorT_4_-d_1_** (top) and its corresponding high-resolution STM (second row) and BR-STM images acquired by a CO-functionalized tip in CH mode (third row), and Laplace-filtered image (bottom row). (d) Geometry-optimized model of **ZnPorT_4_** on Au(111) (left) and the corresponding simulated nc-AFM image (right). Scanning parameters: (a) *U* = −1 V, *I* = 30 pA; (b) STM (second row): *U* = −1 V, *I* = 20 pA; nc-AFM (third row): *U* = −5 mV, *I* = 150 pA before switching off the feedback; (c) STM (second row): *U* = −1 V, *I* = 100 pA; BR-STM (third row): *U* = −5 mV, *I* = 150 pA before switching off the feedback. In (c) (top row), the five-membered rings in **ZnPorT_4_-d_1_** have been filled in light blue for easier identification.

As the flash annealing protocol has proved to be the optimal procedure to maximize the yield of **ZnPorT_2_**, the same synthetic approach was applied to the on-surface synthesis of **ZnPorT_4_**. A large-scale STM image of a flash-annealed Au(111) sample of **ZnPor(dmpa)_4_** predominantly revealed fully planarized, cruciform-shaped nanostructures (Fig. 2a), among which **ZnPorT_4_** could be tentatively identified (Fig. 2b, top and second rows). Definitive structural information of **ZnPorT_4_** was obtained from non-contact atomic force microscopy (nc-AFM) measurements, which revealed that only one side of each triangulene ‘arm’ is clearly imaged, suggesting a non-planar configuration of the *π*-extended Por (Fig. 2b, third and bottom rows). Density functional theory (DFT) calculations for **ZnPorT_4_**in the gas phase and on Au(111) show that **ZnPorT_4_** adopts a *D*_4_-symmetric propeller-like geometry to minimize the steric hindrance between the four triangulene moieties [Fig. 2d (left), Fig. S4.12]. The simulated nc-AFM image based on the geometry-optimized **ZnPorT_4_** configuration adsorbed on Au(111) (Fig. 2d, right) well reproduces the experimental observations (Fig. 2b, third row from the top), confirming the non-planar conformation of **ZnPorT_4_**.

As in the case of the bottom-up fabrication of **ZnPorT_2_**, some ‘defective’ nanostructures resulting from the loss of one or more methyl groups during the cyclodehydrogenation reaction were also observed during the on-surface synthesis of **ZnPorT_4_** (Fig. 2c, Fig. S4.13). Among such defective species, it was possible to identify **ZnPorT_4_-d_1_**, a Por in which each of the four *π*-extended lobes features one pentagon ring, with all four pentagon rings located at the same ‘side’ of the truncated triangulene (i.e. arranged ‘clockwise’ or ‘counterclockwise’ with respect to the Por), as identified by BR-STM (Fig. 2c, third row from the top). In **ZnPorT_4_-d_1_**, the former triangulene *π*-extensions are tilted such that the four pentagon rings point towards the surface (see the four brighter spots in the STM image in Fig. 2c) and the zigzag edges are pointing away from the surface, exhibiting a similar propeller-like conformation as in **ZnPorT_4_** (Fig. S4.18).

### Electronic structure of ZnPorT_2_ and ZnPorT_4_

**Figure 3.**
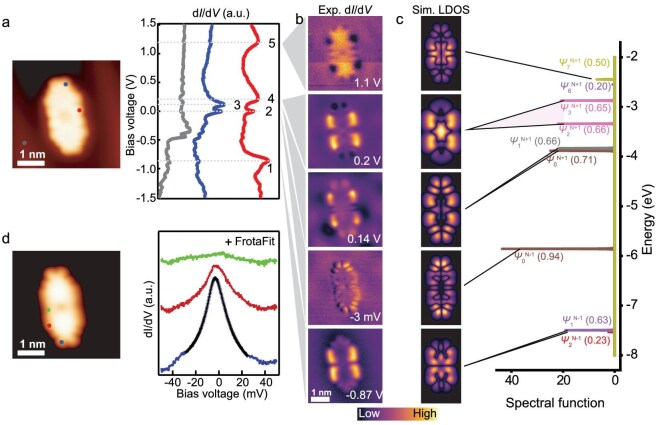
(a) STM image of **ZnPorT_2_** (left) and corresponding d*I*/d*V* spectra taken at specific positions of the STM image (right) (see color code). Lock-in amplitude: 20 mV. (b) d*I*/d*V* maps taken at resonances 1–5 from (a) in constant current (CC) mode. The d*I*/d*V* map acquired at −3 mV was measured with a lock-in amplitude of 1 mV, whereas all other d*I*/d*V* maps were recorded with a lock-in amplitude of 20 mV. (c) Calculated many-body spectral function and corresponding simulated d*I*/d*V* maps of Dyson orbitals shown in Fig. S5.8 (electron removal) and Fig. S5.9 (electron injection) for positively charged **ZnPorT_2_^•^^+^**. (d) (Left) STM image of **ZnPorT_2_** and (right) d*I*/d*V* spectra at specific positions of the STM image on the left recorded in the range of −0.05 to 0.05 V. Lock-in amplitude: 1 mV. Scanning parameters: (a) *U* = −1.5 V, *I* = 500 pA; (b) bias voltage is shown above each map, *I* = 500 pA. (d) *U* = −0.08 V, *I* = 100 pA.

To elucidate the electronic structure of *π*-extended **ZnPorT_2_** and **ZnPorT_4_**, we performed differential conductance (d*I*/d*V*) spectroscopy and mapping, combined with DFT calculations at the Perdew–Burke–Ernzerhof (PBE) and PBE0 level, and complemented by many-body calculations using complete active space self-consistent field (CASSCF) and complete active space configuration interaction (CASCI). In the case of **ZnPorT_2_**, two ionic resonances can be identified via d*I*/d*V* spectroscopy at its triangulene extensions, labeled as 2 and 3 in Fig. 3; one is almost located at zero energy, and the other one is detected at 0.14 V (Fig. 3a and b). The Por core exhibits dominant features at three distinct resonances, labeled as 1, 4 and 5 in Fig. 3a, located at −0.87, 0.2 and 1.1 V, respectively.

DFT calculations predicted that **ZnPorT_2_** has an antiferromagnetic ground state with a singlet-to-triplet energy (Δ*E*_ST_ = *E*_T_ − *E*_S_) gap of 90 meV (Table S5.1). Restricted Kohn–Sham (RKS) DFT calculations at the PBE0 level (Fig. S4.3) show a state at zero energy designated as the highest occupied molecular orbital (HOMO, Ψ_2_ in Fig. S4.3), along with two nearly energetically degenerated lowest unoccupied molecular orbital (LUMO) energy levels slightly above zero energy (Ψ_3_ and Ψ_4_ in Fig. S4.3). Ψ_2_ and Ψ_4_ are predominantly localized at the termini of **ZnPorT_2_** displaying characteristic edge states at its triangulene extensions, whereas Ψ_3_ is primarily contributed from the Por core. To allow for spin polarization due to electron–electron interactions, unrestricted Kohn–Sham (UKS) DFT calculations were performed (Fig. S4.4). The frontier energy states labeled as *Ψ_U2_* and *Ψ_U4_* are found to be spin-polarized, and characterized by a pair of singly occupied/unoccupied molecular orbitals (SOMOs/SUMOs), in which the dominant spin-up and spin-down populations are spatially separated and localized at the triangulene extension termini of **ZnPorT_2_** (Fig. S4.4) with some states extending over the Por core. Notably, *Ψ_U3_*, which is the molecule’s LUMO, is lower in energy than the SUMO (Fig. S4.4), indicating a potential SUMO–LUMO inversion [26]. However, the energy level arrangement predicted by DFT calculations (Fig. S4.4) is not consistent with the experimental observations (Fig. 3b). This leads us to consider a possible interfacial charge transfer between **ZnPorT_2_** and the underlying Au(111) surface.

The density of states (DOS) of **ZnPorT_2_** adsorbed on Au(111), calculated by UKS DFT at the PBE level (Fig. S4.1 and S4.2), suggests that the molecule is singly positively charged on the surface. To simulate this condition, we performed UKS DFT–PBE0 calculations for positively charged **ZnPorT_2_** in the gas phase (Fig. S4.5). Only one pair of SOMO/SUMO (labeled as *Ψ_CU2↑_* and *Ψ_CU3↓_* in Fig. S4.5) remains in the molecule due to the positive charging.

However, open-shell molecules typically exhibit many-body ground states, and DFT does not accurately capture their multireference character and static electron correlation [27]. To better elucidate the magnetic ground state and frontier orbital distributions, we thus performed CASSCF calculations on **ZnPorT_2_** in both neutral and singly positively charged states (for more details see Section S5). Considering both CAS(8,8) (representing complete active space containing 8 electrons and 8 orbitals) and CAS(12,12), two natural orbitals with electronic weight on the triangulene extensions show electron occupation close to 1 (Fig. S5.3). This confirms the diradical character of **ZnPorT_2_**. Dynamical correlation was accounted for through DLPNO-NEVPT2 corrections, yielding singlet–triplet energy gaps Δ*E*_ST_ of 30 and 41 meV for CAS(8,8) and CAS(12,12), respectively (Table S5.2). Removing one electron from **ZnPorT_2_** leads to **ZnPorT_2_^•^^+^**, which presents a single unpaired electron delocalized at the edge of the molecule backbone (Fig. S5.5 and S5.6).

Next, to interpret the d*I*/d*V* maps for **ZnPorT_2_**, we have calculated the Dyson orbitals [27] from the multireference wavefunction obtained from CASCI calculation on the positively charged **ZnPorT_2_^•^^+^** species (Fig. 3c, Fig. S5.7–S5.10). Five Dyson orbitals are obtained by considering six orbitals from electron addition and three orbitals from electron removal based on singly positively charged **ZnPorT_2_^•^^+^** (Fig. 3c). Simulated d*I*/d*V* maps [28] of Dyson orbitals show good agreement with experimental d*I*/d*V* maps (Fig. 3b).

We subsequently performed low-energy spin excitation spectroscopy to obtain the magnetic fingerprints of **ZnPorT_2_** (Fig. 3d). A Kondo resonance is expected, as the molecule still possesses *S* = 1/2 when singly positively charged on the surface. An ionic resonance at −3 mV with respect to the Fermi level is obtained, with a half-width at half-maximum (HWHM) of 19 meV from Frota fitting (Fig. 3d), which is found to be almost independent from temperature. Moreover, the energy of this low-energy resonance coincides with ionic resonance 2, shown in Fig. 3a. On the one hand, we speculate that the absence of a clear Kondo peak is due to the fact that it is obscured by the strong contributions of the SOMO- and SUMO-derived resonances 2 and 3 in this energy range [24]. On the other hand, the small Coulomb gap may enable electron hopping between these frontier orbitals, and thus **ZnPorT_2_** could enter the mixed valence regime with rapid fluctuations between several possible states, which destabilizes a ‘static’ open shell configuration [29–33].

**Figure 4.**
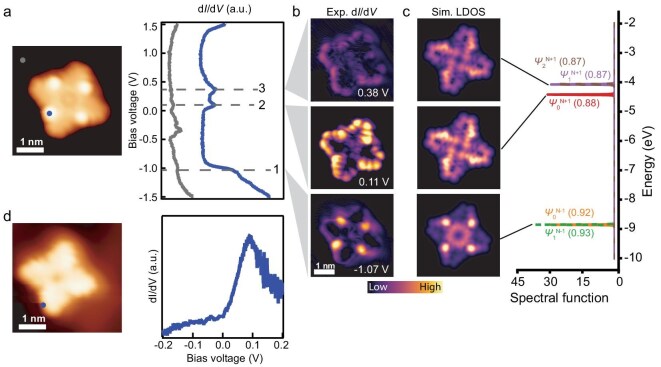
(a) STM image of **ZnPorT_4_** and corresponding d*I*/d*V* spectroscopy taken at the positions shown in the STM image. Lock-in amplitude: 20 mV. (b) d*I*/d*V* maps taken at resonances 1–3 from (a) in CC mode with a CO-functionalized tip. (c) Calculated many-body spectral function and corresponding simulated d*I*/d*V* maps of Dyson orbitals shown in Fig. S5.15 (electron removal) and Fig. S5.16 (electron injection) for doubly positively charged **ZnPorT_4_^2^^+^**. (d) STM image of **ZnPorT_4_** and d*I*/d*V* spectroscopy in the range of −0.2 to 0.2 V. Lock-in amplitude: 1 mV. Scanning parameters: (a) *U* = −1.5 V, *I* = 300 pA; (b) bias voltage is shown above each map; *I* = 300 pA; (d) *U* = −0.2 V, *I* = 1 nA.

Turning to **ZnPorT_4_**, three resonances are detected in the d*I*/d*V* spectra within the bias voltage range of −1.5 V to 1.5 V (Fig. 4a and b). At resonance 1, detected at −1.07 V, four pronounced states are observed at the ‘lifted’ triangulene corner. In contrast, at resonances 2 and 3, both located above the Fermi level, the dominant intensity is found along the zigzag edges of the triangulene extensions (Fig. 4b). In low-energy d*I*/d*V* spectra taken at the zigzag edge of **ZnPorT_4_** (Fig. 4d), a prominent resonance is detected at ∼0.1 V corresponding to resonance 2, as shown in Fig. 4a. The absence of resonance features at the Fermi level indicates that **ZnPorT_4_** holds a closed-shell configuration. To understand the experimentally observed orbital distributions and magnetic ground state of **ZnPorT_4_**, DFT–PBE0 calculations were subsequently performed (Fig. S4.14–S4.17). RKS DFT–PBE0 calculations predict four orbitals, contributed mainly by the zigzag edges of the triangulene extensions (Ψ_2_–Ψ_5_ in Fig. S4.14) with only the lowest energy orbital (i.e. Ψ_2_) fully occupied by two electrons. Under the UKS calculations, two of these four orbitals become spin-polarized, resulting in an antiferromagnetically coupled ground state with two unpaired electrons. The spin-polarized states are predominantly localized at two diagonally distributed triangulene extensions, whereas the other two triangulene extensions exhibit two degenerated states with the energy lying between SOMOs and SUMOs (Fig. S4.15). Similar to **ZnPorT_2_, ZnPorT_4_** also undergoes charge transfer with the substrate, as the experimentally observed zigzag edge states of **ZnPorT_4_** are above the Fermi level, in contrast to the UKS DFT results, in which one zigzag edge state is singly occupied. To account for the charge transfer in **ZnPorT_4_**, UKS DFT–PBE0 calculations on the singly and doubly positively charged **ZnPorT_4_** species were performed (Fig. S4.16 and S4.17). For two-electron positively charged **ZnPorT_4_** (i.e. **ZnPorT_4_^2+^**), the molecule possesses a closed-shell configuration with the orbitals contributed by triangulene extensions fully empty, which agrees with the experimental observations. We further take into account the transition via virtual charge states by employing multireference Dyson orbitals. The orbital contributions to each experimentally detected ionic resonance are consistent with DFT calculations. In this case, the single-electron DFT orbitals are sufficient to describe the electronic state of **ZnPorT_4_^2^^+^** on the surface (Fig. 4c, Fig. S5.13–S5.17).

The study on **ZnPorT_2_** and **ZnPorT_4_** provides a coherent description of the interplay between geometric symmetry and their electronic configurations in the single‐particle picture. **ZnPorT_2_** exhibits 2-fold symmetry, with two unpaired *π* electrons localized at the zigzag termini of its triangulene extension. Due to the intramolecular hybridization, the nominally degenerate zero-energy states split by a small gap [34]. In the 4-fold symmetric **ZnPorT_4_**, the number of symmetry-equivalent triangulene extensions available for hosting the two unpaired *π* electrons increases to four. Similarly to **ZnPorT_2_**, the two spin-carrying orbitals hybridize and undergo energy splitting, whereas the other two orbitals remain energy degenerated. Unless the geometry of **ZnPorT_4_** undergoes geometric symmetry breaking to reduce its symmetry from 4-fold to 2-fold, the spin-hosting orbitals remain arbitrary among the four zigzag-edge states (Ψ_2_–Ψ_5_ in Fig. S4.14), potentially leading to a frustrated electronic phase. On Au(111), however, **ZnPorT_4_** adopts a closed-shell configuration due to a two-electron charge transfer to the substrate, which is an alternative stabilization mechanism that circumvents the need for geometric symmetry breaking. Even though a clear charge transfer is confirmed by DFT and many-body calculations, we are aware that additional physics processes, such as charge fluctuations in a mixed valence regime, may also contribute to the absence of magnetic fingerprints in low-energy spectroscopy in both **ZnPorT_2_** and **ZnPorT_4_**, which requires further comprehensive studies and theoretical models.

## CONCLUSION

In conclusion, we demonstrate the successful on-surface synthesis of [3]triangulene *π*-extended Por nanostructures **ZnPorT_2_** and **ZnPorT_4._** The formation of such species was maximized using a rapid heating protocol with short annealing time, which efficiently limits the diffusion of such reactive Por nanostructures on the Au(111) surface, thereby minimizing their undesired lateral fusion. Both **ZnPorT_2_** and **ZnPorT_4_** feature diradical electronic configurations with open-shell singlet ground states. The comparison of **ZnPorT_2_** and **ZnPorT_4_** highlights the crucial role of geometric symmetry in determining their electronic configurations. In both systems, interfacial charge transfer from the molecules to the substrate takes place and leads to a one-electron donation for **ZnPorT_2_**, and a two-electron donation for **ZnPorT_4_**, affording open-shell **ZnPorT_2_^•^^+^** and closed-shell **ZnPorT_4_^2+^** species, respectively. Notably, despite the interfacial charge transfer, the multireference character of the frontier orbitals is maintained in **ZnPorT_2_^•^^+^** and rationalized with the assistance of DFT and many-body calculations. This study advances the preparation of Por-NG hybrids and clarifies their magnetic properties, providing a basis for designing tailored molecular spin systems for future Por-NG hybrid quantum devices.

## METHODS

### Sample preparation

The Au(111) epitaxially grown on a mica substrate (Phasis Sarl) was used in this work. Au(111)/mica was cleaned by several cycles of argon ion sputtering at 5.0 × 10^−6^ mbar, followed by annealing to 690 K for 10 min. The precursors **ZnPor(dmpa)_2_** and **ZnPor(dmpa)_4_** were sublimated on a clean Au(111) kept at RT or 570 K, using standard quartz crucible with a temperature of 720 and 780 K, respectively. The different Por-triangulene hybrid structures, **ZnPorT_2_** and **ZnPorT_4_**, were synthesized by further annealing the sample to 570 K to trigger the intramolecular cyclodehydrogenative coupling.

### Scanning probe microscopy measurements

STM and nc-AFM were carried out in an ultrahigh vacuum system with a base pressure lower than 2 × 10^−10^ mbar. A commercial STM/nc-AFM (Scienta-Omicron GmbH) in a separate chamber and equipped with Matrix electronics and Nanonis electronics was used to perform the experiments at *T* = 4.5 K. A qPlus tuning fork sensor [35] with a chemically etched tungsten tip was used. The sensor has a resonance frequency of *f*_0_ ∼ 24–27 kHz and *Q* ∼ 47k and was operated with an oscillation amplitude of *A*_osc_ ≤ 44 pm. Nc-AFM images were acquired in CH mode with a CO-functionalized tip. The definition of the tip position is given in the corresponding figure captions. A CO molecule was picked up by applying a 1.5-V voltage pulse at the tip position of 0.1 V, 100 pA or by scanning the surface using 0.01 V, 100 pA. The nc-AFM measurements were recorded using Omicron Matrix electronics and HF2LI phase-locked loop (PLL) from Zurich Instruments. STS was performed using an HF2LI Lock-in Amplifier from Zurich Instruments and Nanonis Lock-in Amplifier with a modulation frequency between 681 and 730 Hz. The modulation amplitude is provided in the corresponding figure caption. The bias voltage is applied to the sample, and the tip is grounded. All bias voltages in the manuscript refer to the sample bias. The data were processed by using WaveMetrics Igor Pro software.

## Supplementary Material

nwag155_Supplemental_File The supplementary data are available at *NSR* online, including detailed sample preparations, experimental and computational methods, synthetic procedures of **ZnPor(dmpa)_2_** and **ZnPor(dmpa)_4_**, associated solution characterization data, additional STM/STS, nc-AFM data, DFT and many-body calculations.

## References

[1] Pavliček N, Mistry A, Majzik Z et al. Synthesis and characterization of triangulene. Nat Nanotechnol 2017; 12: 308–11. 10.1038/nnano.2016.305 28192389

[2] Mishra S, Beyer D, Eimre K et al. Synthesis and characterization of *π*-extended triangulene. J Am Chem Soc 2019; 141: 10621–5. 10.1021/jacs.9b05319 31241927

[3] Mishra S, Xu K, Eimre K et al. Synthesis and characterization of [7]triangulene. Nanoscale 2021; 13: 1624–8. 10.1039/D0NR08181G 33443270

[4] Mishra S, Beyer D, Eimre K et al. Topological frustration induces unconventional magnetism in a nanographene. Nat Nanotechnol 2020; 15: 22–8. 10.1038/s41565-019-0577-9 31819244

[5] Song S, Pinar Solé A, Matěj A et al. Highly entangled polyradical nanographene with coexisting strong correlation and topological frustration. Nat Chem 2024; 16: 938–44. 10.1038/s41557-024-01453-9 38374456

[6] Mishra S, Yao X, Chen Q et al. Large magnetic exchange coupling in rhombus-shaped nanographenes with zigzag periphery. Nat Chem 2021; 13: 581–6. 10.1038/s41557-021-00678-2 33972756

[7] Li J, Sanz S, Corso M et al. Single spin localization and manipulation in graphene open-shell nanostructures. Nat Commun 2019; 10: 200. 10.1038/s41467-018-08060-6 30643120 PMC6331630

[8] Wang T, Berdonces-Layunta A, Friedrich N et al. Aza-triangulene: on-surface synthesis and electronic and magnetic properties. J Am Chem Soc 2022; 144: 4522–9. 10.1021/jacs.1c12618 35254059 PMC8931755

[9] Turco E, Bernhardt A, Krane N et al. Observation of the magnetic ground state of the two smallest triangular nanographenes. JACS Au 2023; 3: 1358–64. 10.1021/jacsau.2c00666 37234116 PMC10207087

[10] Su J, Telychko M, Hu P et al. Atomically precise bottom-up synthesis of *π*-extended [5]triangulene. Sci Adv 2019; 5: eaav7717. 10.1126/sciadv.aav7717 31360763 PMC6660211

[11] Su J, Fan W, Mutombo P et al. On-surface synthesis and characterization of [7]triangulene quantum ring. Nano Lett 2021; 21: 861–7. 10.1021/acs.nanolett.0c04627 33305570

[12] Mishra S, Beyer D, Eimre K et al. Collective all-carbon magnetism in triangulene dimers. Angew Chem Int Ed 2020; 59: 12041–7. 10.1002/anie.202002687 PMC7383983 32301570

[13] Mishra S, Catarina G, Wu F et al. Observation of fractional edge excitations in nanographene spin chains. Nature 2021; 598: 287–92. 10.1038/s41586-021-03842-3 34645998

[14] Chen Q, Lodi A, Zhang H et al. Porphyrin-fused graphene nanoribbons. Nat Chem 2024; 16: 1133–40. 10.1038/s41557-024-01477-1 38459234 PMC11230900

[15] He Y, Garnica M, Bischoff F et al. Fusing tetrapyrroles to graphene edges by surface-assisted covalent coupling. Nat Chem 2017; 9: 33–8. 10.1038/nchem.2600 27995925

[16] Kadish KM, Smith KM, Guilard R. The Porphyrin Handbook. Burlington: Academic Press, 2000.

[17] Sun Q, Mateo LM, Robles R et al. Magnetic interplay between *π*-electrons of open-shell porphyrins and d-electrons of their central transition metal ions. Adv Sci 2022; 9: 2105906. 10.1002/advs.202105906 PMC9259720 35302718

[18] Robles R, Edalatmanesh S, Sun Q et al. Tailoring *π*–d magnetic interactions in metallated porphyrin nanotapes. Angew Chem Int Ed 2025; 64: e15342. 10.1002/anie.202515342 PMC12723466 41186057

[19] Tenorio M, Lozano M, Cerna L et al. Coordinative self-assembly of *π*-electron magnetic porphyrins. Angew Chem Int Ed 2025; 64: e202420572. 10.1002/anie.202420572 39642287

[20] Lieb EH . Two theorems on the Hubbard model. Phys Rev Lett 1989; 62: 1201–4. 10.1103/PhysRevLett.62.1201 10039602

[21] Clar E . The Aromatic Sextet. London, New York: J. Wiley, 1972.

[22] Sun Q, Mateo LM, Robles R et al. Inducing open-shell character in porphyrins through surface-assisted phenalenyl *π*-extension. J Am Chem Soc 2020; 142: 18109–17. 10.1021/jacs.0c07781 32985889

[23] Zhao Y, Jiang K, Li C et al. Precise control of *π*-electron magnetism in metal-free porphyrins. J Am Chem Soc 2020; 142: 18532–40. 10.1021/jacs.0c07791 32959653

[24] Mishra S, Beyer D, Berger R et al. Topological defect-induced magnetism in a nanographene. J Am Chem Soc 2020; 142: 1147–52. 10.1021/jacs.9b09212 31904953

[25] Wang T, Fan Q, Zhu J. Steering on-surface reactions by kinetic and thermodynamic strategies. J Phys Chem Lett 2023; 14: 2251–62. 10.1021/acs.jpclett.3c00001 36821589

[26] Li Z, Liu X, Bao Q et al. The SUMO (singly unoccupied molecular orbital)-LUMO (lowest unoccupied molecular orbital) inversion radicals. J Am Chem Soc 2025; 147: 1452–7. 10.1021/jacs.4c16614 39737560

[27] Kumar M, Soler-Polo D, Lozano M et al. Multireference theory of scanning tunneling spectroscopy beyond one-electron molecular orbitals: can we image molecular orbitals? J Am Chem Soc 2025; 147: 24993–5003. 10.1021/jacs.5c08166 40600652 PMC12272701

[28] Krejčí O, Hapala P, Ondráček M et al. Principles and simulations of high-resolution STM imaging with a flexible tip apex. Phys Rev B 2017; 95: 045407. 10.1103/PhysRevB.95.045407

[29] Pixley JH, Kirchner S, Ingersent K et al. Kondo destruction and valence fluctuations in an Anderson model. Phys Rev Lett 2012; 109: 086403. 10.1103/PhysRevLett.109.086403 23002763

[30] Lee M, Choi M-S. Mixed-valence transition in a quantum dot coupled to superconducting and spin-polarized leads. Phys Rev B 2019; 99: 075161. 10.1103/PhysRevB.99.075161

[31] Goldhaber-Gordon D . From the Kondo regime to the mixed-valence regime in a single-electron transistor. Phys Rev Lett 1998; 81: 5225–8. 10.1103/PhysRevLett.81.5225

[32] Jacob D . Renormalization of single-ion magnetic anisotropy in the absence of the Kondo effect. Phys Rev B 2018; 97: 075428. 10.1103/PhysRevB.97.075428

[33] Biswas K, Janeiro J, Gallardo A et al. Designing highly delocalized solitons by harnessing the structural parity of *π*-conjugated polymers. Nat Synth 2025; 4: 233–42. 10.1038/s44160-024-00665-8

[34] Jacob D, Fernández-Rossier J. Theory of intermolecular exchange in coupled spin-½ nanographenes. Phys Rev B 2022; 106: 205405. 10.1103/PhysRevB.106.205405

[35] Giessibl FJ . The qPlus sensor, a powerful core for the atomic force microscope. Rev Sci Instrum 2019; 90: 011101. 10.1063/1.5052264 30709191

