# Supplementary material for: On-surface synthesis and interfacial charge redistribution of open-shell [3]triangulene-fused porphyrins on Au(111)
Source: Natl Sci Rev. 2026 Mar 11;13(7):nwag155. doi: 10.1093/nsr/nwag155 (PMC13098376; doi:10.1093/nsr/nwag155)
Supplement: nwag155_Supplemental_File — The supplementary data are available at NSR online, including detailed sample preparations, experimental and computational methods, synthetic procedures of ZnPor(dmpa)2 and ZnPor(dmpa)4, associated solution characterization data, additional STM/STS, nc-AFM data, DFT and many-body calculations. [file nwag155_supplemental_file.pdf]

Supplementary Information for

On-surface synthesis and interfacial charge redistribution of open-shell [3]triangulene-fused porphyrins on Au(111)

Miguel Martínez García,[1,2,§] Feifei Xiang,[3,4,§,*] Andres Ortega-Guerrero,[3] Manish Kumar,[5] Nicolò Bassi,[3] Carlo A. Pignedoli,[3] Pascal Ruffieux,[3] Tomas Torres,[1,2,6,*] Pavel Jelínek,[5,*] Roman Fasel,[3,7] and Giovanni Bottari[1,2,6,*]

[1] Departamento de Química Orgánica, Universidad Autónoma de Madrid, Campus de Cantoblanco, 28049 Madrid, Spain

[2] IMDEA-Nanociencia, Campus de Cantoblanco, 28049 Madrid, Spain

[3] nanotech@surfaces Laboratory, Empa - Swiss Federal Laboratories for Materials Science and Technology, 8600 Dübendorf, Switzerland

[4] School of Advanced Manufacturing Engineering, Nanjing University, Suzhou, 215163, China

[5] Institute of Physics, Czech Academy of Sciences, Prague 16200, Czech Republic

[6] Institute for Advanced Research in Chemical Sciences (IAdChem), Universidad Autónoma de Madrid, 28049, Madrid, Spain

[7] Department of Chemistry, Biochemistry and Pharmaceutical Sciences, University of Bern, 3012 Bern, Switzerland

*Corresponding authors. E-mails: feifei.xiang@nju.edu.cn, tomas.torres@uam.es, jelinekp@fzu.cz, giovanni.bottari@uam.es

[§]These authors contributed equally to this work.

Table of content

S1 **General: Materials and Methods** ... 4

Computational details ... 4

Synthetic details ... 5

S2 **Synthetic procedures and spectroscopic data** ... 6

Solution-based synthesis and characterization of ZnPor(dmpa)$_2$ and ZnPor(dmpa)$_4$ ... 6

Synthesis and characterization of 9-(2,6-dimethylphenyl)anthracene 1 ... 6

Synthesis and characterization of 9-bromo-10-(2,6-dimethylphenyl)anthracene 2 ... 7

Synthesis and characterization of 10-(2,6-dimethylphenyl)anthracene-9-carbaldehyde 3 10

Synthesis and characterization of *meso*-H-dipyrromethane *meso*-H-DPM ... 13

Synthesis and characterization of 5,15-bis(10-(2,6-dimethylphenyl)anthracen-9-yl)-Zn(II) porphyrin ZnPor(dmpa)$_2$ ... 17

Synthesis and characterization of 5,10,15,20-tetrakis(10-(2,6-dimethylphenyl)anthracen-9-yl)porphyrin H$_2$Por(dmpa)$_4$ ... 20

Synthesis and characterization of 5,10,15,20-tetrakis(10-(2,6-dimethylphenyl)anthracen-9-yl)-Zn(II)porphyrin ZnPor(dmpa)$_4$ ... 23

S3 **Resonance structures of ZnPorT$_2$ and ZnPorT$_4$** ... 27

S4 **Supplementary STM measurements and DFT calculations** ... 31

S5 **Many-body calculations** ... 47

S6 **Charge analysis** ... 56

S7 **References** ... 58

List of abbreviations:

AcOEt = Ethyl acetate

ATR = Attenuated total reflectance

DCM = Dichloromethane

DCTB = *trans*-2-[3-(4-*tert*-butylphenyl)-2-methyl-2-propenylidene]malononitrile

DDQ = 2,3-dichloro-5,6-dicyano-1,4-benzoquinone

DMF = Dimethylformamide

FT-IR = Fourier-transform infrared spectroscopy

GC-EI-TOF = Gas chromatography-electron ionization-time of flight

n-BuLi = *n*-Butyllithium

MALDI-TOF = Matrix-assisted laser desorption/ionization-time of flight

MeOH = Methanol

MS = Mass spectrometry

NMR = Nuclear magnetic resonance

R_f = Retention factor

THF = Tetrahydrofuran

S1 General: Materials and Methods

Computational details

Density functional theory (DFT)

Density functional theory (DFT) calculations were performed to model the electronic properties of **ZnPorT$_2$** and **ZnPorT$_4$** molecules using AiiDAlab [1] apps based on AiiDA [2] workflows for the CP2K [3] code (version v2024.1). The geometry of these two molecules was firstly optimized in gas phase and on Au(111) using PBE functional. For the AFM simulations we used an AiiDAlab app leveraging the probe particle code from P. Hapala [4]. Then the PBE0 exchange-correlation functional [5] was employed to calculate the energy spectrum and the corresponding molecular orbitals of both molecules in different electronic configurations in gas phase. The orbital transformation (OT) method [6] was utilized, with plane-wave and relative energy cutoffs set at 700 Ry and 70 Ry, respectively, and a 5-level multigrid mapping. The Martyna-Tuckerman Poisson solver algorithm [7] was used to solve the Poisson equation. Goedecker-Teter-Hutter (GTH) pseudopotentials [8] for the PBE0 functional (GTH-PBE0) were employed, along with mixed Gaussian and plane-wave basis sets [9]. The TZV2P MOLOPT basis set was used to describe the atoms (TZV2P-MOLOPT-PBE0-GTH). Additionally, the auxiliary density matrix methods for Hartree−Fock exchange calculations [10] were utilized with the MOLOPT-ADMM basis functions (ADMM-TZP).

Complete active space self-consistent field (CASSCF)

The electronic properties of the neutral and positively charged **ZnPorT$_2$** complex were investigated using various levels of theory within the ORCA 6.0.1 software package [11]. DFT calculations were performed using the def2-TZVP basis set, employing broken-symmetry (BS) approaches to describe the open-shell character of the systems. Two exchange-correlation functionals were considered: PBE0 and M06-2X.

To assess the multiconfigurational nature of the ground states, Complete Active Space Self-Consistent Field (CASSCF) [12] calculations were performed on both the neutral and cationic species using CAS (12,12) and CAS (11,12) active spaces, respectively. The Resolution-of-Identity for the Coulomb and exchange integrals (RIJK) approximation [13] was employed to accelerate the SCF convergence, utilizing the def2/JK auxiliary basis for exchange fitting and the def2-TZVP/C auxiliary basis for Coulomb integrals. Initial guess orbitals were generated from quasi-restricted molecular orbitals obtained from the preceding M06-2X DFT calculations.

Spin states and low-lying excitations were characterized through State-Averaged CASSCF (SA-CASSCF) calculations for the neutral molecule. To account for dynamic electron

correlation, the CASSCF energies were further refined using the Domain-Based Local Pair Natural Orbital N-Electron Valence Second-Order Perturbation Theory (DLPNO-NEVPT2) approach [14].

Synthetic details

Chemicals and solvents were purchased from commercial suppliers (Aldrich, BLDpharm, TCI, Thermo Scientific Chemicals and Scharlab) and used without further purification. All dry solvents were freshly distilled under argon over an appropriate drying agent before use. Column chromatography was carried out on Silica gel VWR-60 (40-63 μm). Analytical TLC was performed on aluminum sheets precoated with silica gel 60 F-254 from Merck. Size Exclusion Chromatography was carried out on Bio-Beads S-X1 Support (1 % cross-linkage, 40–80 μm) from Bio-Rad.

^{1}H- and ^{13}C-NMR spectra were recorded with a Bruker DPX 400 MHz instrument. Chemical shifts values (δ) are referred to the corresponding deuterated solvent ($CDCl_3$ or THF-d_8).

UV/vis experiments were carried out by using quartz cells with a 1 cm optical path length in a Varian Cary 50 UV spectrophotometer. IR spectra were recorded on a Bruker ALPHA Platinum-ATR system.

MALDI-TOF MS spectra were obtained in a Bruker ULTRAFLEX III (MALDI-TOF/TOF) spectrometer. GC-MS spectra were obtained from a Waters GCT Agilent Technologies 6890N spectrometer.

S2 Synthetic procedures and spectroscopic data

Solution-based synthesis and characterization of ZnPor(dmpa)2 and ZnPor(dmpa)4

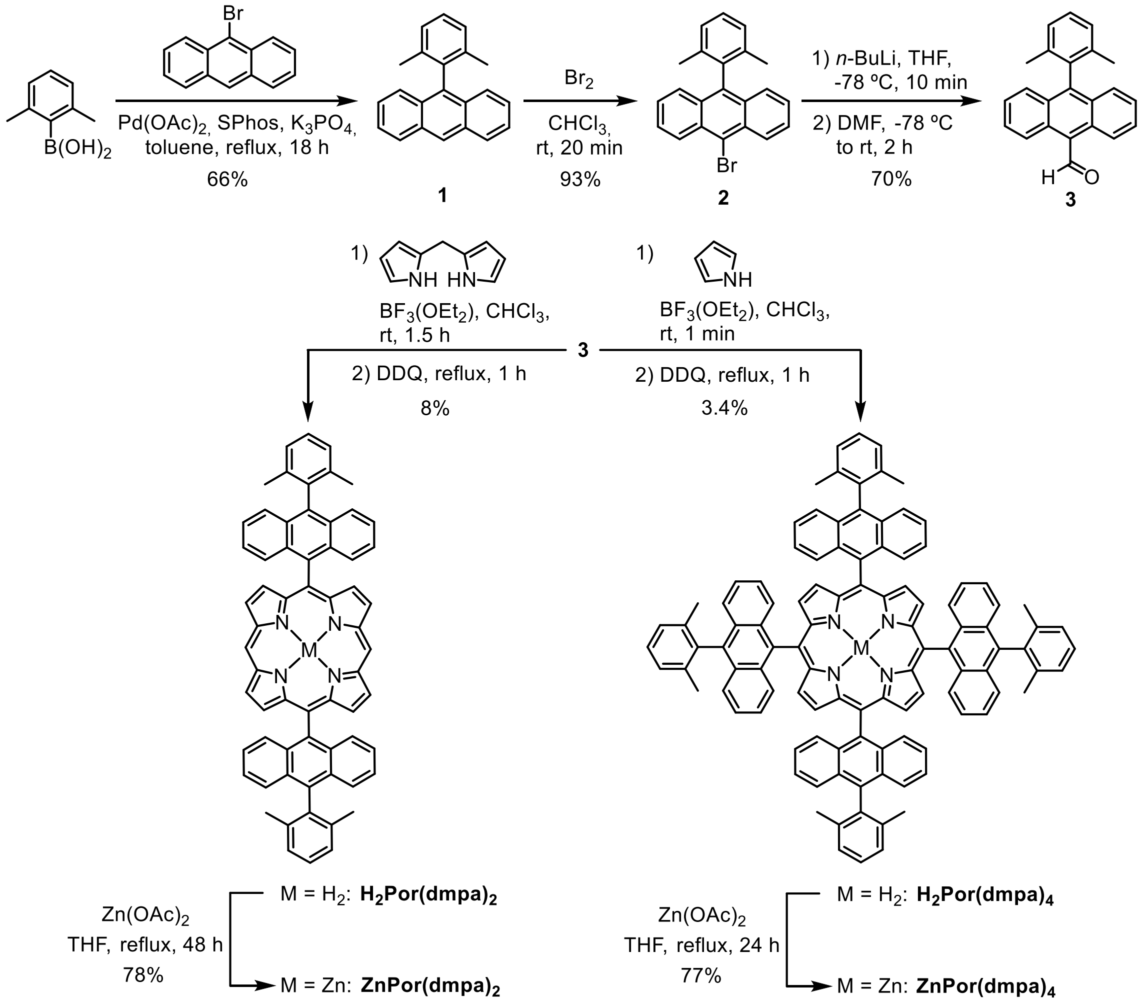

Supplementary Figure 2.1. Synthetic routes towards **ZnPor(dmpa)2** and **ZnPor(dmpa)4**.

Synthesis and characterization of 9-(2,6-dimethylphenyl)anthracene 1

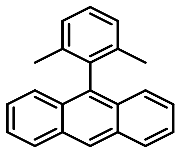

The synthesis of **1** was carried out using a modified procedure from literature [15].

A 100 mL flame-dried Schlenk was charged with 9-bromoanthracene (3.81 g, 15 mmol), 2,6-dimethylphenylboronic acid (3.38 g, 22.5 mmol, 1.5 eq.), $Pd(OAc)_2$ (101 mg, 0.45 mmol, 0.03 eq.), SPhos (369 mg, 0.90 mmol, 0.06 eq.), K_3PO_4 (7.96 g, 37.5 mmol, 2.5 eq.), and anhydrous toluene (100 mL). The resulting mixture was subjected to three freeze-pump-thaw cycles and then stirred at 105 °C overnight. After that, the reaction mixture was cooled to room temperature and poured over water (50 mL). The organic layer was separated, and the aqueous layer was

extracted with DCM (2 × 30 mL). The organic extracts were combined and dried over anhydrous $MgSO_4$, filtered, and concentrated in vacuum. The resulting mixture was subjected to column chromatography using pure *n*-heptane as eluent, obtaining the pure product as a white solid (2.80 g, 66 %).

^{1}H- and ^{13}C-NMR data were consistent with the ones reported in the literature [16].

Synthesis and characterization of 9-bromo-10-(2,6-dimethylphenyl)anthracene 2

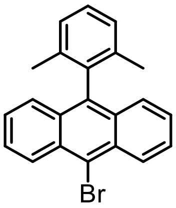

In a flame-dried 100 mL round-bottom flask **1** (1.41 g, 5.00 mmol) was dissolved in 20 mL of $CHCl_3$. To the resulting solution, while stirring, was added dropwise over 5 minutes Br_2 (0.28 mL, 5.5 mmol, 1.1 eq.). The reaction mixture was allowed to stir for 20 minutes at room temperature and then quenched with saturated $Na_2S_2O_3$ (15 mL). The mixture was extracted with DCM (2 × 25 mL) and the combined organic layers were dried over anhydrous $MgSO_4$, filtered and concentrated under reduced pressure. The solid residue was suspended in MeOH, sonicated, filtered and washed with more MeOH (2 × 5 mL) affording **2** as a light-yellow powder (1.68 g, 93 %).

^{1}H-NMR (400 MHz, $CDCl_3$): δ = 8.62 (d, *J* = 8.8 Hz, 2H), 7.60 (ddd, *J* = 8.8, 6.5, 1.3 Hz, 2H), 7.47 (d, *J* = 8.5 Hz, 2H), 7.40 – 7.34 (m, 3H), 7.27 (d, *J* = 7.6 Hz, 2H), 1.73 (s, 6H); **^{13}C-NMR** (100 MHz, $CDCl_3$): δ = 137.8, 137.3, 136.6, 130.7, 130.5, 128.3, 128.1, 127.7, 127.2, 126.4, 126.1, 122.5, 20.2; **GC-EI-TOF MS**: *m/z* (% intensity) = 360.05 – 364.06 m/z $[M]^+$ (100 %); **FT-IR (ATR)**: ν (cm^{-1}) = 3063, 3040, 2917, 1462, 1438, 1377, 1332, 1258, 1025, 934, 878, 757, 649, 617.

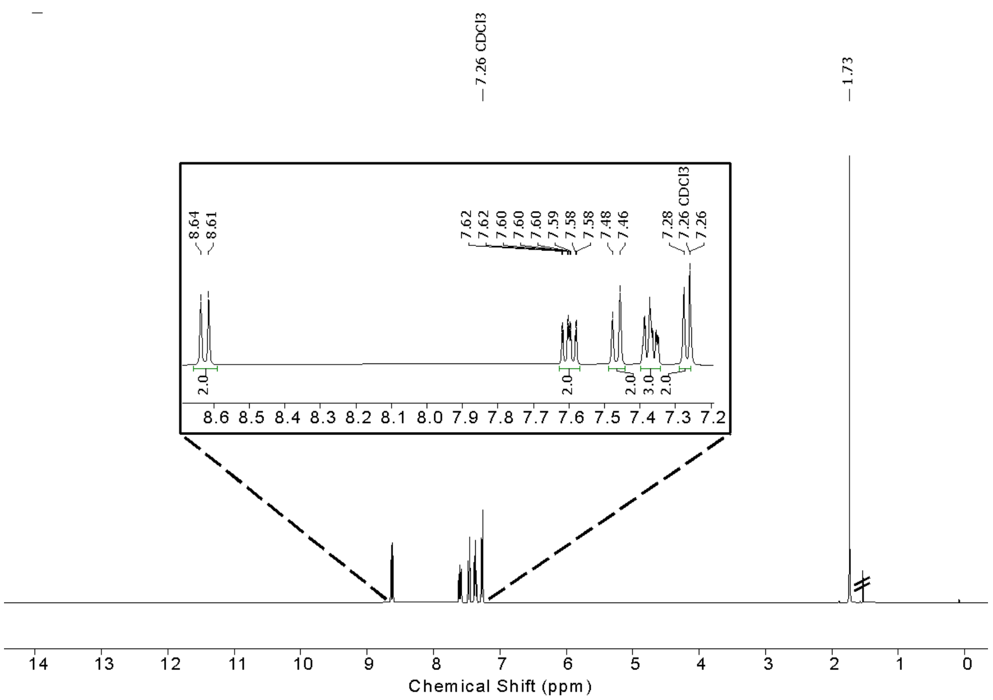

Supplementary Figure 2.2. ^{1}H-NMR spectrum of **2** in $CDCl_3$ (≠ = residual solvent signals).

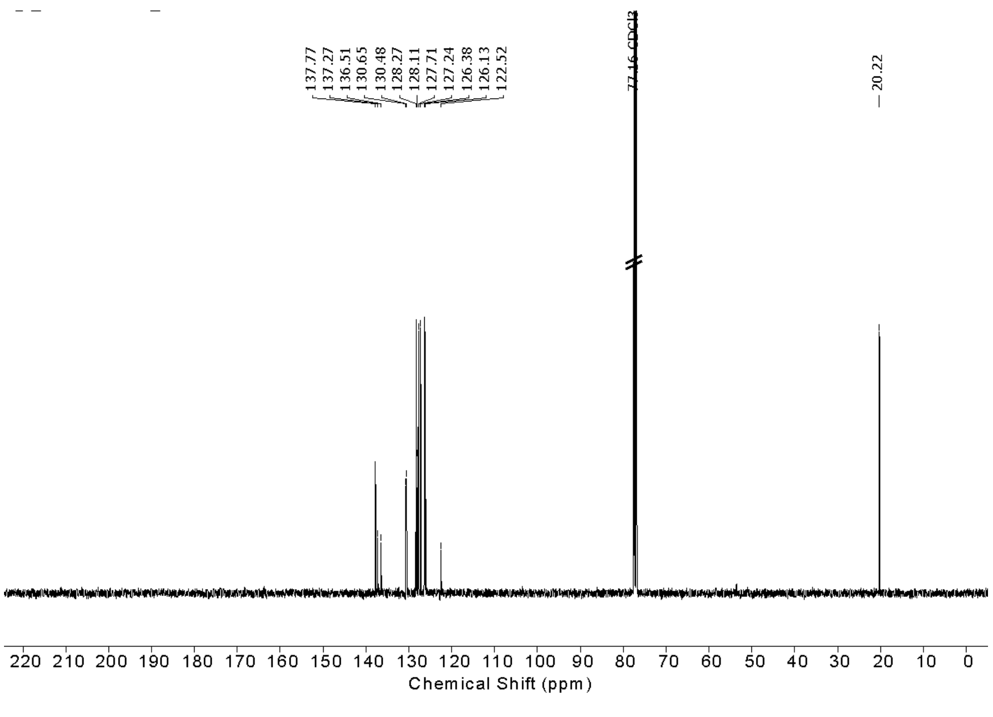

Supplementary Figure 2.3. ^{13}C-NMR spectrum of **2** in $CDCl_3$ (≠ = residual solvent signals).

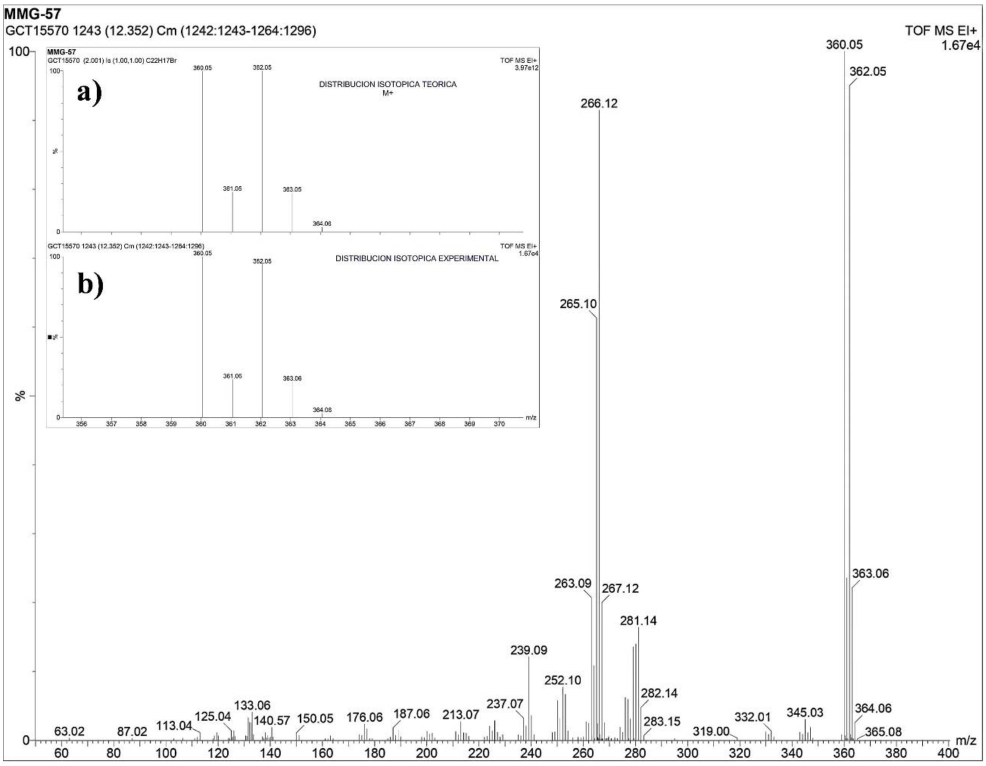

Supplementary Figure 2.4. GC-EI mass spectrum of **2**. Inset: (a) calculated isotopic pattern for **2**. (b) experimental isotopic distribution of the GC-EI peaks between 360.05 and 364.06 m/z.

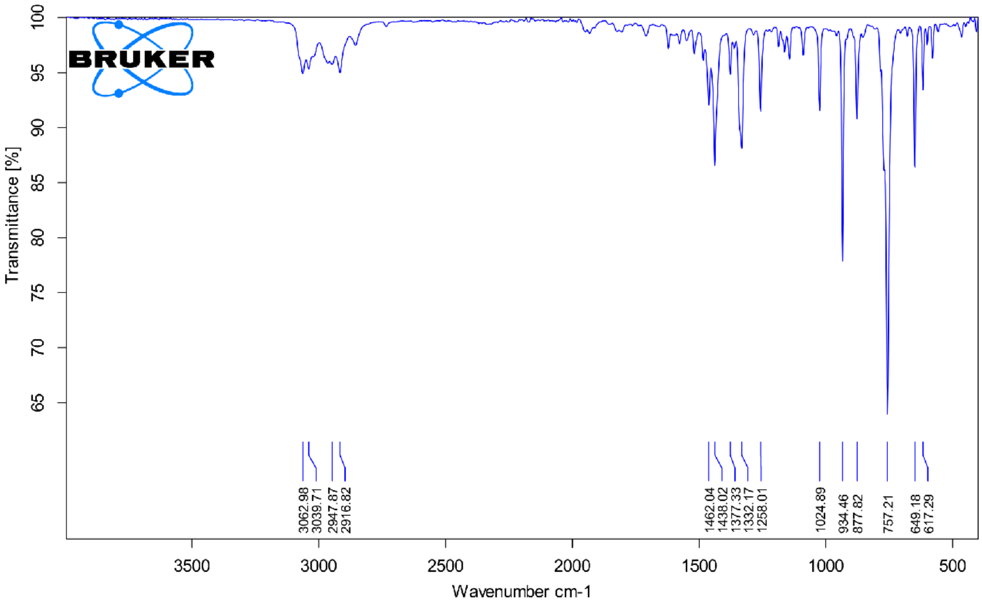

Supplementary Figure 2.5. FT-IR spectrum of **2**.

Synthesis and characterization of 10-(2,6-dimethylphenyl)anthracene-9-carbaldehyde 3

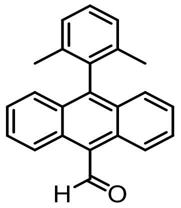

Compound **2** (1.90 g, 5.27 mmol) was charged in a flame-dried 50 mL round-bottom flask and dissolved in freshly distilled THF (20 mL) under argon atmosphere. This solution was cooled to –78 ºC and *n*-BuLi (2.5 M in *n*-hexanes, 2.55 mL, 6.32 mmol, 1.2 eq.) was added dropwise while stirring. After 10 minutes, dry DMF (2 mL, excess) was slowly added, and the reaction mixture was stirred for 30 minutes at –78 ºC and then for 1 hour at room temperature. After that, the reaction is quenched with NH_4Cl sat. (40 mL) and the organic layer was extracted with Et_2O (3 × 20 mL). The combined organic layers were washed with water and brine, dried over $MgSO_4$, filtered and concentrated in vacuum. The resulting mixture was purified by column chromatography using *n*-heptane/DCM (1:1) as eluent, where the second fraction (R_f = 0.41) was isolated and concentrated. Aldehyde **3** was obtained as an intense yellow solid (1.14 g, 70 %).

^{1}H-NMR (400 MHz, $CDCl_3$): δ = 11.61 (s, 1H), 9.05 (d, *J* = 8.9 Hz, 2H), 7.68 (ddd, *J* = 8.9, 6.6, 1.3 Hz, 2H), 7.54 (d, *J* = 8.7 Hz, 2H), 7.43 – 7.37 (m, 3H), 7.28 (d, *J* = 7.8 Hz, 2H), 1.73 (s, 6H); **^{13}C-NMR** (100 MHz, $CDCl_3$): δ = 193.6, 144.9, 137.3, 137.2, 132.1, 129.4, 129.0, 128.4, 127.8, 127.0, 126.2, 125.0, 124.0, 20.1; **GC-EI-TOF MS**: *m/z* (% intensity) = 309.13 – 312.15 m/z $[M]^+$ (100 %); **FT-IR (ATR)**: ν (cm^{-1}) = 3064, 1676, 1557, 1464, 1439, 1272, 1187, 1156, 1046, 1029, 922, 775, 761, 719, 653, 614, 574.

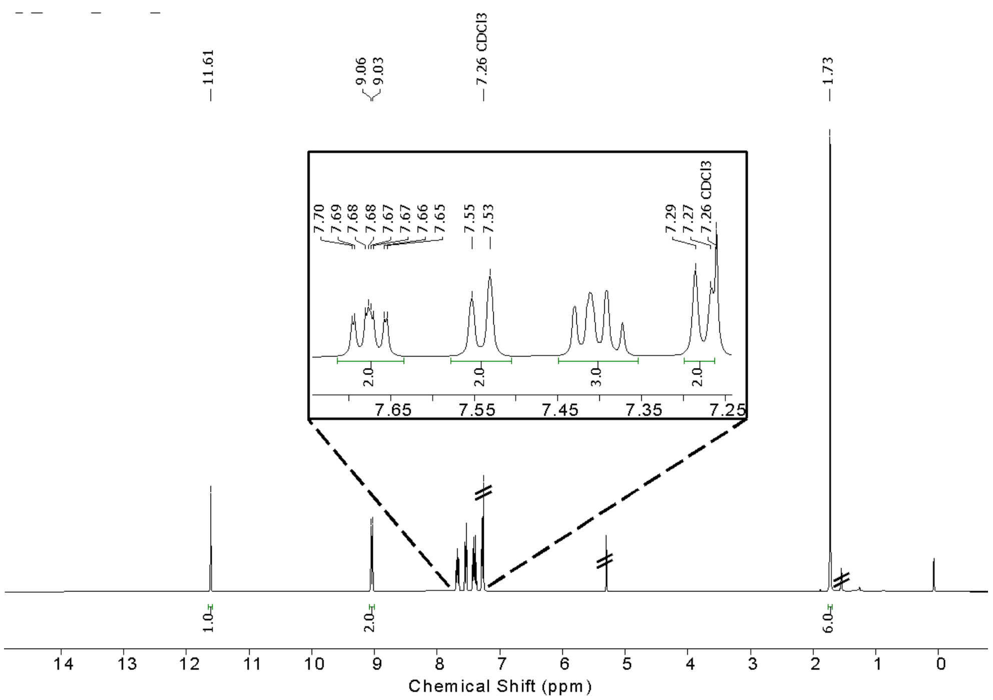

Supplementary Figure 2.6. ^{1}H-NMR spectrum of **3** in $CDCl_3$ (≠ = residual solvent signals).

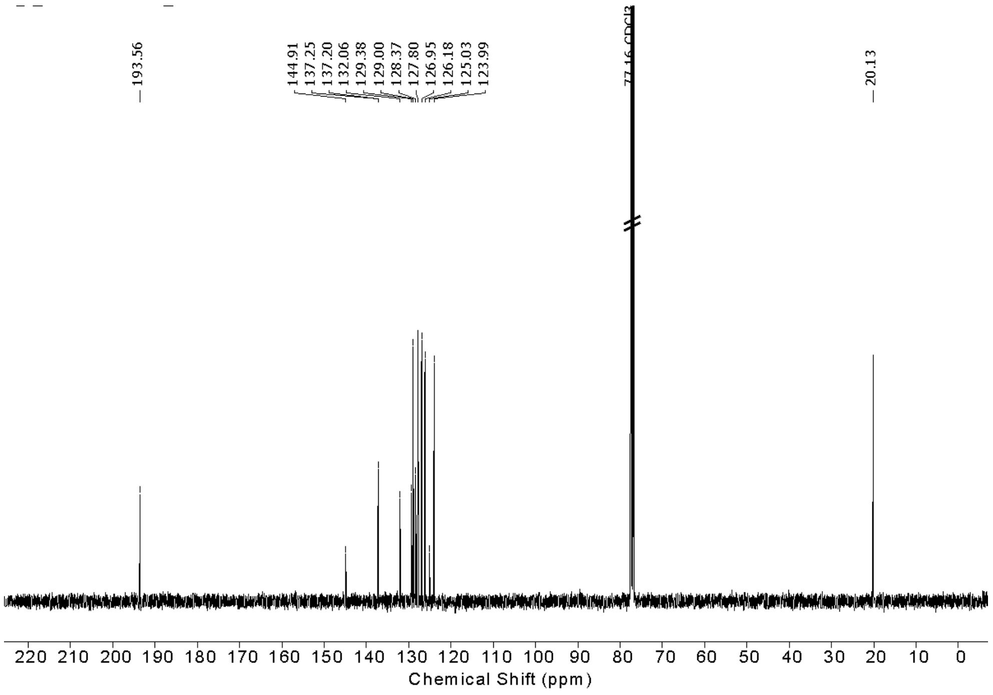

Supplementary Figure 2.7. ^{13}C-NMR spectrum of **3** in $CDCl_3$ (≠ = residual solvent signals).

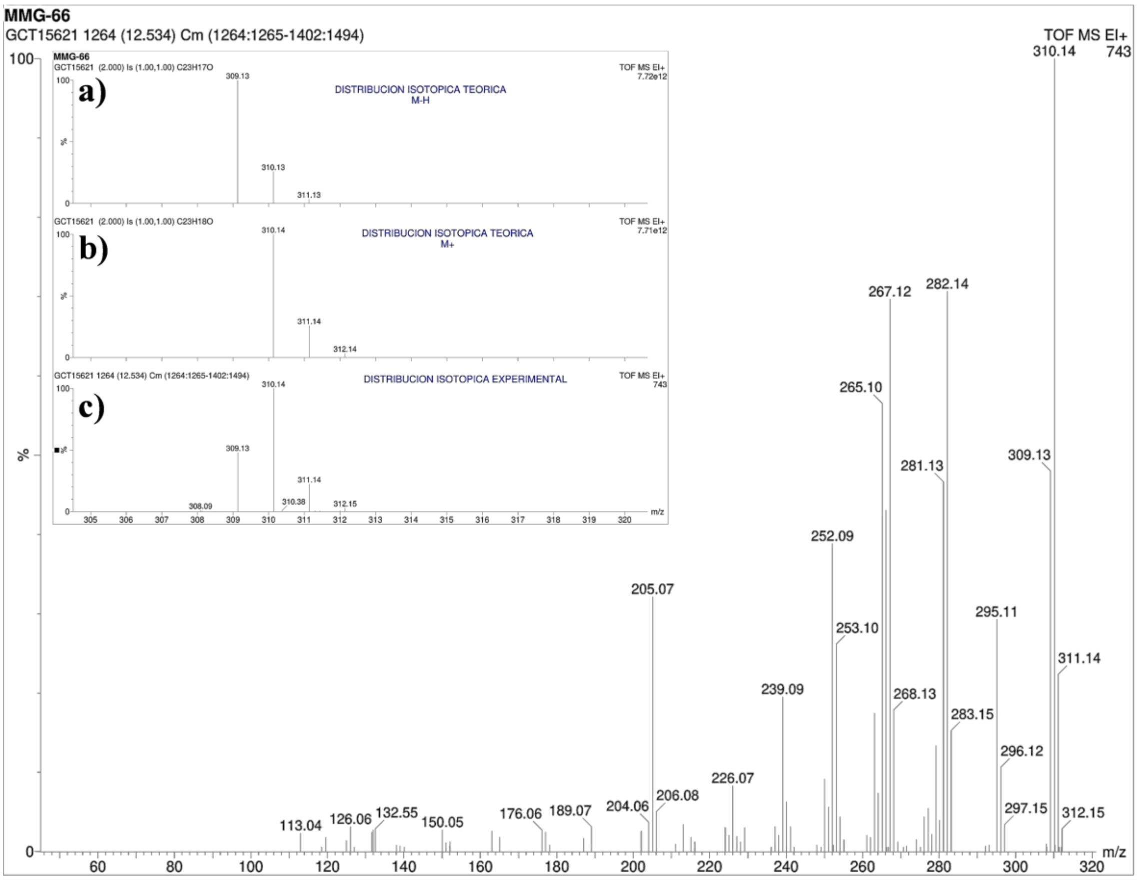

Supplementary Figure 2.8. GC-EI mass spectrum of **3**. Inset: (a) Calculated isotopic pattern for $C_{23}H_{17}O$ $[M–H]^+$. (b) Calculated isotopic pattern for $C_{23}H_{18}O$ $[M]^+$. (c) Experimental isotopic distribution of the GC-EI peaks between 309.13 and 312.15 m/z.

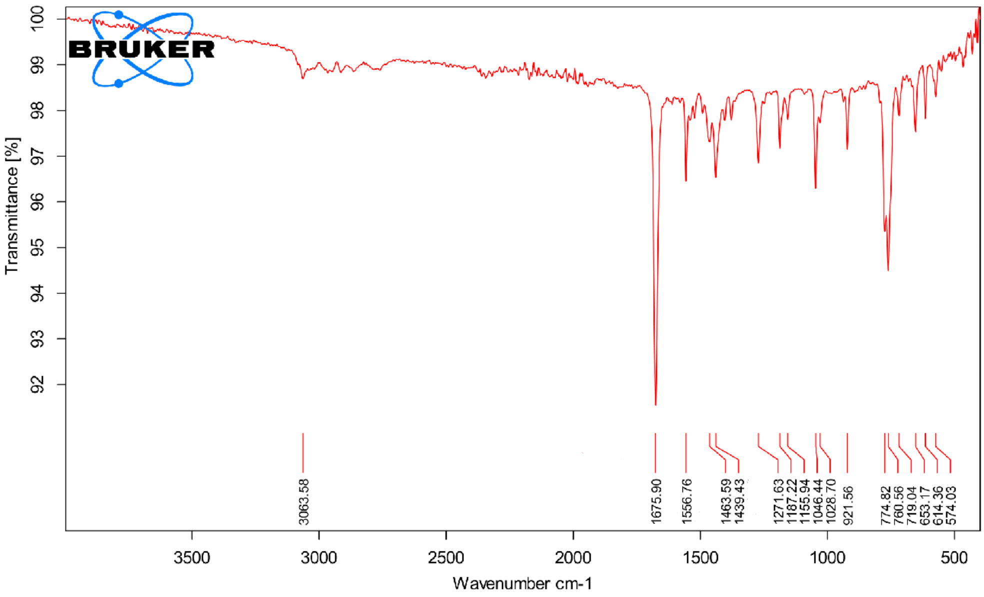

Supplementary Figure 2.9. FT-IR spectrum of **3**.

Synthesis and characterization of *meso*-H-dipyrromethane *meso*-H-DPM

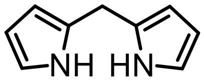

***meso*-H-DPM** was prepared using a method reported by Laha and co-workers [17], in which *p*-formaldehyde (3 g, 100 mmol, 1 eq.) was reacted in neat pyrrole (700 mL, 10 mol, 100 eq.) with $InCl_3$ (2.2 g, 10 mmol. 0.1 eq.) at 55 °C for 2 hours under inert atmosphere. After quenching the acid catalyst by stirring the mixture with NaOH (12 g, 300 mmol, 3 eq.) for 1 hour at room temperature, the crude was filtered and the excess of pyrrole in the filtrate removed by vacuum distillation maintaining the temperature of the rotary evaporator bath below 35 ºC. The resulting mixture was then subjected to column chromatography using *n*-heptane/DCM/AcOEt (7:2:1) as eluent, affording ***meso*-H-DPM** as a white solid (11.25 g, 77 %).

^{1}H- and ^{13}C-NMR data were consistent with those reported in the literature [17].

Synthesis and characterization of 5,15-bis(10-(2,6-dimethylphenyl)anthracen-9-yl)porphyrin H_2Por(dmpa)$_2$

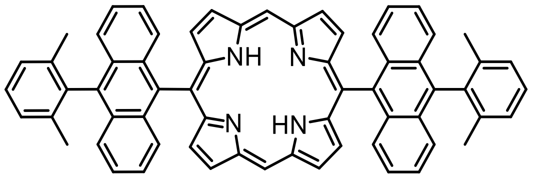

H_2Por(dmpa)$_2$ was synthesized using standard Lindsey conditions [18].

***meso*-H-DPM** (146 mg, 1 mmol) and **3** (310 mg, 1 mmol, 1 eq.) were loaded in a flame-dried 250 mL round-bottom flask. Then, $CHCl_3$ (100 mL) and dry EtOH (0.75 mL) were added and the mixture was vigorously degassed with argon for 40 minutes under stirring and in the dark. Then, under argon, $BF_3(OEt_2)$ (0.04 mL) was added, whereupon the mixture turned dark red. The mixture was stirred in the dark for 45 minutes at room temperature, then DDQ (363 mg, 1.6 mmol, 1.6 eq.) was added in one batch and the mixture was heated to reflux for 1 hour. After that, the mixture was allowed to cool to room temperature and triethylamine (0.8 mL) was added. After stirring for 5 minutes, the solvents were evaporated under reduced pressure. The crude product was then purified by column chromatography using DCM/*n*-heptane (2:1) as eluent, where the first red-colored fraction (R_f = 0.70) was isolated. The solvents were evaporated under reduced pressure and the crude product was further purified by size exclusion chromatography (BioBeads, THF). The solvent was removed under reduced pressure and the

resulting crude product was suspended in MeOH, sonicated, filtered, washed with MeOH (10 mL), collected and dried under vacuum to yield **H_2Por(dmpa)$_2$** as a dark-violet solid (35 mg, 8 %).

^{1}H-NMR (400 MHz, $CDCl_3$): δ = 10.30 (s, 2H), 9.28 (d, *J* = 4.5 Hz, 4H), 8.57 (d, *J* = 4.5 Hz, 4H), 7.79 (d, *J* = 8.9 Hz, 4H), 7.53 – 7.50 (m, 2H), 7.47 (d, *J* = 7.2 Hz, 4H), 7.35 (dd, *J* = 8.4, 6.8 Hz, 4H), 7.17 (d, *J* = 8.9, 4H), 7.03 – 6.98 (m, 4H), 2.18 (s, 12H), –2.58 (s, 2H); **^{13}C-NMR** (100 MHz, $CDCl_3$): δ = 148.6, 145.8, 138.1, 135.4, 135.1, 134.91, 132.3, 131.4, 129.4, 129.2, 129.1, 128.1, 127.9, 126.1, 125.8, 125.5, 120.7, 105.5, 20.6; **MALDI-TOF MS** (DCTB matrix): *m/z* (% intensity) = 870.4 – 874.4 m/z $[M]^+$ (100 %); **UV/vis** ($CHCl_3$): λ_{max} (log ε) = 359 (4.38), 416 (5.27), 505 (4.19), 539 (3.66), 577 (3.73); **FT-IR (ATR)**: ν (cm^{-1}) = 2956, 2922, 2853, 1461, 1437, 1407, 1375, 1261, 1239, 1057, 1045, 1023, 967, 944, 907, 857, 822, 789, 768, 754, 732, 657.

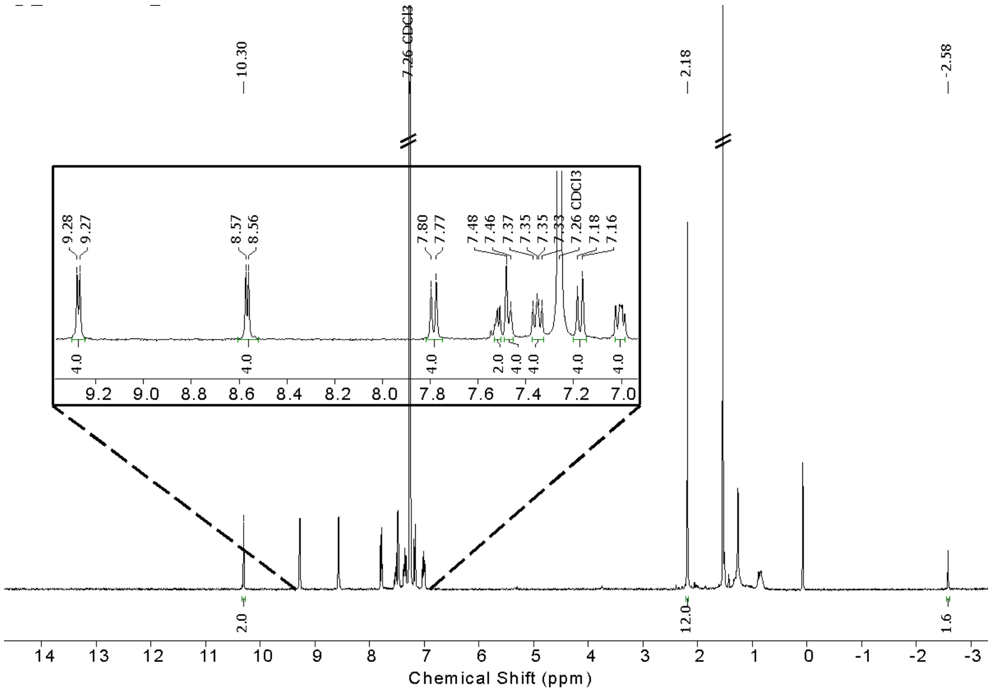

Supplementary Figure 2.10. ^{1}H-NMR spectrum of **H_2Por(dmpa)$_2$** in $CDCl_3$ (≠ = residual solvent signals).

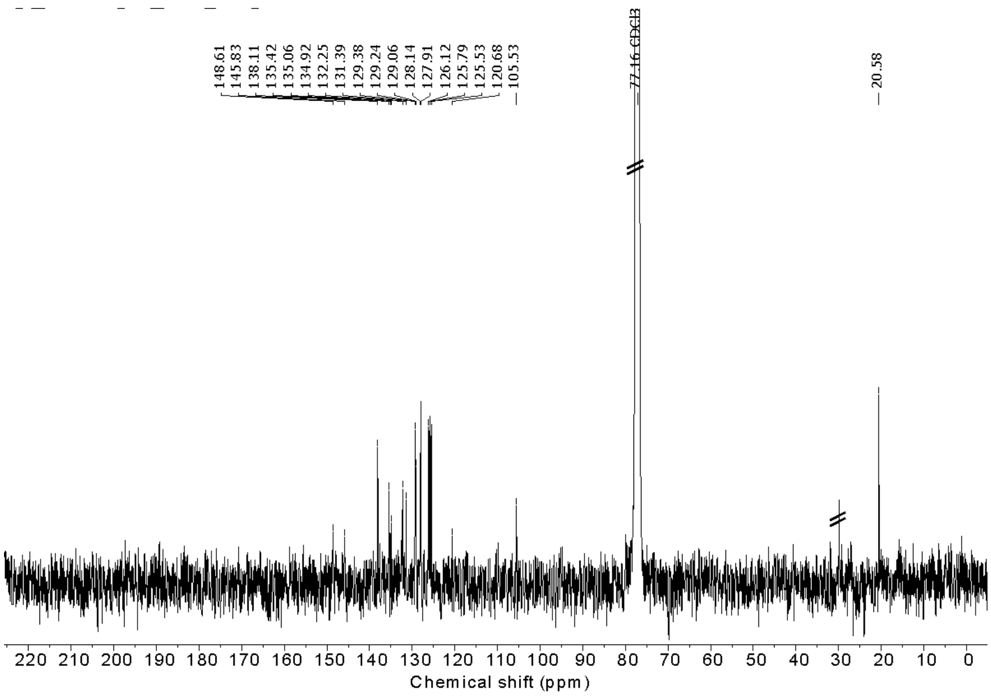

Supplementary Figure 2.11. ^{13}C-NMR spectrum of **H_2Por(dmpa)$_2$** in $CDCl_3$ (≠ = residual solvent signals). A better resolution could not be obtained due to solubility issues.

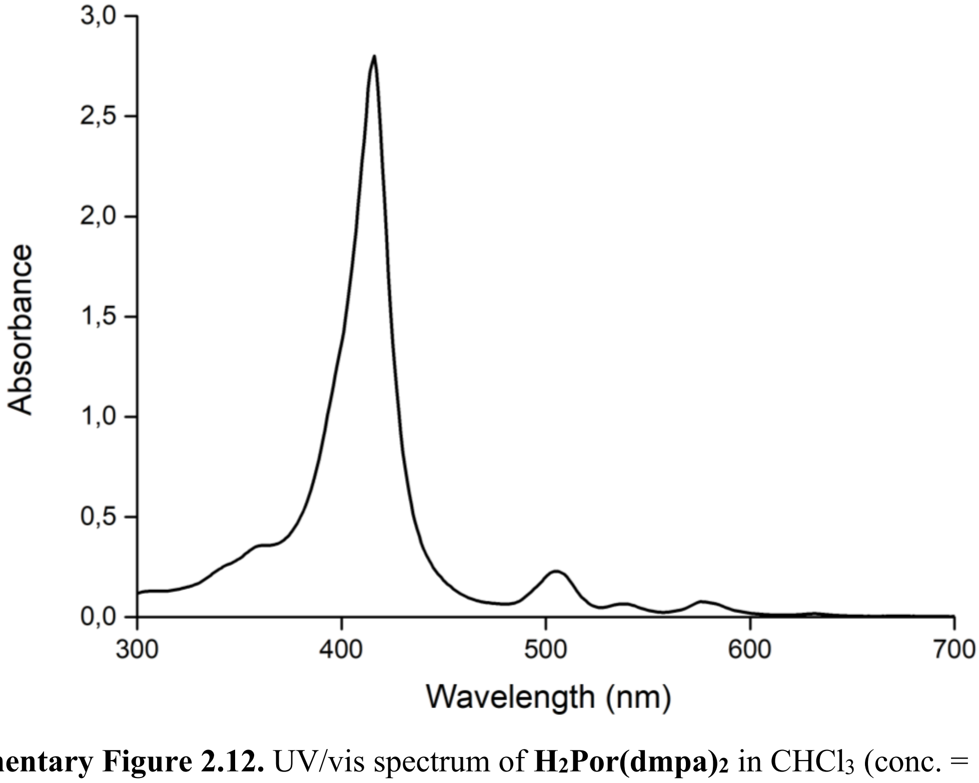

Supplementary Figure 2.12. UV/vis spectrum of **H_2Por(dmpa)$_2$** in $CHCl_3$ (conc. = 15 µM).

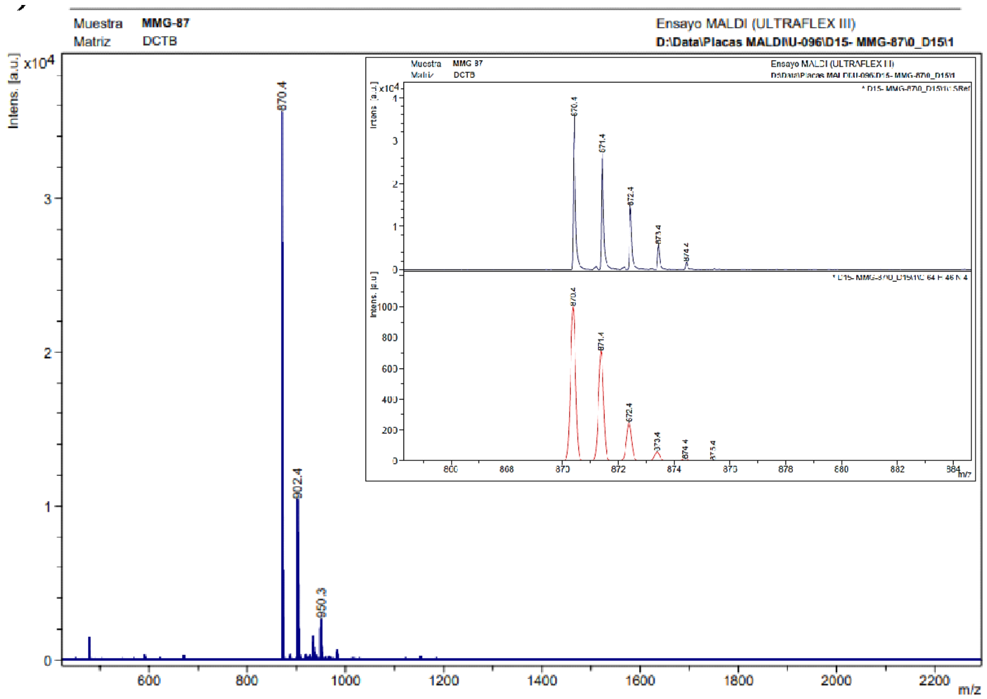

Supplementary Figure 2.13. MALDI-TOF mass spectrum of **H_2Por(dmpa)$_2$**. Inset: (a) Experimental isotopic resolution of the MALDI-TOF main peak at 870.4 m/z. (b) Calculated isotopic pattern for **H_2Por(dmpa)$_2$**.

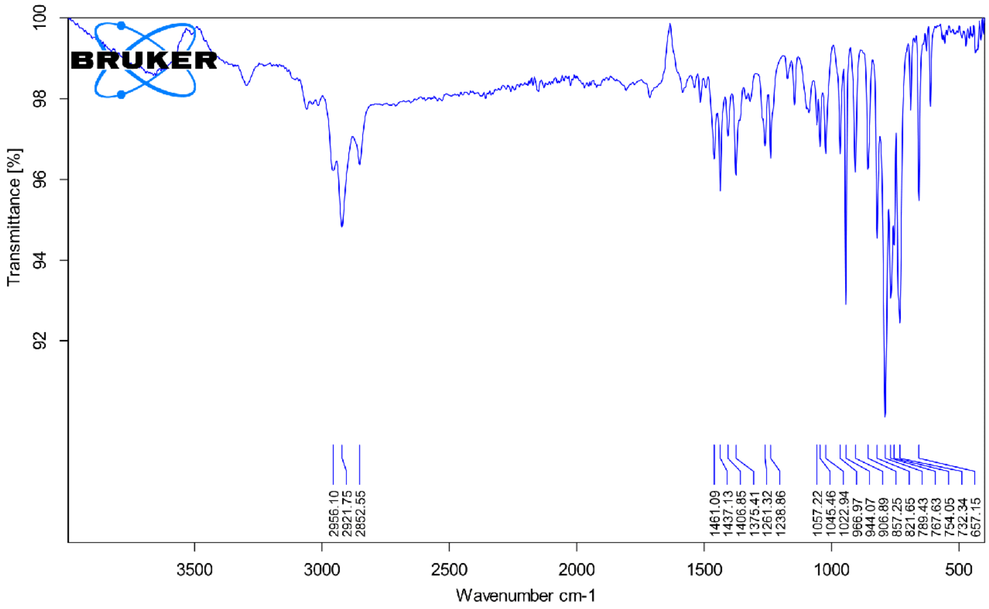

Supplementary Figure 2.14. FT-IR spectrum of **H_2Por(dmpa)$_2$**.

Synthesis and characterization of 5,15-bis(10-(2,6-dimethylphenyl)anthracen-9-yl)-Zn(II) porphyrin ZnPor(dmpa)$_2$

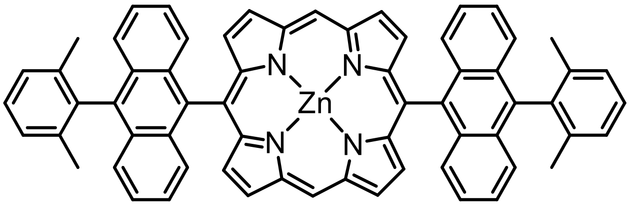

H$_2$Por(dmpa)$_2$ (20 mg, 0.023 mmol) and Zn(OAc)$_2$ (42 mg, 0.23 mmol, 10 eq.) were loaded in a flame-dried 25 mL Schlenk tube and three cycles of vacuum/argon backfilling were applied. Then, dry THF (8 mL) was added, and the mixture was heated to reflux and stirred under argon for 48 hours. The mixture was then allowed to cool to room temperature, quenched with water (20 mL) and extracted with DCM (3 × 25 mL). The combined organic layers were washed with water (40 mL) and brine (40 mL), dried over anhydrous MgSO$_4$, filtered and dried under reduced pressure using a rotary evaporator with a temperature bath below 30 ºC. The crude product was subjected to column chromatography using DCM/*n*-heptane (2:1) as eluent, where the second (if there's starting material left) red-colored fraction (R_f = 0.64) was isolated. Solvents were removed *in vacuo* maintaining the bath temperature below 30 ºC, and the product was further purified by size exclusion chromatography (BioBeads, THF). The solvent was removed under reduced pressure maintaining the bath temperature below 30 ºC and the resulting solid was suspended in pentane, sonicated, filtered, washed with pentane (5 × 2 mL), collected and dried under vacuum to yield **ZnPor(dmpa)$_2$** as a red powder (16.8 mg, 78 %).

^{1}H-NMR (400 MHz, CDCl$_3$): δ = 10.32 (s, 2H), 9.33 (d, *J* = 4.5 Hz, 4H), 8.64 (d, *J* = 4.5 Hz, 4H), 7.79 (d, *J* = 8.9 Hz, 4H), 7.55 – 7.50 (m, 2H), 7.48 (d, *J* = 7.3 Hz, 4H), 7.37 – 7.31 (m, 4H), 7.12 (d, *J* = 8.9 Hz, 4H), 7.01 – 6.95 (m, 4H), 2.20 (s, 12H); **^{13}C-NMR** (100 MHz, CDCl$_3$): δ = 168.3, 151.7, 149.9, 138.2, 135.1, 132.7, 132.6, 129.4, 129.0, 127.9, 126.1, 125.6, 125.4, 114.4, 105.6, 103.2, 20.6; **MALDI-TOF MS** (DCTB matrix): *m/z* (% intensity) = 932.3 – 939.3 m/z $[M]^+$ (100 %); **UV/vis** (CHCl$_3$): λ_{max} (log ε) = 419 (5.33), 542 (4.23); **FT-IR (ATR)**: ν (cm^{-1}) = 3649, 2954, 2922, 2854, 1460, 1438, 1364, 1313, 1247, 1230, 1213, 1156, 1120, 1059, 1024, 991, 965, 860, 834, 787, 761, 739, 729, 658.

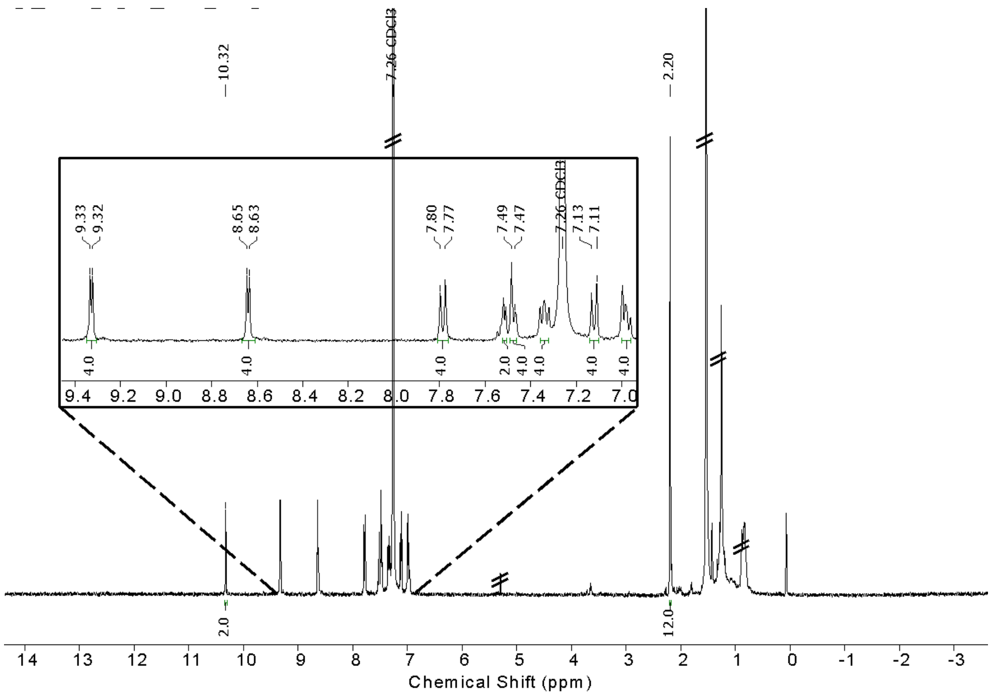

Supplementary Figure 2.15. ^{1}H-NMR spectrum of **ZnPor(dmpa)$_2$** in $CDCl_3$ (≠ = residual solvent signals).

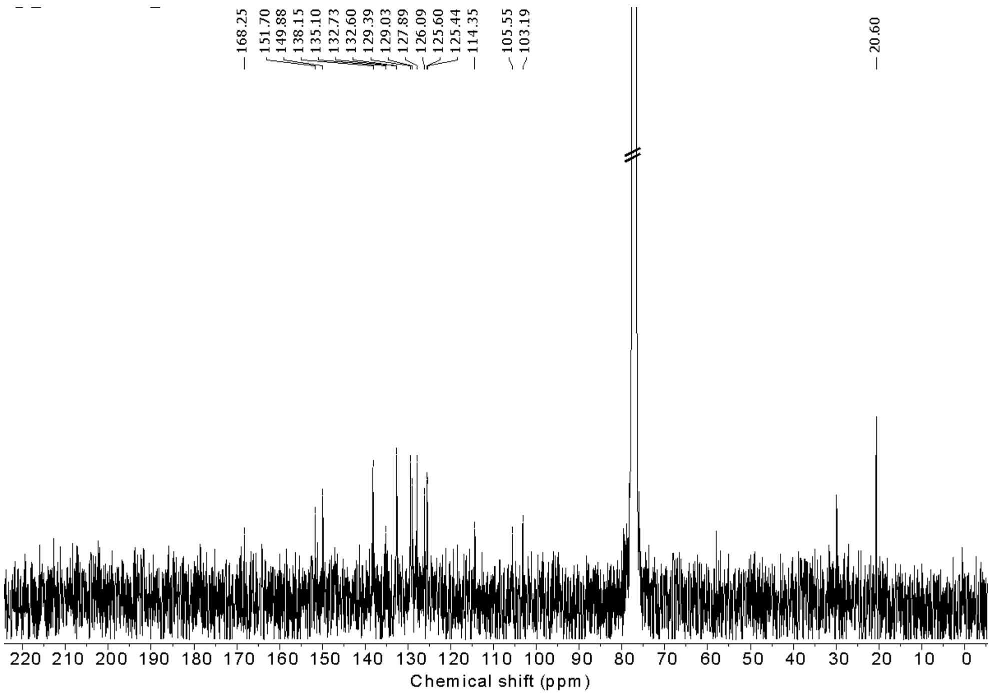

Supplementary Figure 2.16. ^{13}C-NMR spectrum of **ZnPor(dmpa)$_2$** in $CDCl_3$ (≠ = residual solvent signals). A better resolution could not be obtained due to solubility issues.

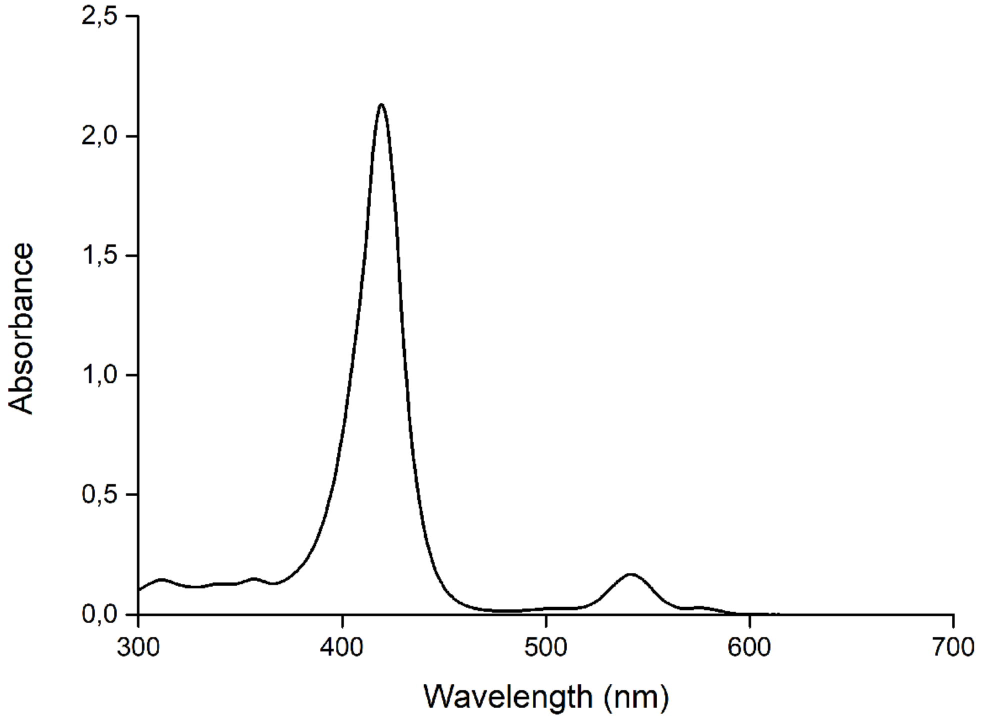

Supplementary Figure 2.17. UV/vis spectrum of **ZnPor(dmpa)$_2$** in $CHCl_3$ (conc. = 10 μM).

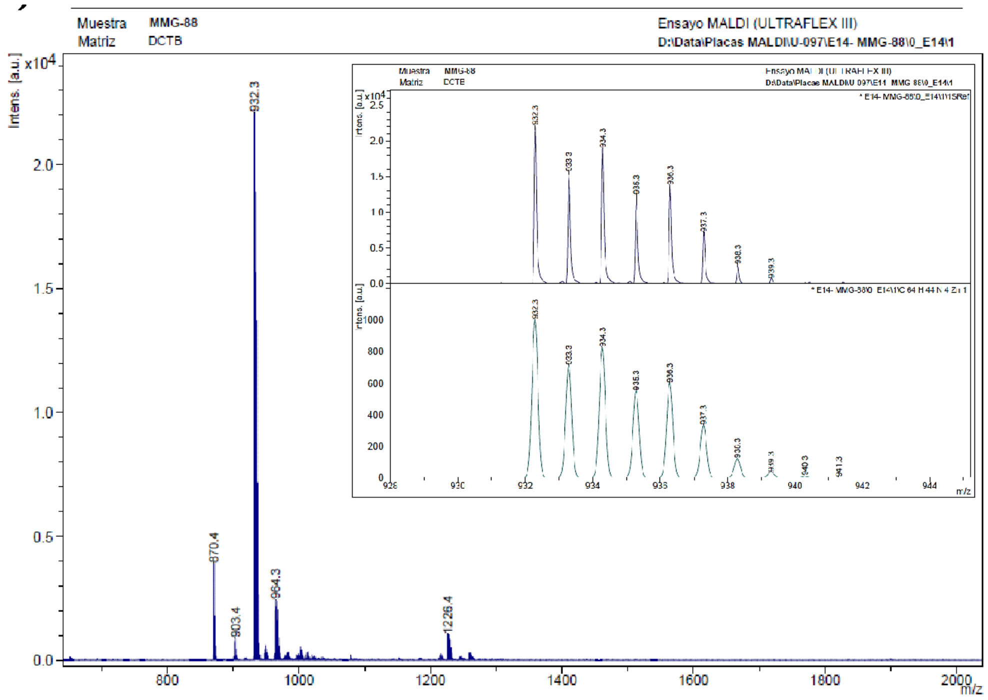

Supplementary Figure 2.18. MALDI-TOF mass spectrum of **ZnPor(dmpa)$_2$**. Inset: (a) Experimental isotopic resolution of the MALDI-TOF main peak at 932.3 m/z. (b) Calculated isotopic pattern for **ZnPor(dmpa)$_2$**.

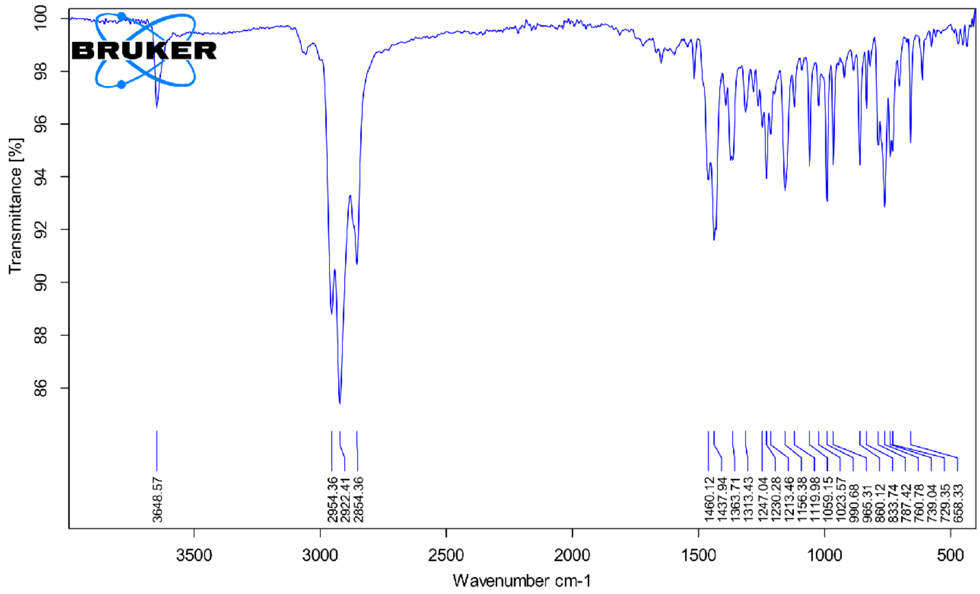

Supplementary Figure 2.19. FT-IR spectrum of **ZnPor(dmpa)$_2$**.

Synthesis and characterization of 5,10,15,20-tetrakis(10-(2,6-dimethylphenyl)anthracen-9-yl)porphyrin H$_2$Por(dmpa)$_4$

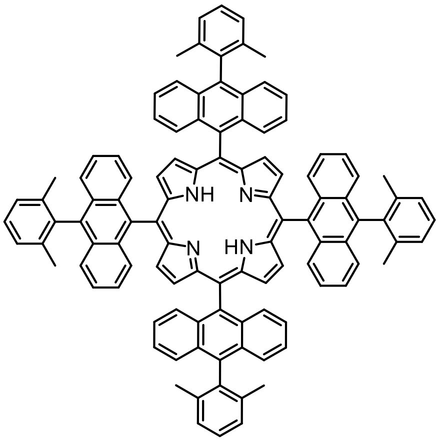

H$_2$Por(dmpa)$_4$ was synthesized using kinetically controlled Lindsey conditions [19]. Pyrrole (67 mg, 0.07 mL, 1 mmol) and **3** (310 mg, 1 mmol, 1 eq.) were loaded in a flame-dried 250 mL round-bottom flask. Then, $CHCl_3$ (100 mL) and dry EtOH (0.75 mL) were added, and the mixture was vigorously degassed with argon for 40 minutes under stirring and in the dark. Then, under argon, $BF_3(OEt_2)$ (0.04 mL) was added, whereupon the mixture turned green and then yellow. After stirring the mixture in the dark for 1 minute at room temperature, DDQ (363 mg, 1.6 mmol, 1.6 eq.) was added in one batch and the mixture was heated to reflux for 1 hour. Then, the mixture was cooled to room temperature, triethylamine (0.8 mL) was added and, after 5 minutes stirring, the solvents were evaporated under reduced pressure. The crude product was then purified by column chromatography using DCM/*n*-heptane (3:2) as eluent, where the red-

colored fraction (R_f = 0.69) was isolated. Then, the solvents were evaporated, and the obtained crude product was purified by size exclusion chromatography (BioBeads, THF). The solvent was evaporated under reduced pressure and the resulting crude product was suspended in MeOH, sonicated, filtered, washed with MeOH (10 mL), collected and dried under vacuum to yield **H_2Por(dmpa)$_4$** as a dark-violet solid (12.2 mg, 3.4 %).

^{1}H-NMR (400 MHz, $CDCl_3$): δ = 8.26 (s, 8H), 7.67 (d, J = 9.0 Hz, 8H), 7.44 (d, J = 6.8 Hz, 4H), 7.37 (d, J = 7.6 Hz, 12H), 7.31 (d, J = 9.0 Hz, 8H), 7.06 – 7.01 (m, 12H), 2.04 (s, 24H), –1.55 (s, 2H); **^{13}C-NMR** was unsuccessful due to solubility issues; **MALDI-TOF MS** (DCTB matrix): m/z (% intensity) = 1430.5 – 1435.6 m/z $[M]^+$ (100 %); **UV/vis** ($CHCl_3$): λ_{max} (log ε) = 359 (4.48), 380 (4.54), 427 (5.26), 521 (4.24), 553 (3.87), 594 (3.87), 653 (3.69); **FT-IR (ATR)**: ν (cm^{-1}) = 3358, 2922, 2852, 1660, 1461, 1410, 1377, 1259, 1215, 1089, 1023, 979, 942, 800, 756, 665, 611.

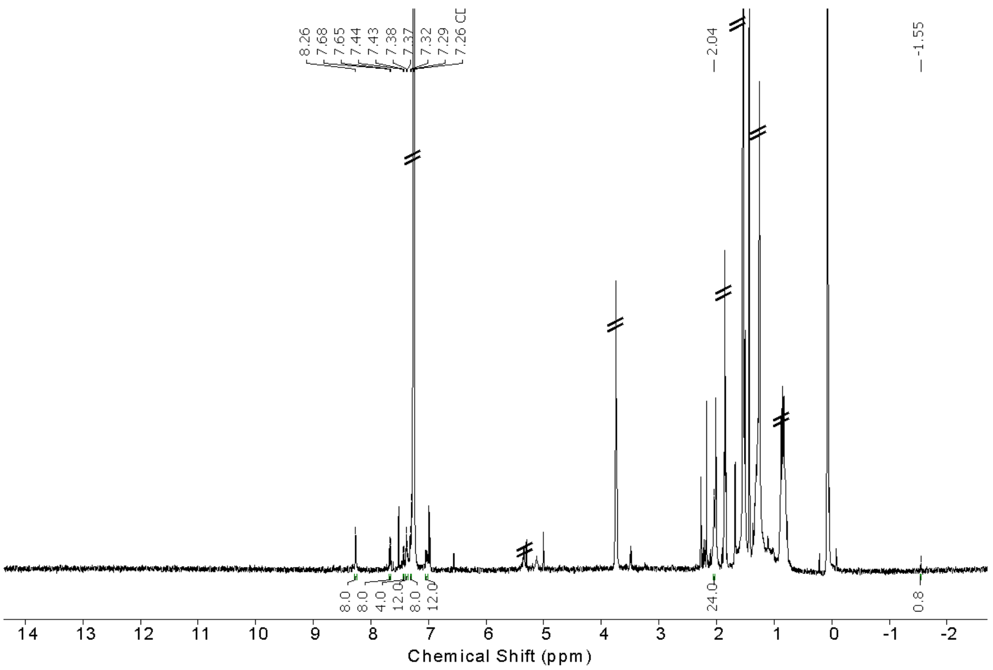

Supplementary Figure 2.20. ^{1}H-NMR spectrum of **H_2Por(dmpa)$_4$** in $CDCl_3$ (≠ = residual solvent signals). A better result could not be obtained due to solubility issues.

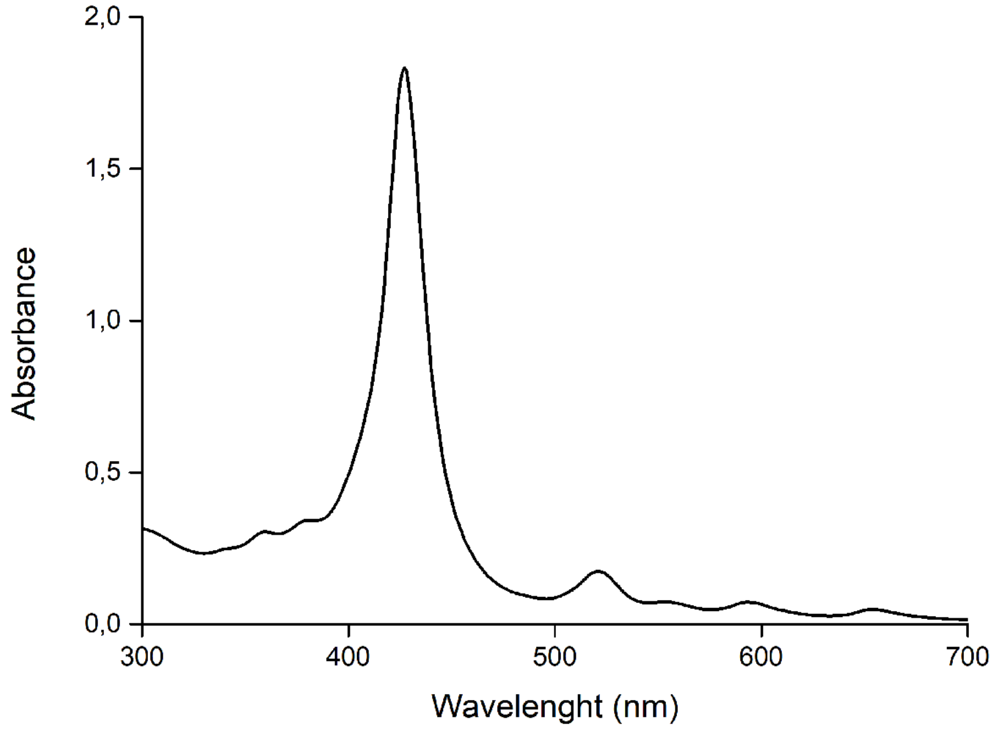

Supplementary Figure 2.21. UV/vis spectrum of **H_2Por(dmpa)$_4$** in $CHCl_3$ (conc. = 10 µM).

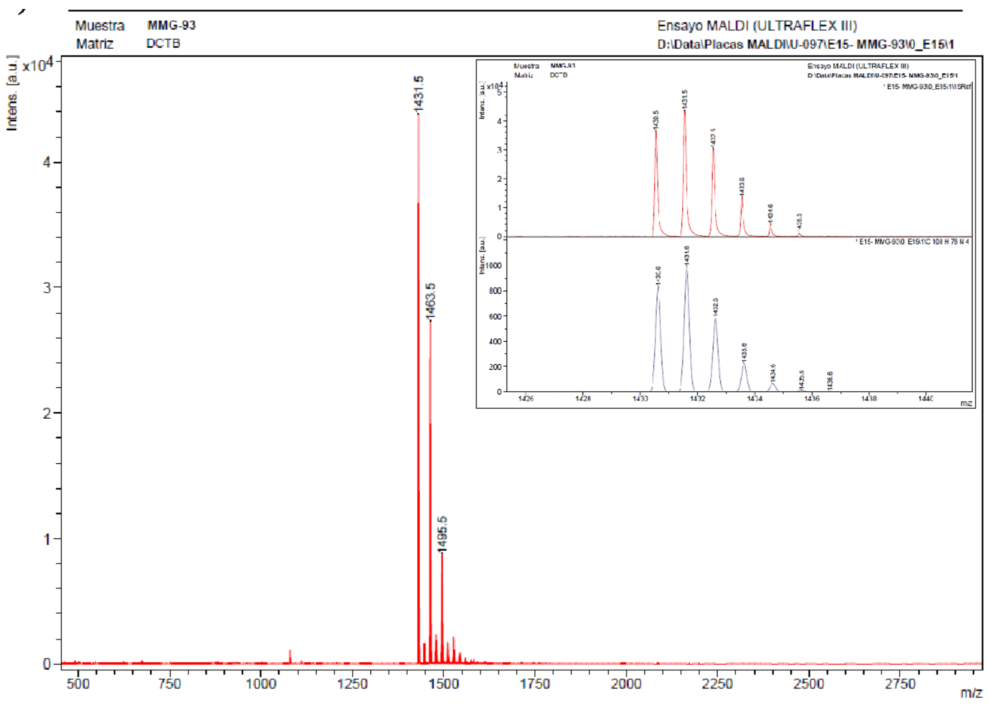

Supplementary Figure 2.22. MALDI-TOF mass spectrum of **H_2Por(dmpa)$_4$**. Inset: (a) Experimental isotopic resolution of the MALDI-TOF main peak at 1431.5 m/z. (b) Calculated isotopic pattern for **H_2Por(dmpa)$_4$**.

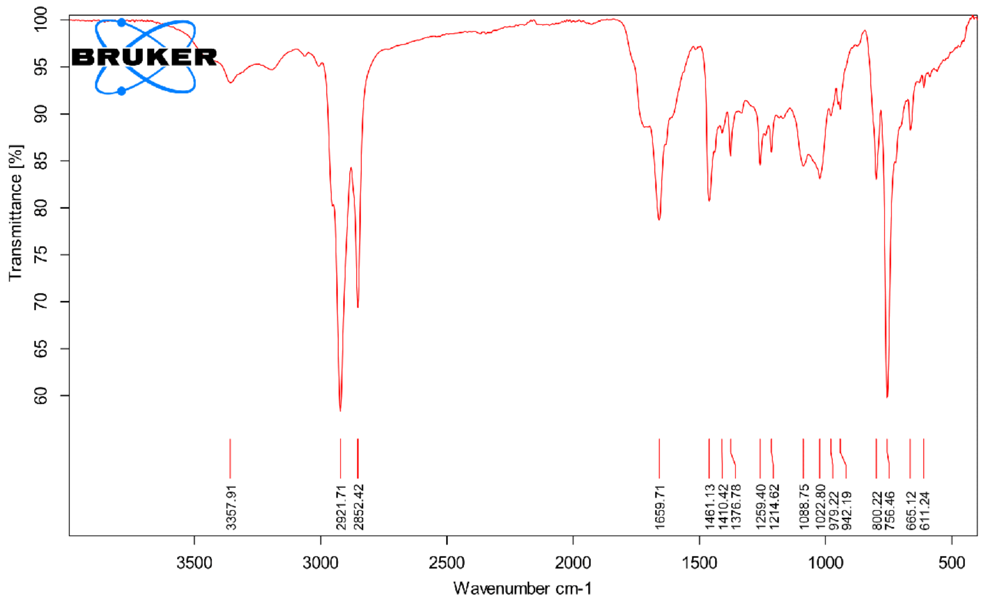

Supplementary Figure 2.23. FT-IR spectrum of **H_2Por(dmpa)$_4$**.

Synthesis and characterization of 5,10,15,20-tetrakis(10-(2,6-dimethylphenyl)anthracen-9-yl)-Zn(II)porphyrin ZnPor(dmpa)$_4$

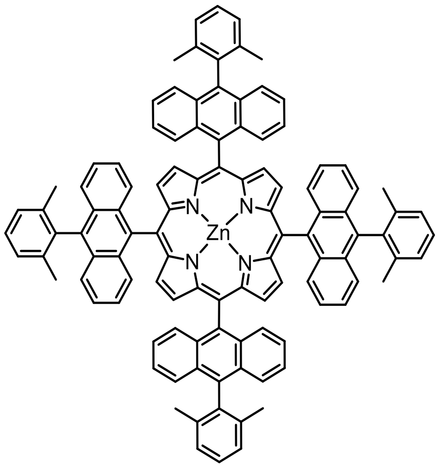

H_2Por(dmpa)$_4$ (5 mg, 0.0035 mmol) and Zn(OAc)$_2$ (6.4 mg, 0.035 mmol, 10 eq.) were loaded in a flame-dried 10 mL Schlenk tube and three cycles of vacuum/argon backfilling were applied. Then, dry THF (3 mL) was added, and the mixture was heated to reflux and stirred under argon for 24 hours. The reaction was cooled to room temperature, water (10 mL) was added, and the mixture was extracted with DCM (3 × 15 mL). The combined organic layers were washed with water (20 mL) and brine (20 mL), dried over anhydrous $MgSO_4$, filtered and dried under reduced pressure. The resulting crude product was subjected to column chromatography using DCM/*n*-heptane (3:2) as eluent, where the red-colored fraction (R_f = 0.62) was collected. Solvents were evaporated under reduced pressure, and the product was further purified by size exclusion chromatography (BioBeads, THF). The solution was dried under reduced pressure

and the resulting product was suspended in MeOH, sonicated, filtered, washed with more MeOH (3 × 2 mL), collected and dried under vacuum to yield **ZnPor(dmpa)₄** as a red solid (4 mg, 77 %).

^{1}H-NMR (400 MHz, THF-d_8): δ = 8.18 (s, 8H), 7.66 (d, J = 8.9 Hz, 8H), 7.46 – 7.36 (m, 12H), 7.33 – 7.23 (m, 16H), 7.02 – 6.96 (m, 8H), 2.02 (s, 24H); **^{13}C-NMR** (100 MHz, THF-d_8): δ = 152.6, 139.2, 138.6, 138.0, 137.6, 136.4, 133.1, 130.3, 129.8, 129.0, 128.7, 126.5, 126.2, 117.5, 30.8; **MALDI-TOF MS** (DCTB matrix): m/z (% intensity) = 1492.5 – 1500.5 m/z $[M]^+$ (100%); **UV/vis** ($CHCl_3$): λ_{max} (log ε) = 359 (4.34), 380 (4.33), 436 (5.32), 559 (4.26); **FT-IR (ATR)**: ν (cm^{-1}) = 2953, 2921, 2852, 1659, 1460, 1377, 1260, 1090, 1062, 1020, 964, 795, 760, 719.

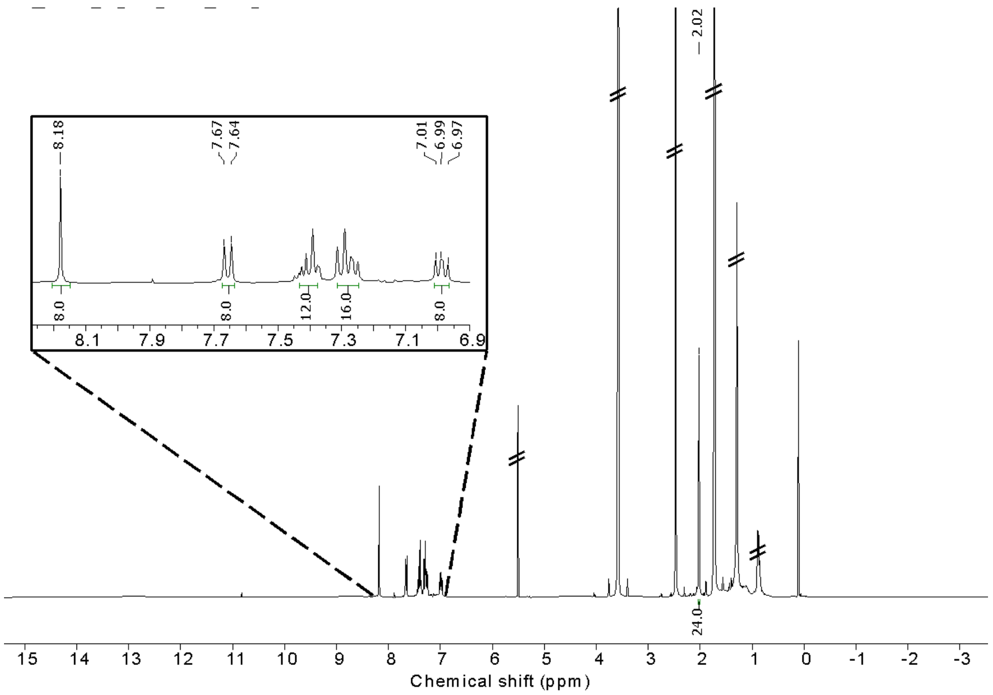

Supplementary Figure 2.24. ^{1}H-NMR spectrum of **ZnPor(dmpa)₄** in THF-d_8 (≠ = residual solvent signals).

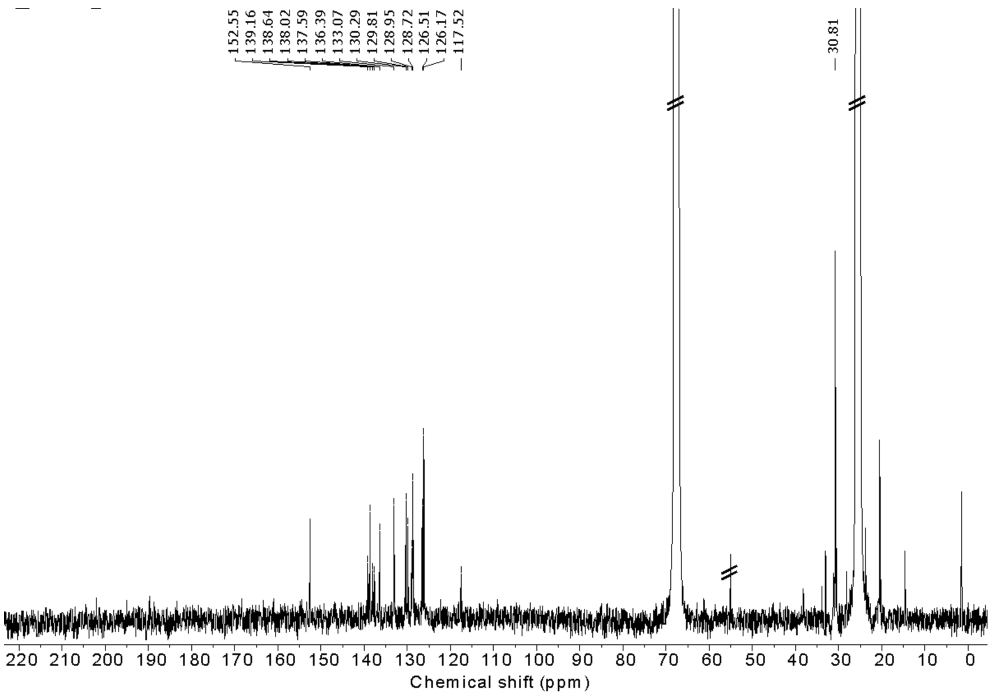

Supplementary Figure 2.25. ^{13}C-NMR spectrum of **ZnPor(dmpa)$_4$** in THF-d_8 (≠ = residual solvent signals).

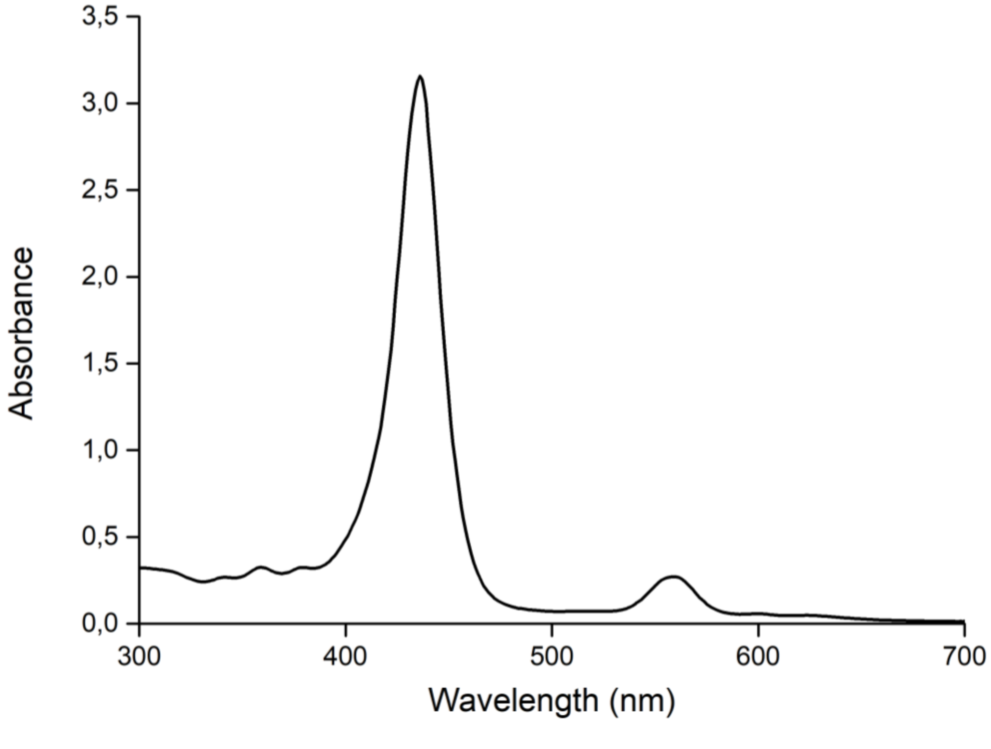

Supplementary Figure 2.26. UV/vis spectrum of **ZnPor(dmpa)$_4$** in $CHCl_3$ (conc. = 15 µM).

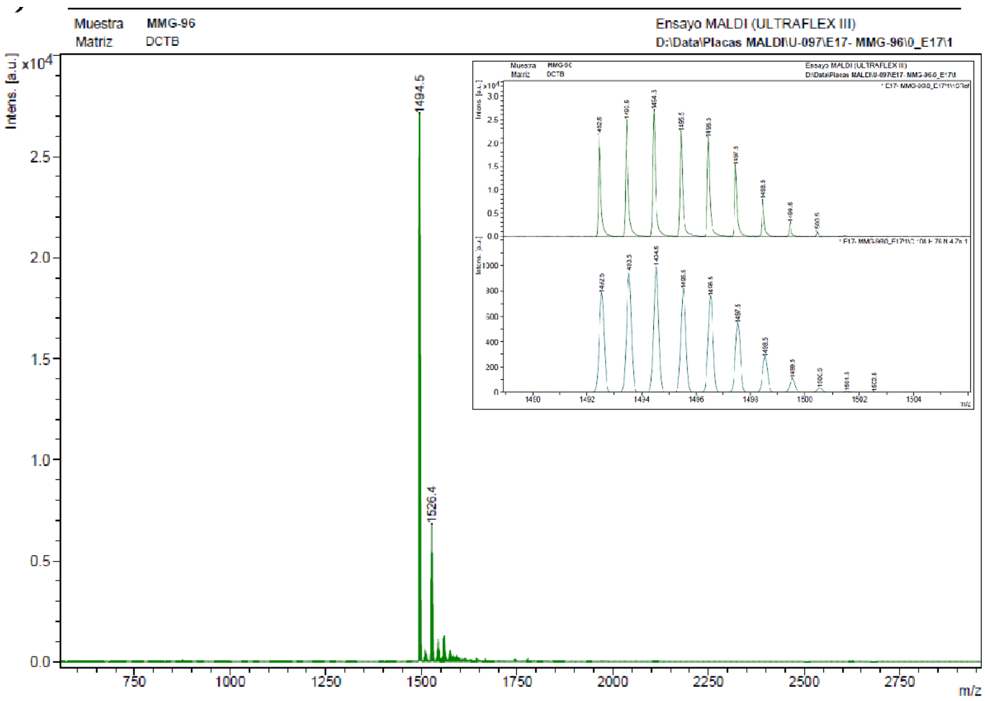

Supplementary Figure 2.27. MALDI-TOF mass spectrum of **ZnPor(dmpa)$_4$**. Inset: (a) Experimental isotopic resolution of the MALDI-TOF main peak at 1494.5 m/z. (b) Calculated isotopic pattern for **ZnPor(dmpa)$_4$**.

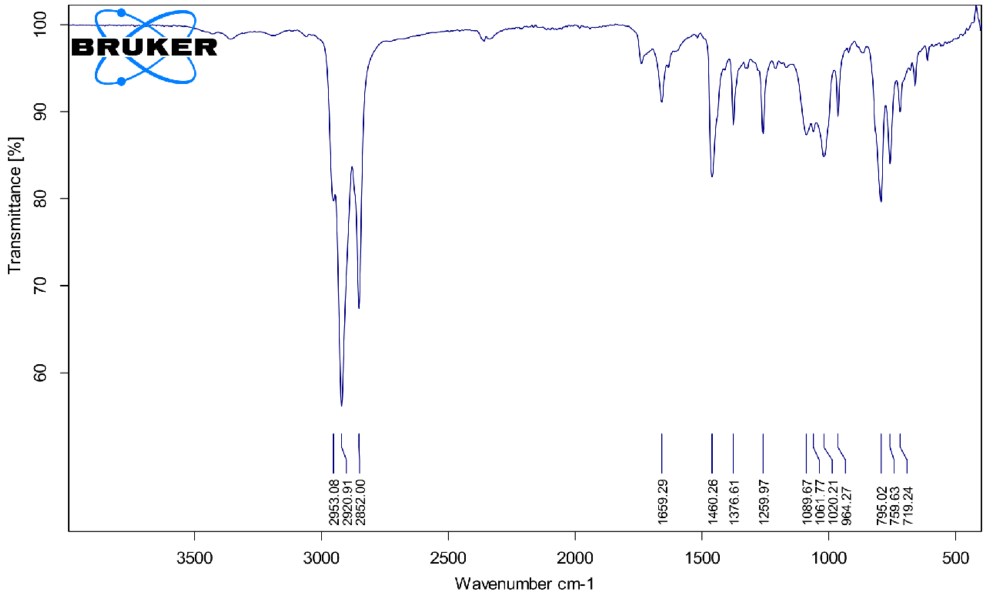

Supplementary Figure 2.28. FT-IR spectrum of **ZnPor(dmpa)$_4$**.

S3 Resonance structures of ZnPorT2 and ZnPorT4

The potential open-shell character of **ZnPorT2** and **ZnPorT4** was evaluated by considering their resonance structures.

Usually, in polyaromatic hydrocarbons (PAHs), the resonance structure that contributes the most to the resonance hybrid is the one which presents the largest number of disjoint aromatic sextets (i.e., isolated benzene rings) as stated by the Clar's sextet empirical rule. This rule, for example, can provide useful information when applied to nanographenes, often in combination with the Lieb's theorem which addresses the magnetic ground states of these systems.

Following this concept, in Por-NGs hybrid systems, besides counting the number of Clar's sextets, the aromatic stabilization energy (ASE) of the Por macrocycle needs to be considered as well. The ASE per Clar's sextet is approximately one third of the energy of a C-C π-bond [20,21], and the ASE of Por is less than the energy of a C-C π-bond [22]. Therefore, in the case of **ZnPorT2**, the formal "fusion" of two open-shell diradical triangulenes to the closed-shell structure of a porphine would give rise to several resonance structures, among which can be identified a structure featuring four unpaired electrons and exhibiting global aromaticity at the Por macrocycle, and another featuring two unpaired electrons and no global aromaticity at the macrocycle (Supplementary Figs. S3.1 and S3.2).

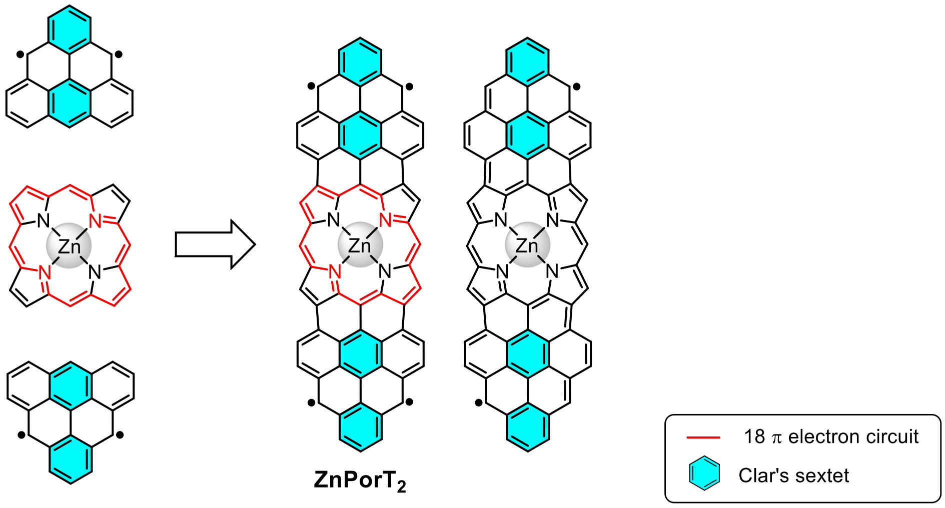

Supplementary Figure 3.1. Formal "fusion" of two open-shell diradical triangulenes to a porphine leading to **ZnPorT2**.

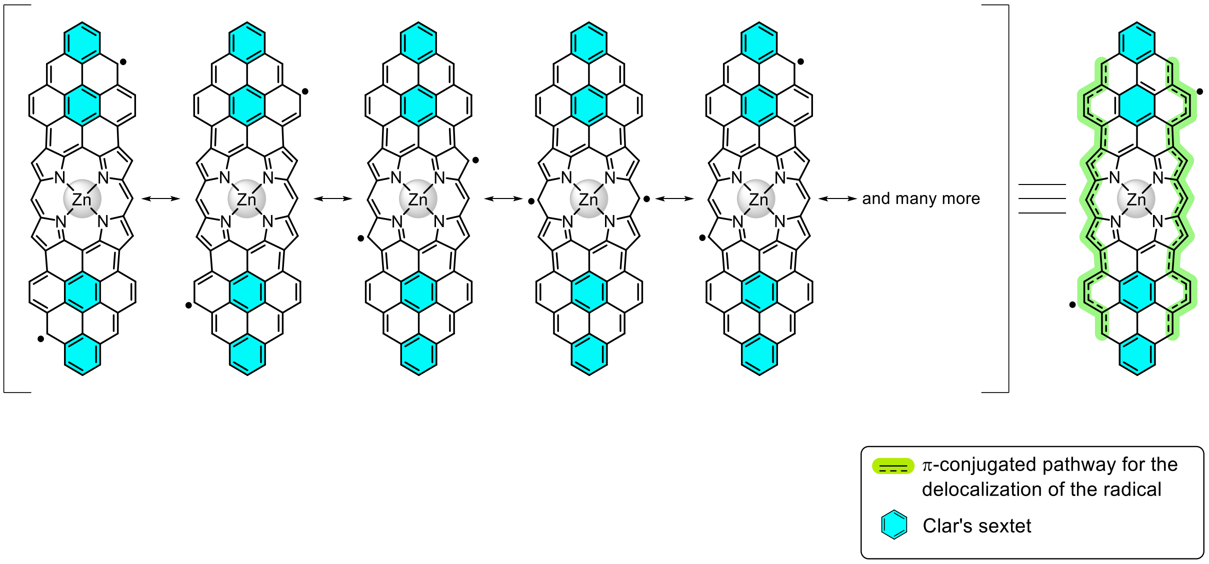

Supplementary Figure 3.2. Some possible diradical resonance structures of **ZnPorT2**.

Both resonance structures contain the same number of Clar's sextets (*i.e.*, six). Based on the above-mentioned comparison between the energy of a C-C π-bond and ASE of a Clar's sextet and Por macrocycle, **ZnPorT2** tends to "break" the aromaticity at the Por macrocycle in order to form a resonance structure with minimum number of unpaired electrons (Supplementary Fig. S3.2). In the electronic structure section in the main text, we show that this open-shell configuration is well-supported by the experiment and calculations. This counting rule also applies to our previously reported **ZnPorA2** system (Supplementary Fig. S3.3) [23].

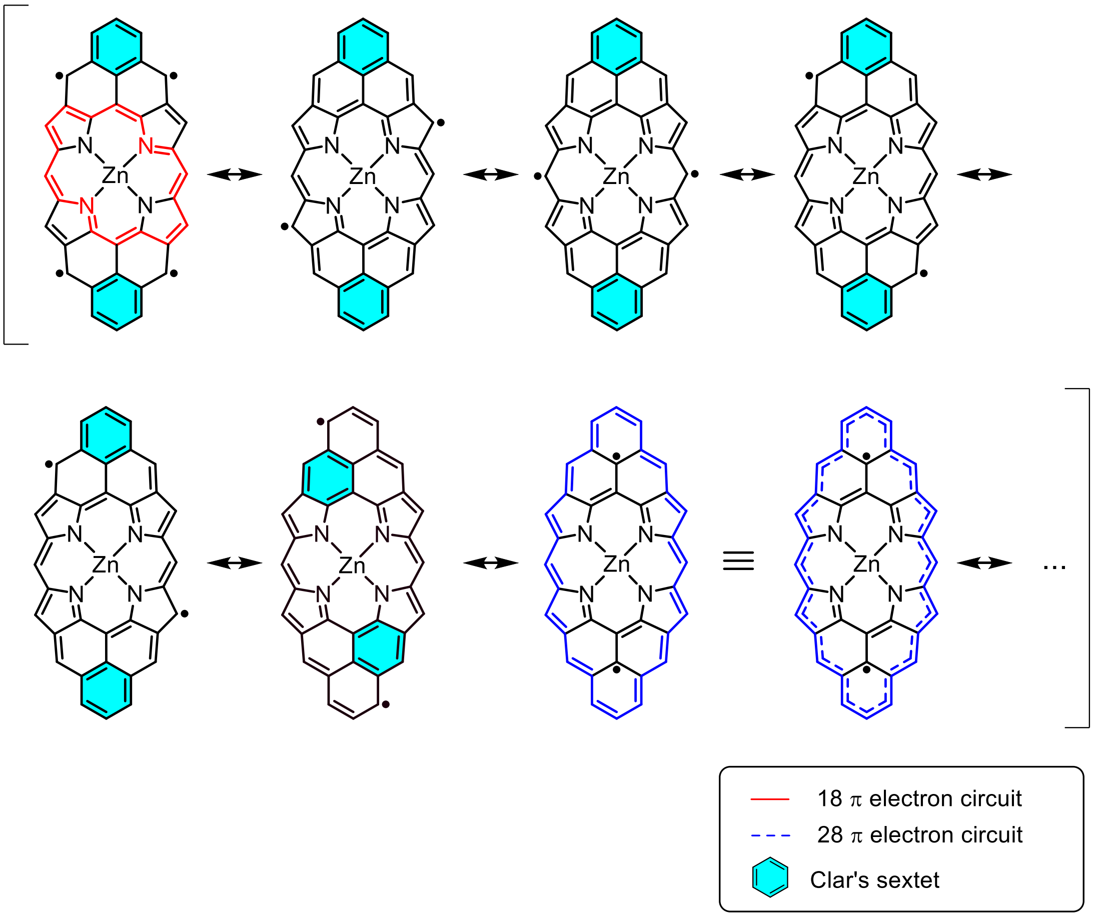

Supplementary Figure 3.3. Some possible resonance structures of **PorA2**.

Tuning to **ZnPorT4**, applying the same rule, several non-Kekulé diradical resonance structures having eight Clar's sextets can be drawn (Supplementary Fig. S3.4). In addition, a closed-shell Kekulé electronic configuration also featuring eight Clar's sextets and an 18 π-electron circuit on the central cavity can be drawn. In this sense, **ZnPorT4** is supposed to be in closed-shell configuration. However, in the later section, **ZnPorT4** is found to be in a diradical open-shell configuration.

It is worth noticing that in our previous study on **ZnPorA4** (Supplementary Fig. S3.5) [23], a closed-shell ground state configuration is predicted, whereas **ZnPorT4** possesses an antiferromagnetic ground state (details are provided in the main text). This indicates that solely quantifying the aromaticity within these four-fold Por-NGs is insufficient to determine their most contributed resonance structures and that additional factors must be considered. For **ZnPorA4** and **ZnPorT4**, the evolution of the ground state from closed-shell to open shell can be rationalized by the competition between hybridization energy and effective Coulomb repulsion [24,25]: As the molecular size increases, the HOMO–LUMO gap decreases, which

in turn reduces the effective Coulomb repulsion required to spatially separate the frontier electrons [26].

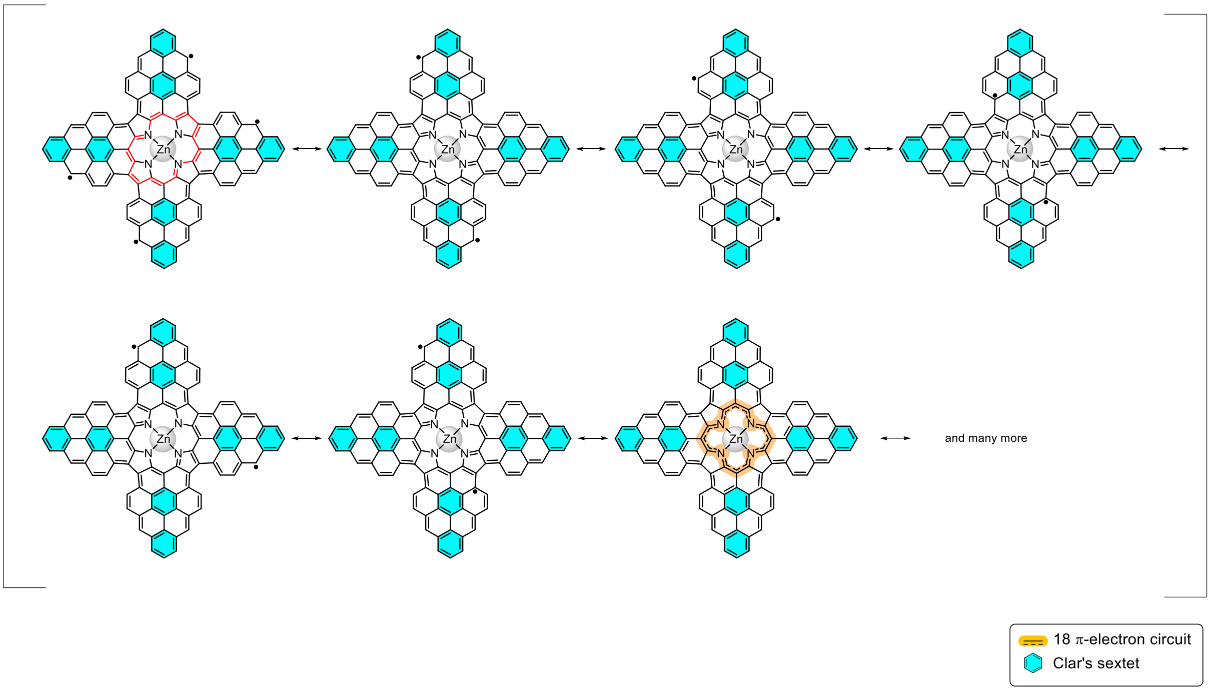

Supplementary Figure 3.4. Some possible resonance structures of **ZnPorT4**.

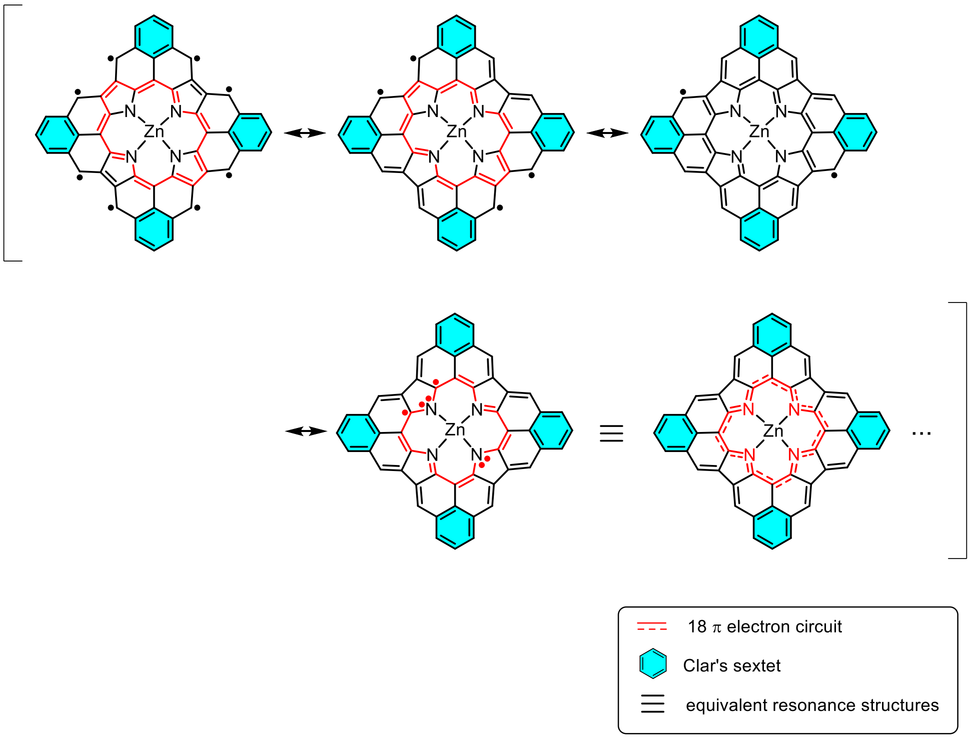

Supplementary Figure 3.5. Some possible resonance structures of **PorA4**.

S4 Supplementary STM measurements and DFT calculations

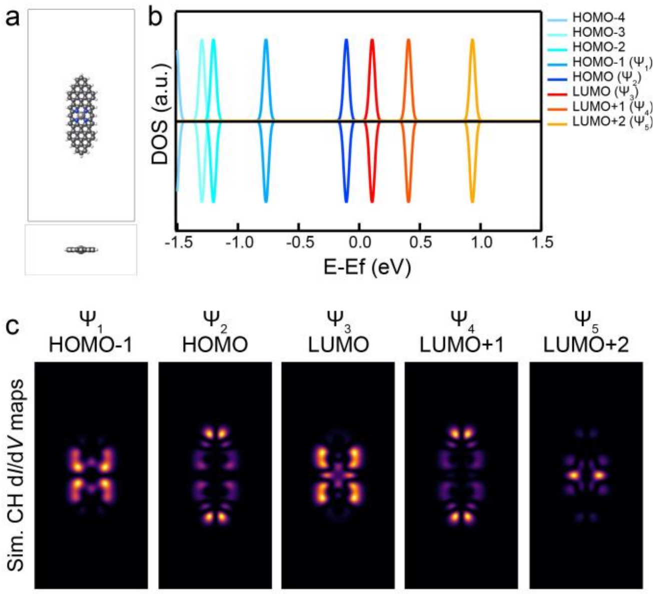

Supplementary Figure 4.1. DFT calculations of **ZnPorT2** in gas phase at the PBE level. (a) Geometry optimized molecular model of **ZnPorT2**. (b) Calculated density of states (DOS) in unrestricted Kohn-Sham conditions. Broadening: 0.05eV (c) Simulated dI/dV maps at HOMO-1 (Ψ_1), HOMO (Ψ_2), LUMO (Ψ_3), LUMO+1 (Ψ_4) and LUMO+2 (Ψ_5), evaluated in a plane 5 Å above the ribbon, FWHM = 0.04 eV. Dark grey, blue, white and grey balls represent carbon, nitrogen, hydrogen and zinc, respectively.

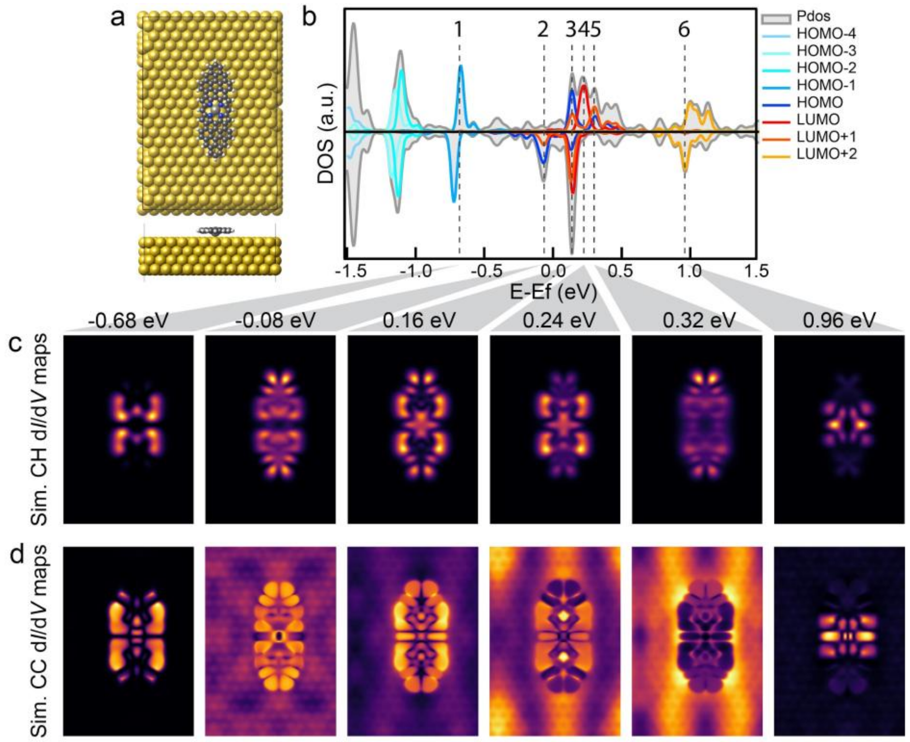

Supplementary Figure 4.2. DFT calculations of **ZnPorT$_2$** adsorbed on Au(111) surface at PBE level. (a) Geometry optimized molecular model of **ZnPorT$_2$** on Au(111) surface. (b) Density of states in unrestricted Kohn-Sham conditions. Broadening: 0.05eV (c) Simulated d*I*/d*V* maps using energies marked in (b) in constant height mode, evaluated in a plane 5 Å above the ribbon, FWHM = 0.08 eV. (d) Simulated d*I*/d*V* maps using energies marked in (b) in constant current mode. FWHM broadening: 0.08 eV. Dark grey, blue, white, grey and yellow balls represent carbon, nitrogen, hydrogen, zinc, and gold atoms, respectively.

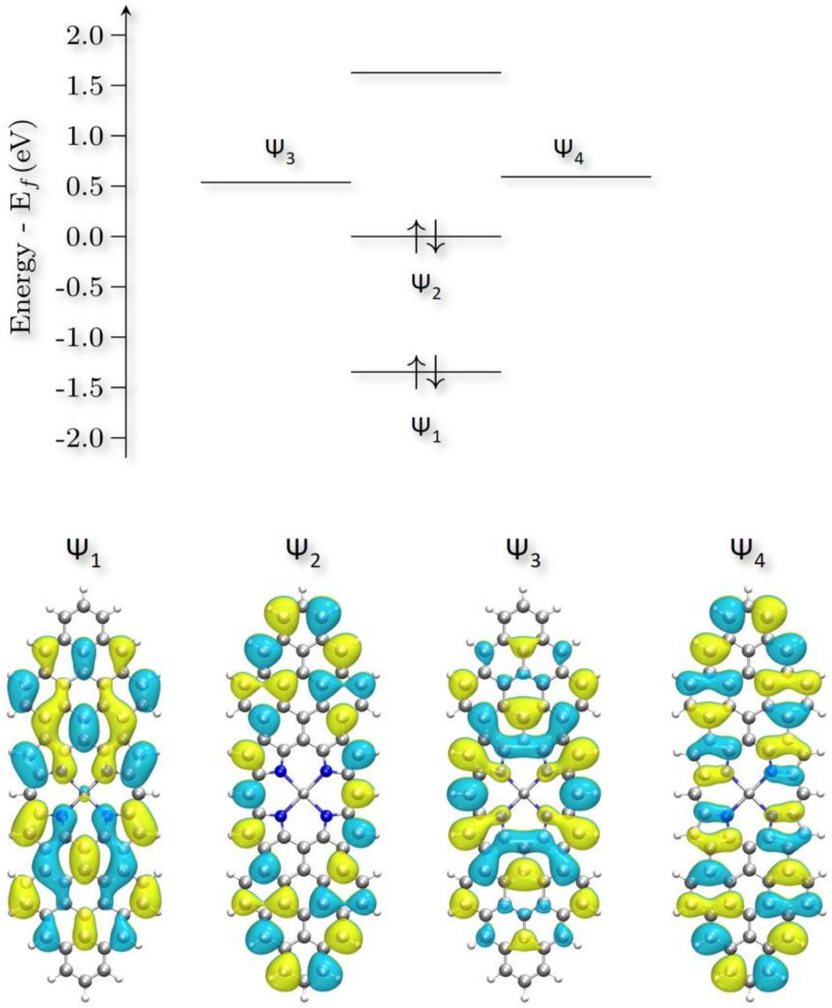

Supplementary Figure 4.3. DFT calculated energy spectrum (top) and molecular orbitals (bottom) of **ZnPorT$_2$** in gas phase under restricted Kohn-Sham conditions at PBE0 level.

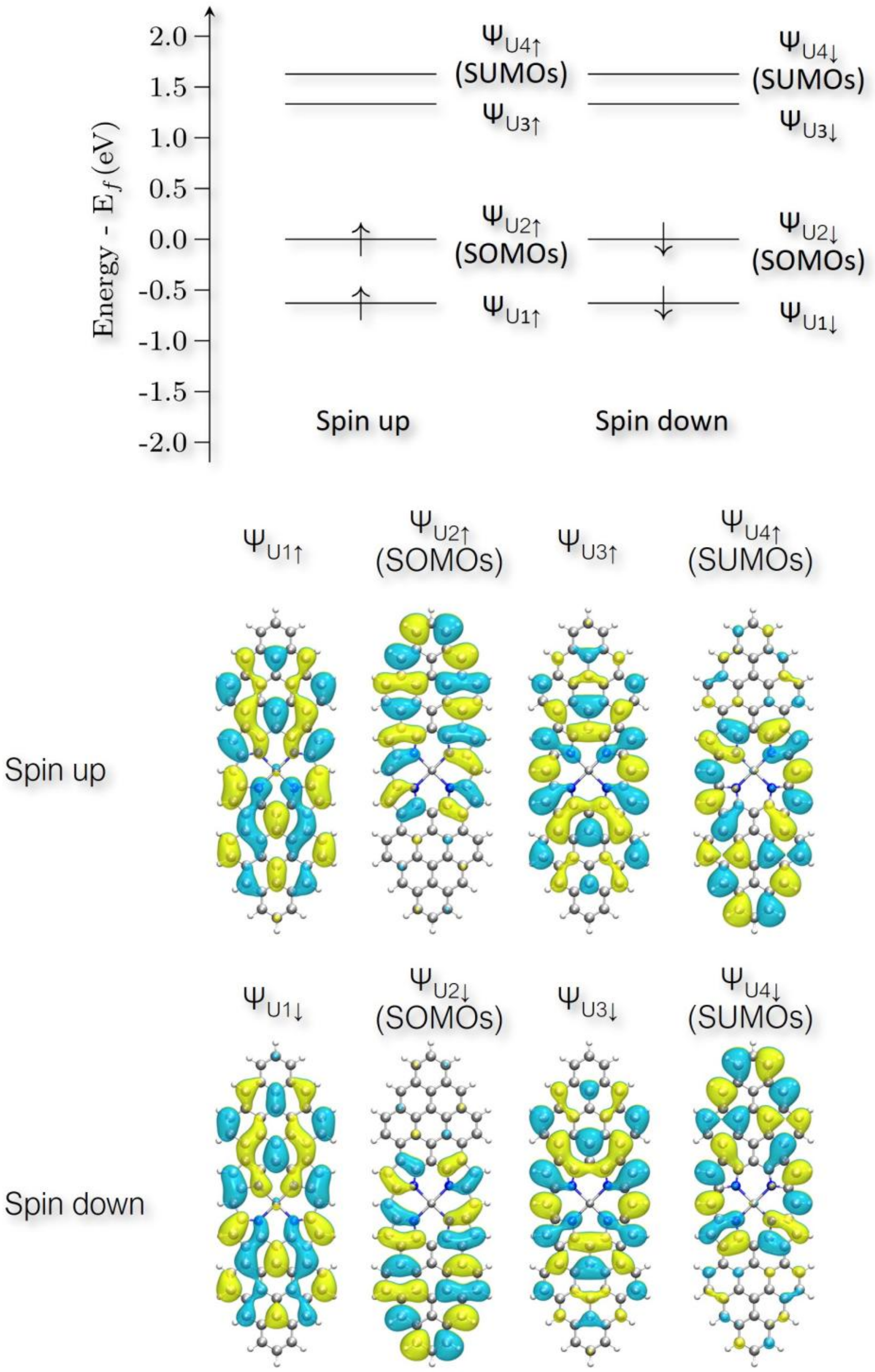

Supplementary Figure 4.4. DFT calculated energy spectrum (top) and molecular orbitals (bottom) of **ZnPorT$_2$** in gas phase under unrestricted Kohn-Sham conditions at PBE0 level. It is worth noting that Ψ_{U2} and Ψ_{U4} are a pair of singly occupied/unoccupied orbitals that are spin-polarized with the electrons mainly localized at the triangulene π-extensions. In contrast, Ψ_{U3} is a doubly unoccupied molecular orbital, primarily contributed by the porphyrin macrocycle.

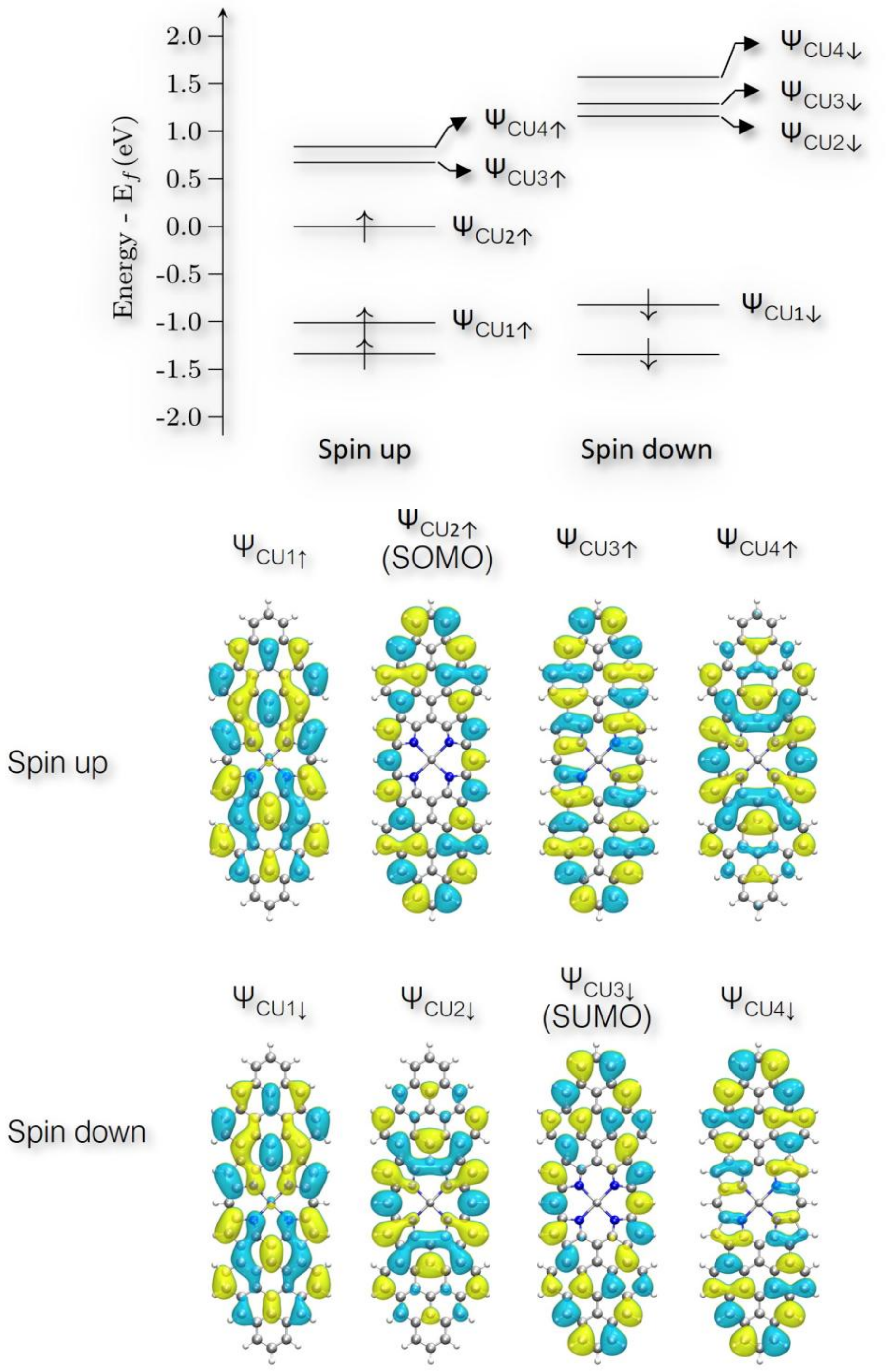

Supplementary Figure 4.5. DFT calculated energy spectrum (top) and molecular orbitals (bottom) of singly positively charged **ZnPorT2** (*i.e.*, **ZnPorT2$^{\bullet+}$**) in gas phase under unrestricted Kohn-Sham conditions at PBE0 level.

The “defective” analogues of **ZnPorT$_2$**, namely **ZnPorT$_2$-d$_1$** and **ZnPorT$_2$-d$_2$** were studied using dI/dV spectroscopy and mapping combined with DFT calculations at PBE level (Supplementary Figs 4.6-4.11). **ZnPorT$_2$-d$_1$** contains one pentagon ring at one of its two triangulene extensions, resulting in a ring contraction that “quenches” the unpaired π-electron formally positioned over this portion of the molecule [27,28]. Consequently, **ZnPorT$_2$-d$_1$** is expected to hold a $S = 1/2$ open-shell ground state. However, for this nanostructure, a prominent resonance is observed just above the Fermi level, with the dominant features mainly localized at the intact triangulene extension (Supplementary Fig. 4.6). This suggests that the molecule is positively charged on Au(111) [29–32], which is well confirmed by UKS DFT calculations of **ZnPorT$_2$-d$_1$** adsorbed on Au(111) (Supplementary Figs 4.7 and 4.8). In the case of **ZnPorT$_2$-d$_2$**, the presence of a pentagon ring on each of the two former triangulene fragments confers a closed-shell ground state to the molecule, with the HOMO and LUMO located at –0.7 and 0.5 V, respectively (Supplementary Figs 4.9-4.11).

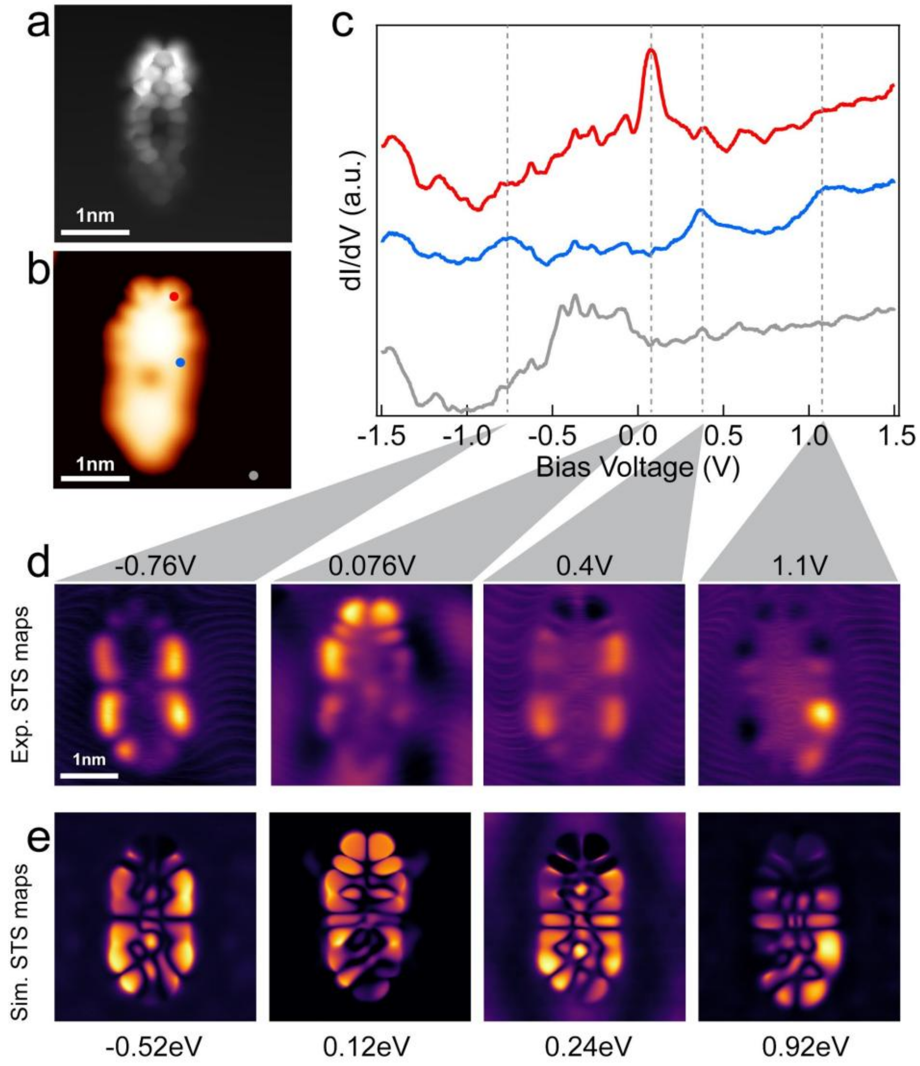

Supplementary Figure 4.6. dI/dV measurements of **ZnPorT$_2$-d$_1$**. (a) BR-STM image of **ZnPorT$_2$-d$_1$** to confirm the chemical structure of the measured species. (b) STM image of **ZnPorT$_2$-d$_1$**. (c) dI/dV spectroscopy measured at the positions shown in (b). (Lock-in amplitude: 20 mV). (d) dI/dV maps taken at the bias voltage shown above each map. (e) Simulated local density of states (LDOS) of **ZnPorT$_2$-d$_1$** on Au(111) (Supplementary Fig. 4.7) at the energy shown below each map. Scanning parameters: (a) I = 200 pA, U = –5 mV. (b) I = 500 pA, U = 80 mV. (d) I = 500 pA. The bias voltage is shown above each map.

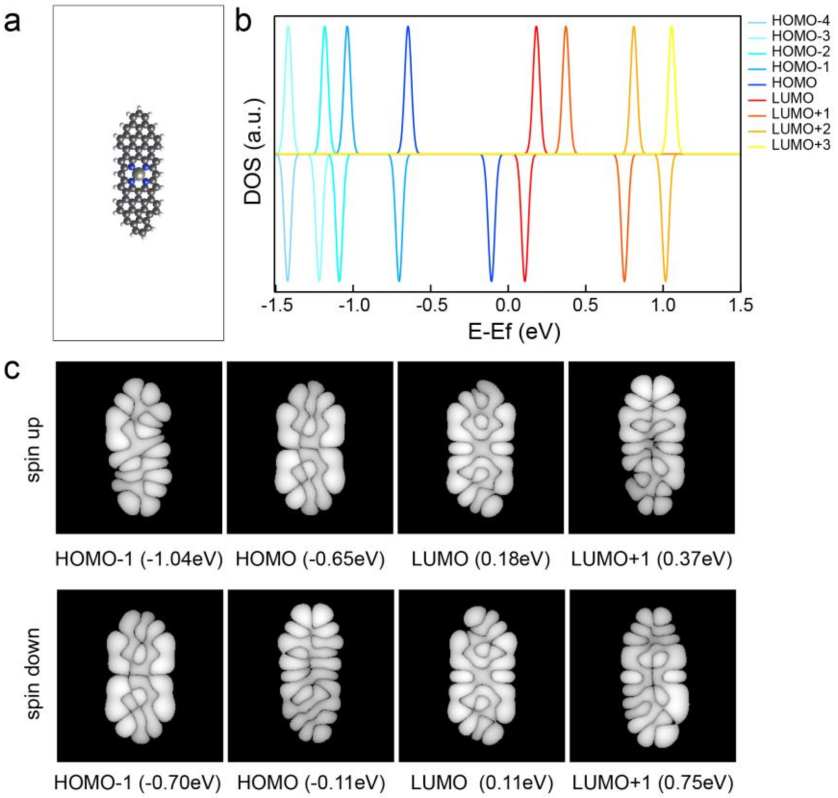

Supplementary Figure 4.7. DFT calculations of **ZnPorT$_2$-d$_1$** in gas phase in unrestricted Kohn-Sham conditions at PBE level. (a) Geometry optimized chemical structure of **ZnPorT$_2$-d$_1$**; (b) Projected density of states (DOS) of different molecular orbitals. Broadening: 0.05eV; (c) Calculated molecular orbitals.

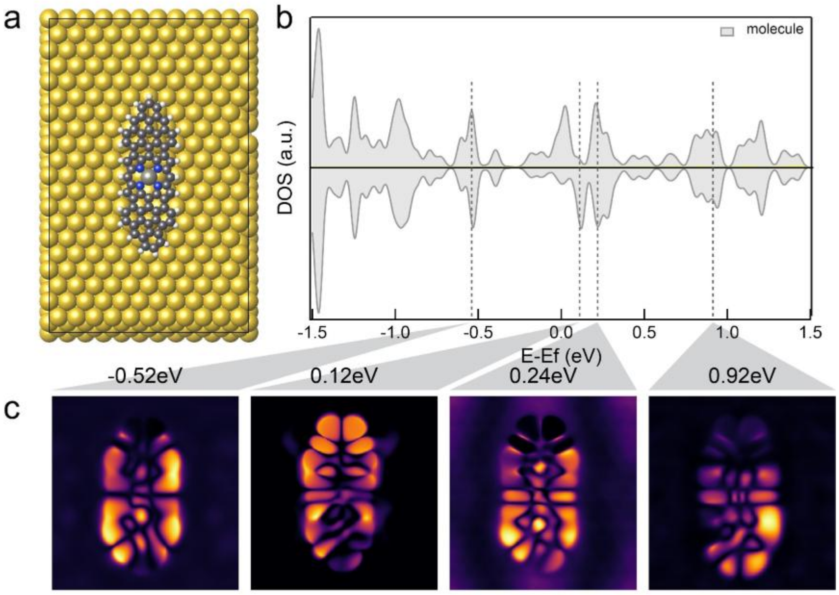

Supplementary Figure 4.8. DFT calculations **ZnPorT2-d1** on Au(111) in unrestricted Kohn-Sham conditions at PBE level. (a) Geometry optimized chemical structure of **ZnPorT2-d1** on a four-layer Au(111) slab; (b) Density of states (DOS). Broadening: 0.05 eV; (c) Calculated local density of states (LDOS) of **ZnPorT2-d1** at the energy shown above each image.

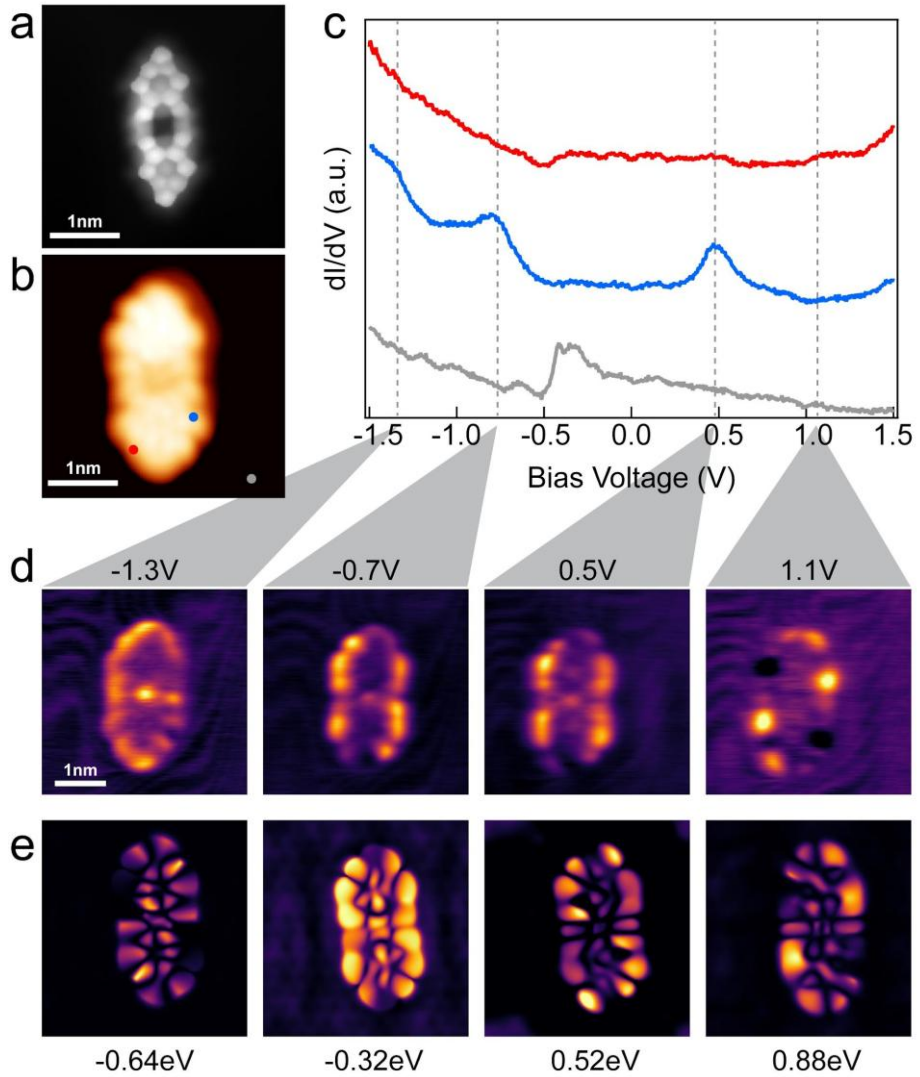

Supplementary Figure 4.9. d*I*/d*V* measurements of **ZnPorT2-d2**. (a) BR-STM and (b) STM images of **ZnPorT2-d2**. (c) d*I*/d*V* spectroscopy on **ZnPorT2-d2** measured at the positions shown in (b) (Lock-in amplitude: 20 mV). (d) d*I*/d*V* maps on **ZnPorT2-d2** taken at the bias voltage shown above each map. (e) Simulated local density of states (LDOS) of **ZnPorT2-d2** on Au(111) (Supplementary Fig. 4.10) at the energy shown below each map. Scanning parameters: (a) I = 100 pA, U = –5 mV; (b) I = 300 pA, U = –1.5 V. (d) I = 300 pA.

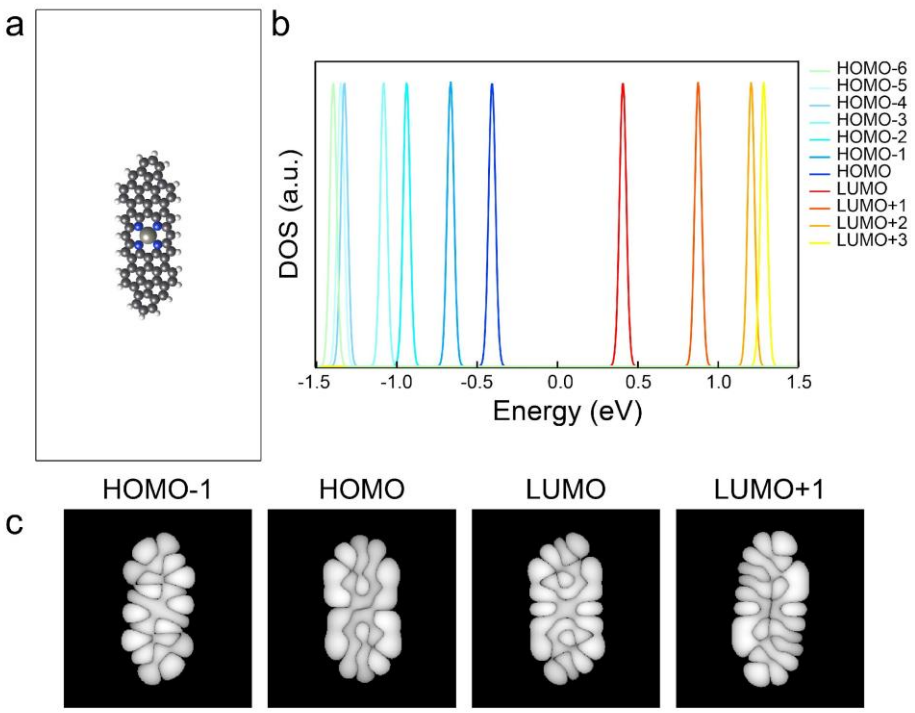

Supplementary Figure 4.10. DFT calculations of **ZnPorT$_2$-d$_2$** in gas phase at PBE level. (a) Geometry optimized chemical structure of **ZnPorT$_2$-d$_2$**. (b) Projected density of states (DOS) into different molecular orbitals. Broadening: 0.05eV; (c) Calculated molecular orbitals.

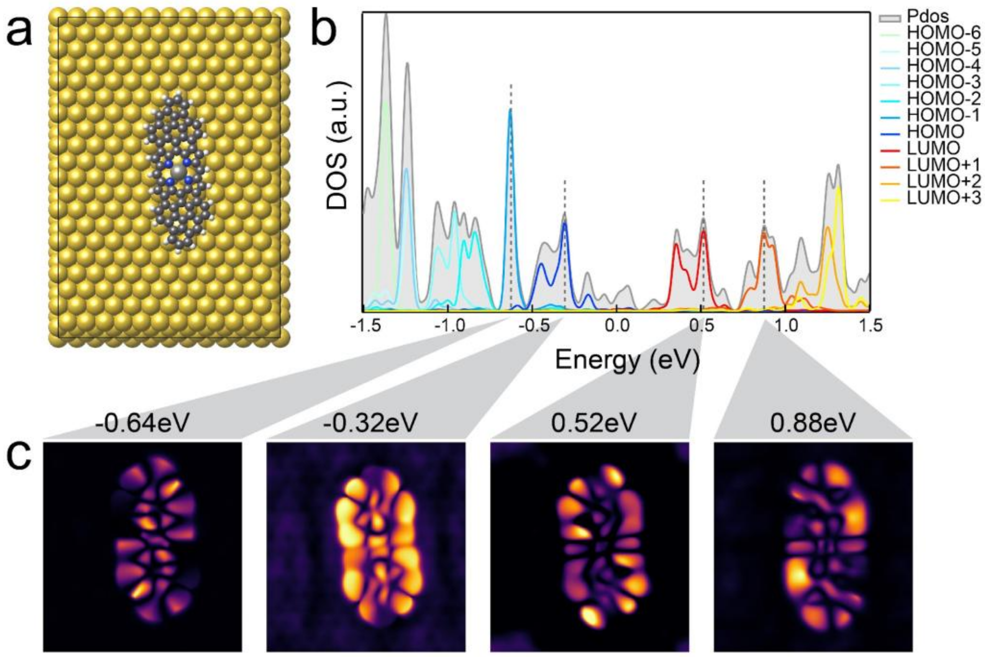

Supplementary Figure 4.11. DFT calculations of **ZnPorT$_2$-d$_2$** on Au(111) at PBE level. (a) Geometry optimized chemical structure of **ZnPorT$_2$-d$_2$** on a four-layer Au(111) slab. (b)

Density of states (DOS) map. Broadening: 0.05eV; (c) Calculated local density of states (LDOS) of **ZnPorT$_2$-d$_2$** at the energy shown for each image.

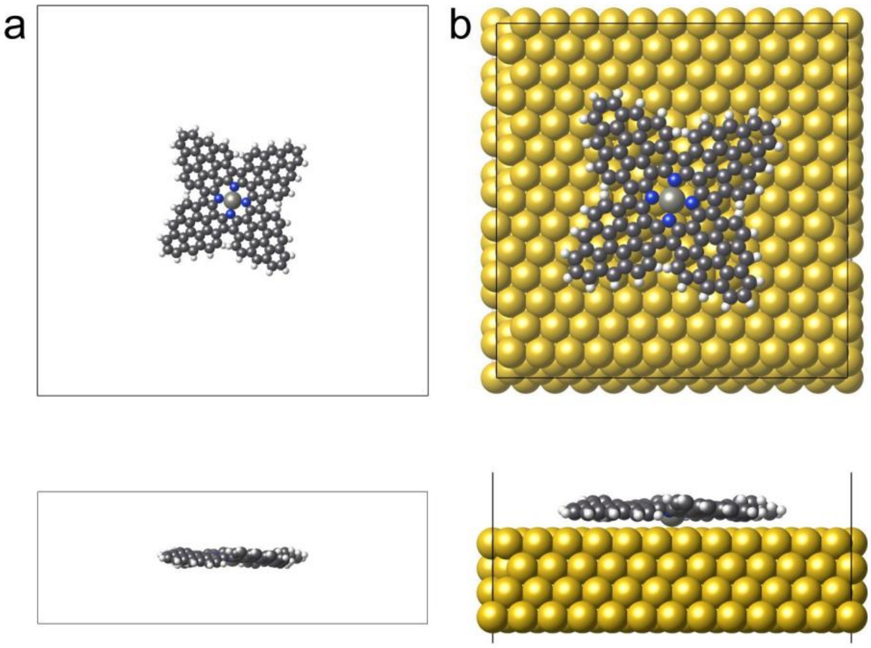

Supplementary Figure 4.12. Geometry optimized structure of **ZnPorT$_4$** by DFT at PBE level (top: upper view with respect to the Por plane, bottom: side view with respect to the Por plane) in (a) gas phase and (b) on a Au(111) slab. The distortion of the four triangulene "arms" is reduced on the Au(111) surface compared to the gas phase. Dark grey, blue, white, grey and yellow balls represent carbon, nitrogen, hydrogen, zinc and gold atoms, respectively.

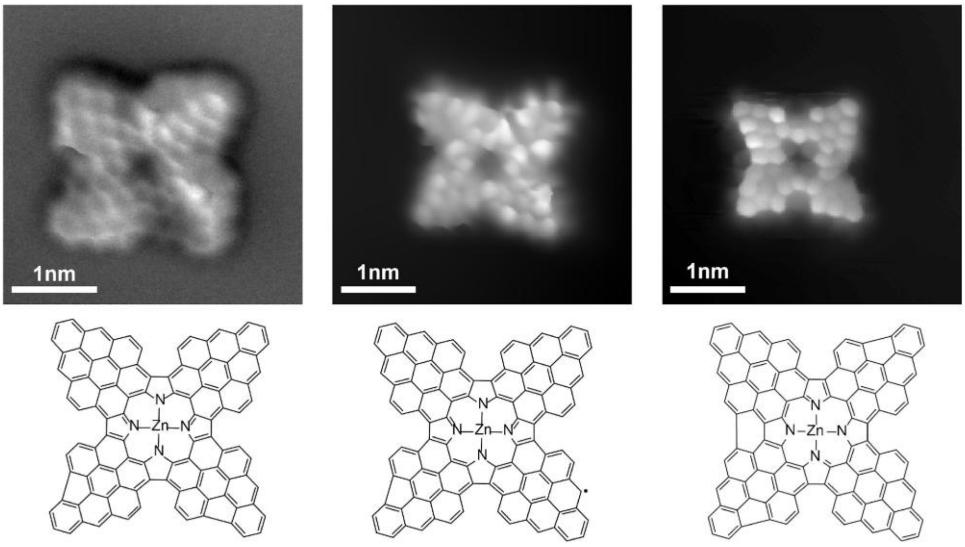

Supplementary Figure 4.13. nc-AFM and BR-STM images of some "defective" **ZnPorT$_4$** analogs which chemical structure is represented in the lower part of the figure. Scanning

parameters: (Left) I = 100 pA, U = –5 mV on top of the molecule before switching off the feedback; (Middle) I = 200 pA, U = –5 mV on top of the molecule before switching off the feedback. (Right) I = 150 pA, U = 5 mV on top of the molecule before switching off the feedback.

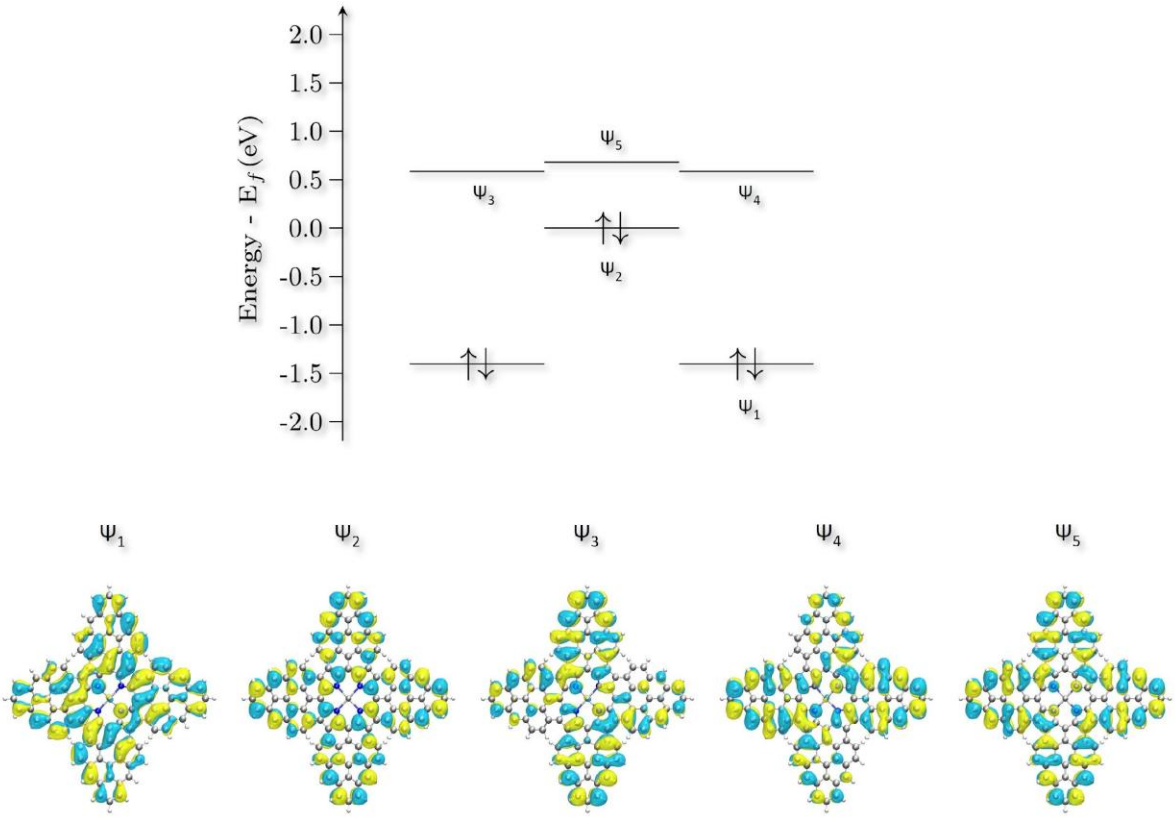

Supplementary Figure 4.14. DFT calculated energy spectrum (top) and molecular orbitals (bottom) of **ZnPorT4** in gas phase under restricted Kohn-Sham conditions at PBE0 level.

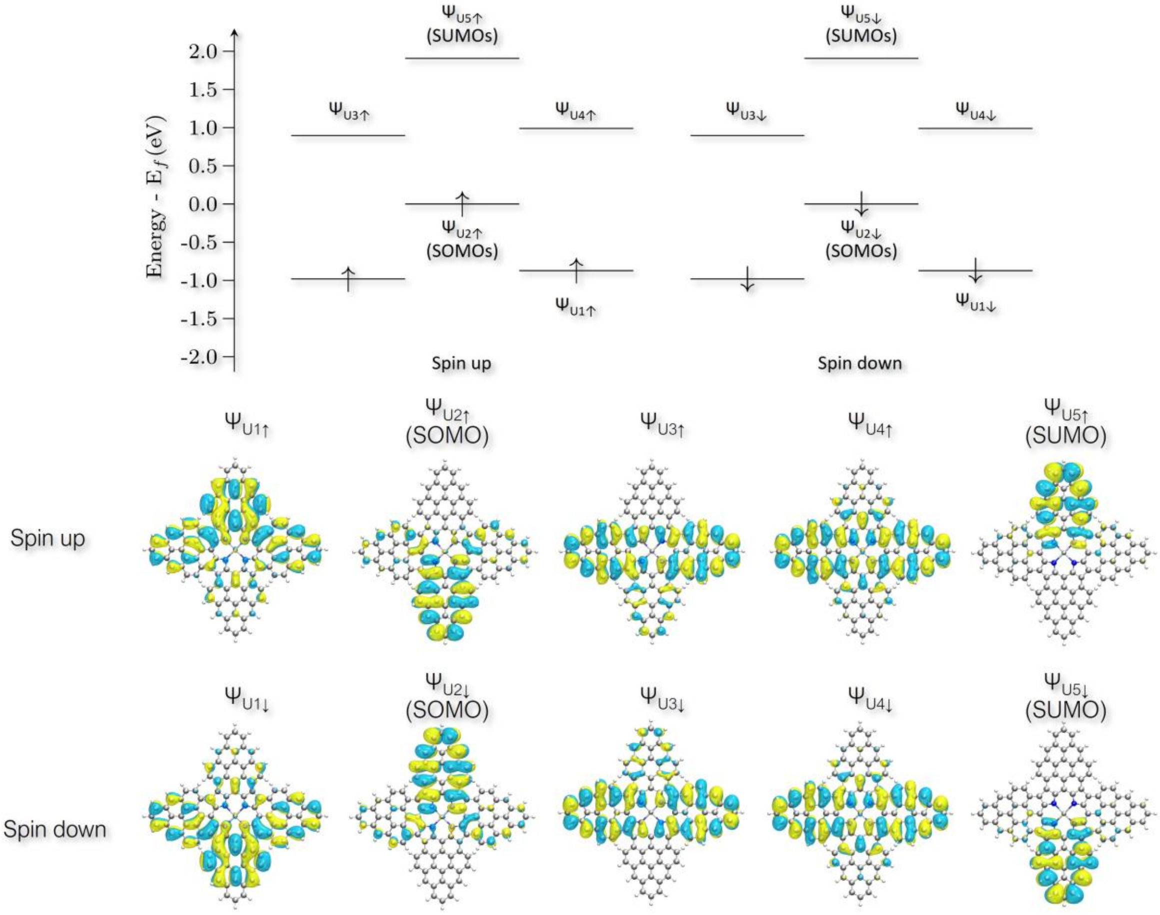

Supplementary Figure 4.15. DFT calculated energy spectrum (top) and molecule orbitals (bottom) of **ZnPorT₄** in gas phase under unrestricted Kohn-Sham conditions at PBE0 level.

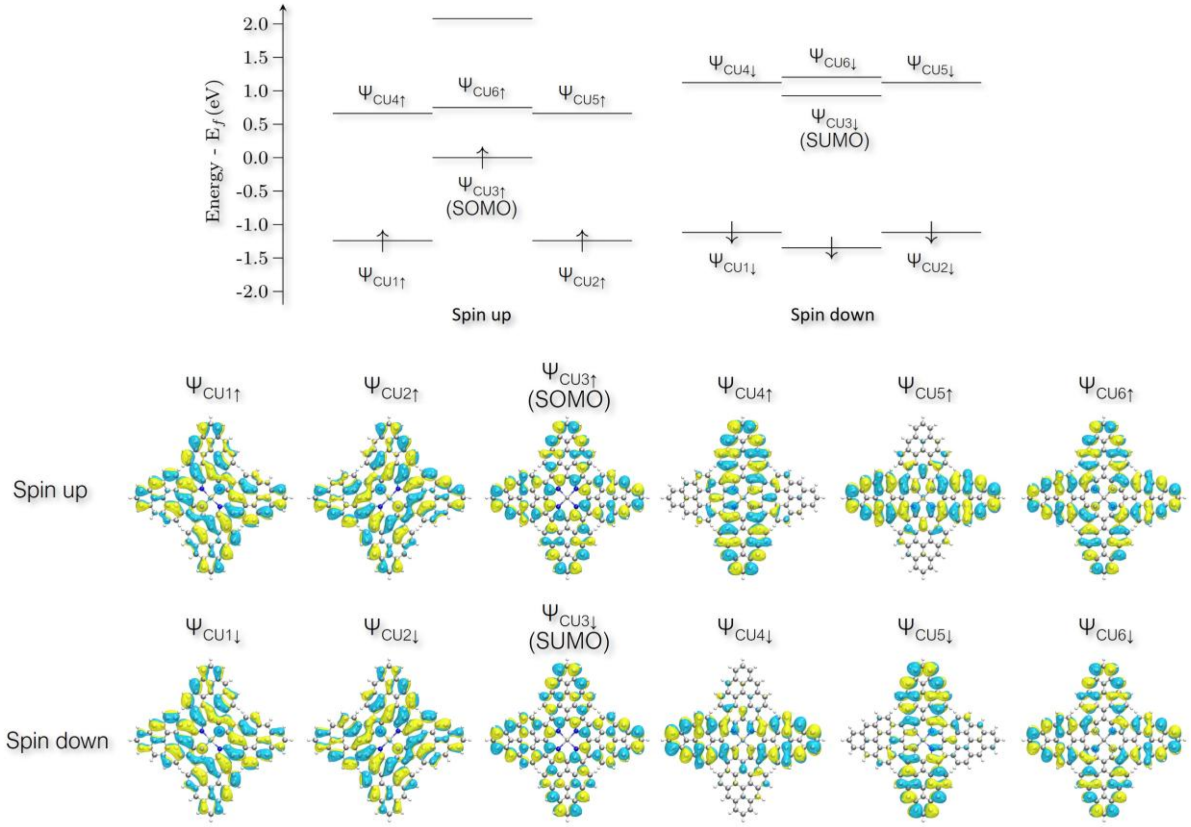

Supplementary Figure 4.16. DFT calculated energy spectrum (top) and molecule orbitals (bottom) of singly positively charged **ZnPorT**$_4$ (*i.e.*, **ZnPorT**$_4^{\bullet+}$) in gas phase under unrestricted Kohn-Sham conditions at PBE0 level.

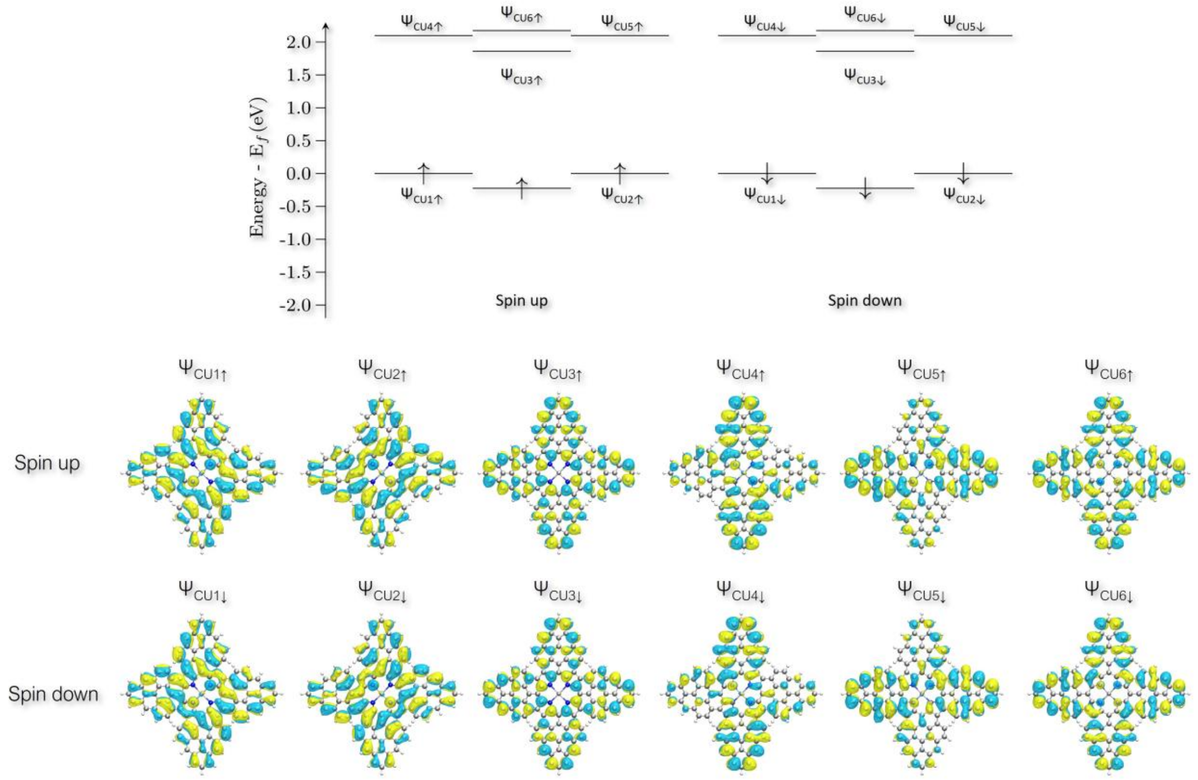

Supplementary Figure 4.17. DFT calculated energy spectrum (top) and molecule orbitals (bottom) of doubly positively charged **ZnPorT$_4$** (*i.e.*, **ZnPorT$_4^{2+}$**) in gas phase under unrestricted Kohn-Sham conditions at PBE0 level.

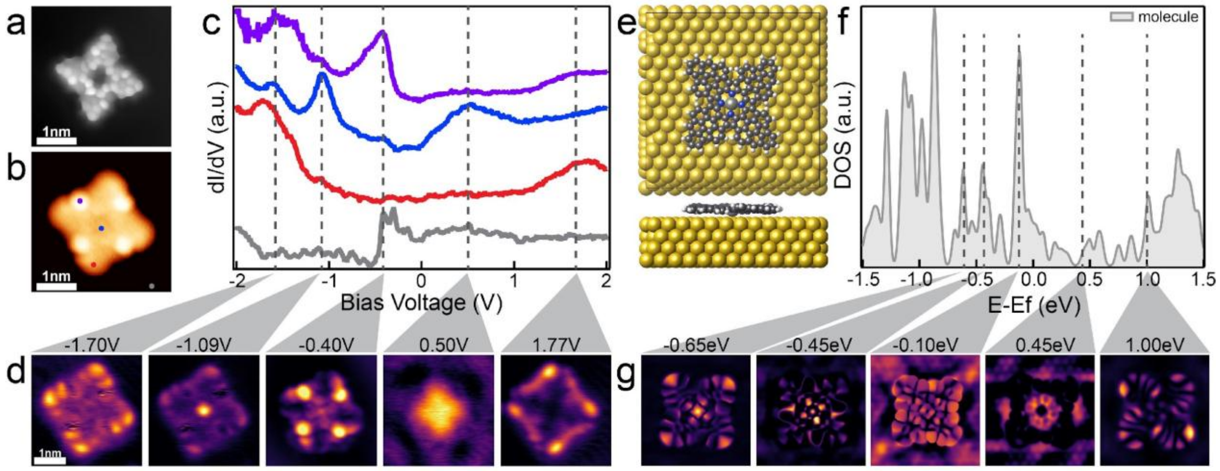

Supplementary Figure 4.18. dI/dV measurements of **ZnPorT$_4$-d$_1$**. (a) BR-STM image of **ZnPorT$_4$-d$_1$** to confirm the chemical structure of the measured species. (b) STM image of **ZnPorT$_4$-d$_1$**. (c) dI/dV spectroscopy measured at the positions shown in (b) (Lock-in amplitude: 20 mV). (d) dI/dV maps taken at the bias voltage shown above each map. (e) Geometry optimized chemical structure of **ZnPorT$_4$-d$_1$** (top: upper view with respect to the Por plane, bottom: side view with respect to the Por plane) on Au(111) at PBE level. (f) Restricted Kohn-

Sham DFT calculations of DOS of **ZnPorT$_4$-d$_1$**. Broadening: 0.05 eV. (g) Simulated local density of states (LDOS) of **ZnPorT$_4$-d$_1$** on Au(111) at the energy shown above each map. Scanning parameters: (a) *I* = 150 pA, *U* = 5 mV on top of the molecule before switching off the feedback; (b) *I* = 100 pA, *U* = –1.0 V; (d) *I* = 300 pA. The bias voltage is shown above each map.

S5 Many-body calculations

Neutral $ZnPorT_2$ ground state properties

Spin-polarized calculations indicate that **$ZnPorT_2$** possesses a singlet ground state, with spin density predominantly localized along the zigzag edges of the triangulene subunits (see Supplementary Fig. 5.1). This distribution reflects the molecule's diradicaloid character, arising from the fusion of triangulene units at the Por "edge". The observed spin localization aligns with theoretical predictions for triangulene-based systems, where the electronic structure is influenced by the topology and conjugation of the π-system.

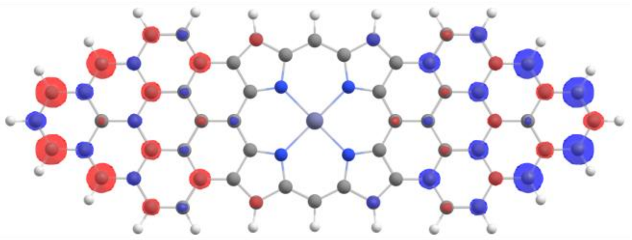

Supplementary Figure 5.1. Spin density distribution of **$ZnPorT_2$** obtained from singlet spin state BS-DFT calculations using the M06-2X functional. The isosurface is plotted at a contour level of 0.005.

The singlet–triplet energy gap (ΔE_{ST}) of **$ZnPorT_2$** was evaluated using broken-symmetry (BS) DFT calculations at the PBE0 and M06-2X levels of theory. Both singlet and triplet spin configuration geometry were optimized. Due to the inherent spin contamination in BS-DFT approaches, where the singlet state is not a pure spin eigenfunction, we employed Yamaguchi's approximate spin projection method [33,34] to correct the computed energies.

$$\Delta E_{ST} = \frac{2(E_{BS} - E_T)}{\langle S^2 \rangle_T - \langle S^2 \rangle_{BS}}$$

Supplementary Table 5.1. Singlet-triplet energy gap of **$ZnPorT_2$** computed by DFT calculations.

Theory	$E_{BS} - E_T$ [meV]	ΔE_{ST}[meV]
PBE0	–39.418	–90.646
M062X	–19.190	–39.962

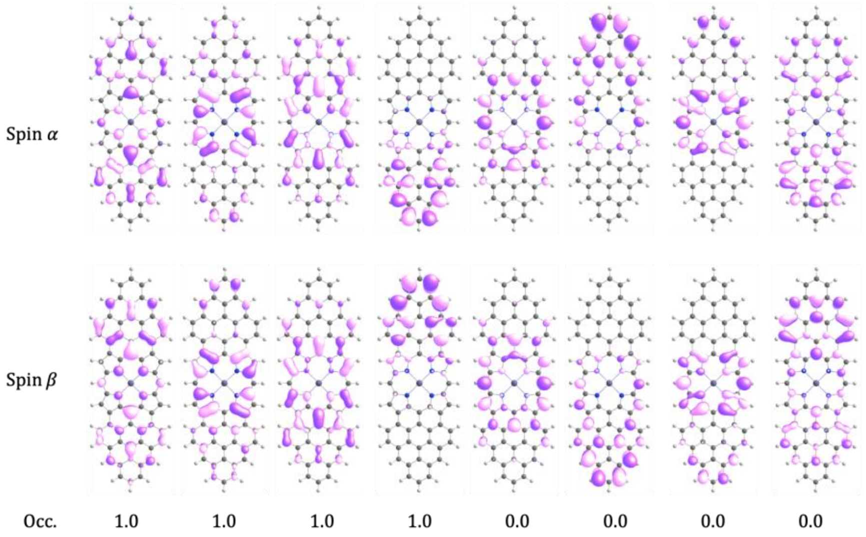

Supplementary Figure 5.2. Kohn–Sham molecular orbitals from the M06-2X/BS DFT simulation of **ZnPorT**$_2$ in its singlet ground state. The first row displays the α molecular orbitals, while the second row shows the β molecular orbitals. The occupation numbers of the molecular orbitals are displayed at the bottom.

Multireference ZnPorT$_2$ **ground state properties**

State-specific CASSCF calculations were performed on **ZnPorT**$_2$ for the singlet ground state to characterize the diradical nature of the electronic structure. The configuration state function (CSF) analysis reveals dominant contributions from two nearly degenerate configurations, consistent with the diradical character. In the CAS (8,8) active space, the leading CSFs are $|22220000\rangle$ (48.0 %) and $|22202000\rangle$ (43.1 %). Similarly, for the CAS (12,12) space, the primary contributions arise from $|222222000000\rangle$ (44.8%) and $|222220200000\rangle$ (40.3 %). The slightly larger weight of the first configuration in each case suggests that while the system exhibits significant diradical character, it does not fall strictly into the category of a strong or pure diradical. This is captured in the Natural Orbital occupation analysis (Supplementary Fig. 5.3)

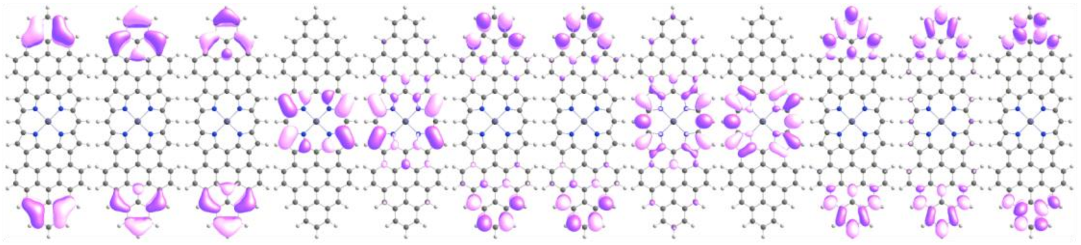

Nos (Occ.) 1.9821 1.9536 1.9536 1.9354 1.9311 1.0491 0.9516 0.0734 0.0602 0.0462 0.0456 0.0178

Supplementary Figure 5.3. SS-CASSCF CAS (12,12) for the neutral ground state of **ZnPorT**$_2$. Natural Orbital occupation numbers are shown below for each orbital.

Singlet-triplet bandgap in ZnPorT$_2$

State-average (SA) CASSCF calculations were conducted to evaluate the singlet triplet energy gap in **ZnPorT**$_2$, including two singlet and two triplet states in the averaging wavefunction. Three different activate spaces were considered in order to evaluate the influence of the NEVPT2 dynamical correlation. The results are summarized in Supplementary Table 5.2.

Supplementary Table 5.2. Singlet-triplet energy difference for **ZnPorT**$_2$ using three different active spaces. Energies are reported at both the state-average CASSCF and DLPNO-NEVPT2 levels of theory.

	Singlet-triplet energy gap ($\Delta E = E_T - E_S$) calculated by SA-CASSCF [meV]	Singlet-triplet energy gap ($\Delta E = E_T - E_S$) calculated by DLPNO-NEVPT2 [meV]
CAS (8,8)	19	30
CAS (10,10)	30	31
CAS (12,12)	26	41

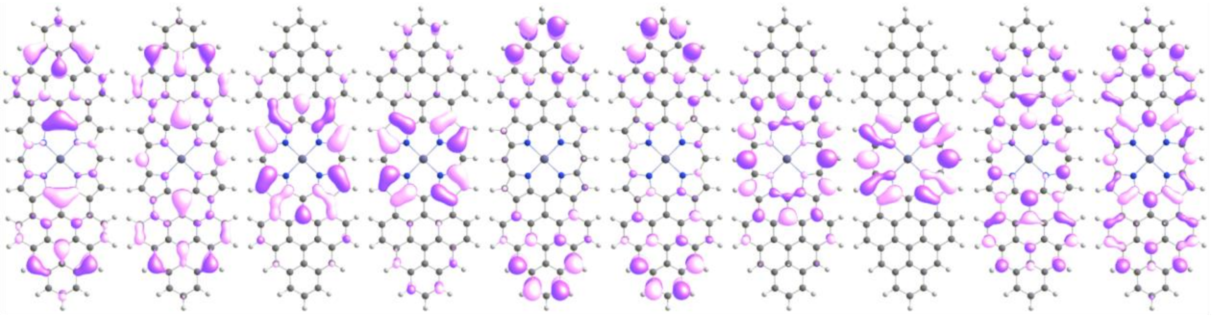

Supplementary Figure 5.4. SA-CASSCF (12,12) Natural orbitals averaged for two singlet and two triplet states of **ZnPorT**$_2$.

$ZnPorT_2$ singly positively charged state properties

State-specific CASSCF calculations were performed for the doublet cationic state of **$ZnPorT_2$** to characterize its multireference character. Configuration State Function (CSF) analysis reveals a dominant contribution from a single configuration in each active space. For CAS (7,8), the leading CSF is |22210000⟩, contributing 91.62 % to the wavefunction. In the CAS (9,10) active space, the same configuration contributes 89.28 %, while for CAS (11,12), the dominant CSF is |222221000000⟩, with a weight of 86.66 %.

All three calculations consistently reveal the presence of a single unpaired electron, as further supported by the natural orbital occupation numbers. The singly occupied natural orbital is localized at the edge of the porphyrin ring and extends over the triangulene.

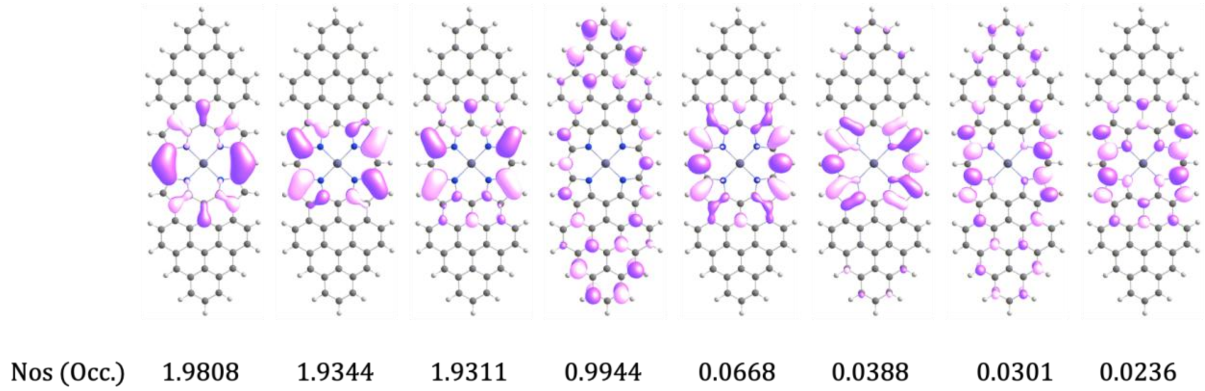

Supplementary Figure 5.5. SS-CASSCF (7,8) orbitals for the positively charged doublet spin state of **$ZnPorT_2^{\bullet +}$**. Natural Orbital occupation numbers are shown below for each orbital.

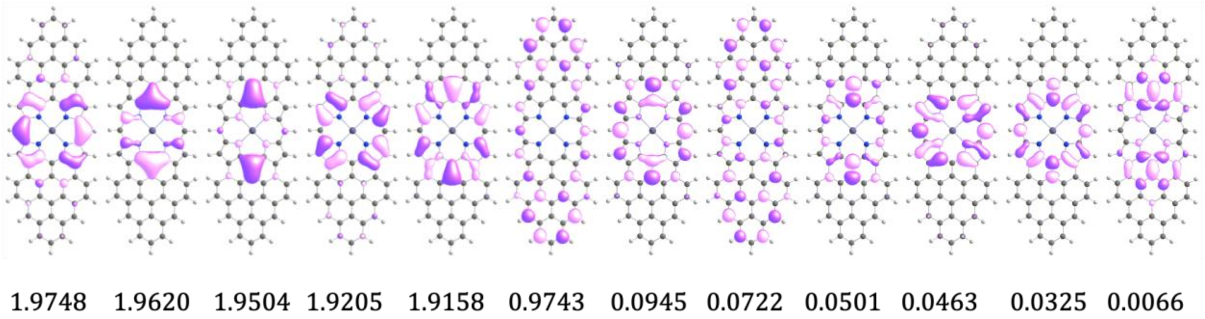

Supplementary Figure 5.6. Optimized SS-CASSCF(11,12) orbitals for the positively charged doublet spin state of **$ZnPorT_2^{\bullet +}$**. Natural Orbital occupation numbers are shown below each orbital.

Dysons and Kondo orbitals of positively charged species $ZnPorT_2^{\bullet+}$ and Dyson orbitals for doubly positively charged species $ZnPorT_4^{2+}$.

To interpret the d*I*/d*V* maps associated with positive (negative) ionic resonances observed in the experiment, we computed Dyson orbitals [35] corresponding to a temporal electron removal (attachment) process during the tunneling process between the STM tip and the molecule. Detailed description of the methodology of multireference Dyson orbitals describing the virtual electronic transitions between the ground state of the neutral system and the N±1 charge states can be found here [36]. To capture the single charge transfer from **$ZnPorT_2$** to the substrate, we considered the singly positively charged species **$ZnPorT_2^{\bullet+}$** as reference N state. Similarly, for the **$ZnPorT_4$**, to account for the double-charge transfer we have considered the doubly positively charged species **$ZnPorT_4^{2+}$** as reference N state. To compute the Dyson orbitals, we first obtained one- and two-electron integrals within an active space of 11 electrons in 11 orbitals CAS(11,11) in the ground state that is positively charged for the **$ZnPorT_2$**, and CAS(12,12) for **$ZnPorT_4^{2+}$** using the orbitals obtained from DFT-PBE [37] (see Supplementary Figs 5.7 and 5.13) from quantum chemistry software ORCA [11]. Using these integrals, we constructed the full many-body Hamiltonian and diagonalized it to get a complete active space configuration interaction (CASCI) wavefunction, yielding the wavefunction of the N ground state. We then repeated the CASCI calculation for the ionized systems (N±1 electrons with reference from the positively charged ground state) to obtain their corresponding wavefunctions. Finally, the Dyson orbitals were calculated from the overlap between the neutral and charged state wavefunctions, describing the transition associated with the electron addition or removal process. The resulting Dyson orbitals for electron removal and attachment processes are presented in Supplementary Figs 5.8 and 5.9, respectively. Theoretically simulated d*I*/d*V* maps based on the Dyson orbitals with the spectral function obtained using the Probe Particle STM code [38] are shown in Supplementary Fig. 5.10. Furthermore, to analyze the features associated with the Kondo resonance in the d*I*/d*V* spectra, we computed the Kondo orbitals from the CASCI wavefunction as described in Ref [39]. There is only one anti-ferromagnetic channel for the Kondo screening shown in Supplementary Fig. 5.11. Simulation of d*I*/d*V* maps of Dyson and Kondo orbitals is performed using the PPSTM code for a metal-like tip [4].

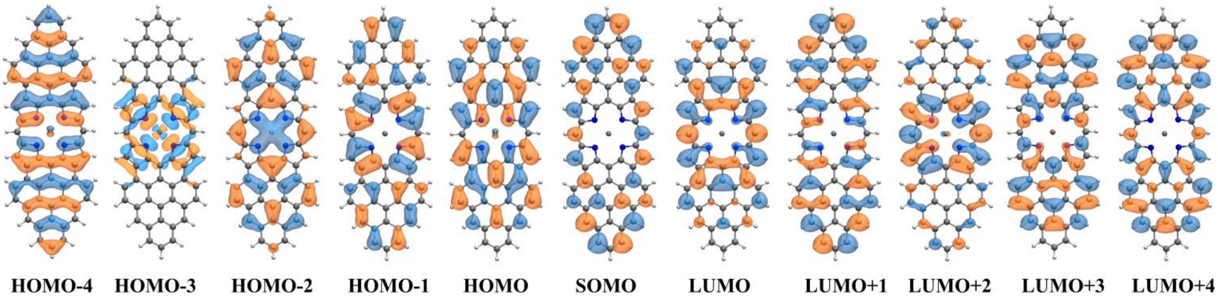

Supplementary Figure 5.7. DFT-PBE orbitals used in CASCI calculation for singly positively charged species **ZnPorT**$_2^{\bullet+}$.

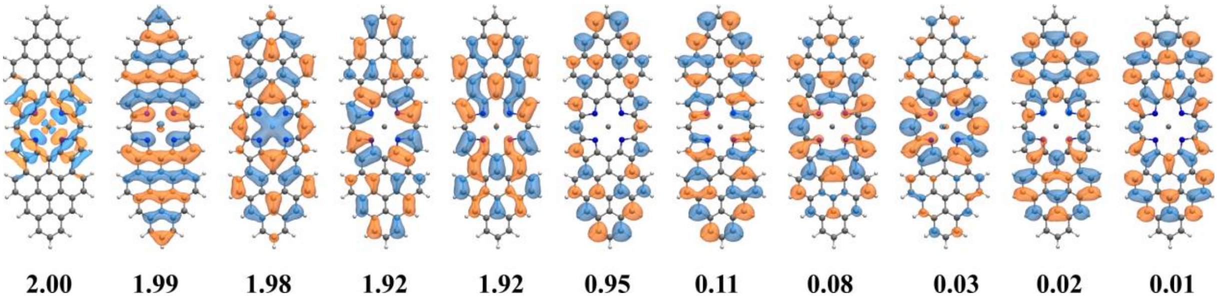

Supplementary Figure 5.8. Multireference natural orbitals obtained from CASCI calculation with the occupation below for singly positively charged species **ZnPorT**$_2^{\bullet+}$.

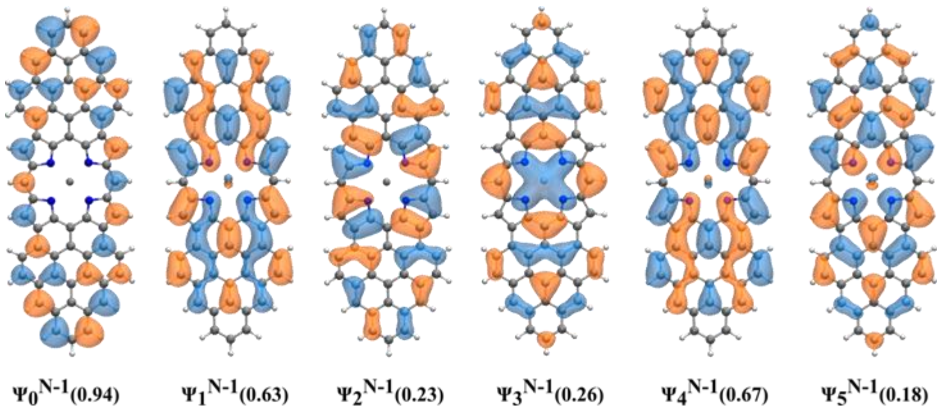

Supplementary Figure 5.9. Dyson orbitals for the process of removal of an electron with the norm of the wavefunction for singly positively charged species **ZnPorT**$_2^{\bullet+}$.

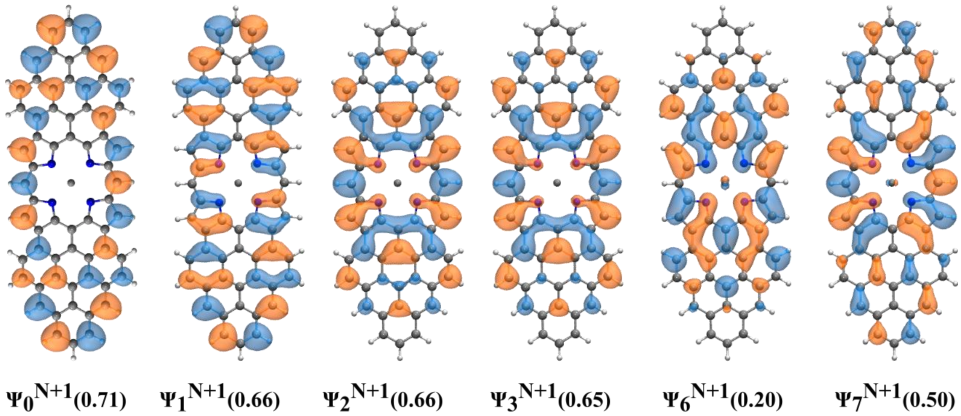

Supplementary Figure 5.10. Dyson orbitals for the process of adding an electron with the norm of the wavefunction for singly positively charged species **ZnPorT**$_2^{•+}$.

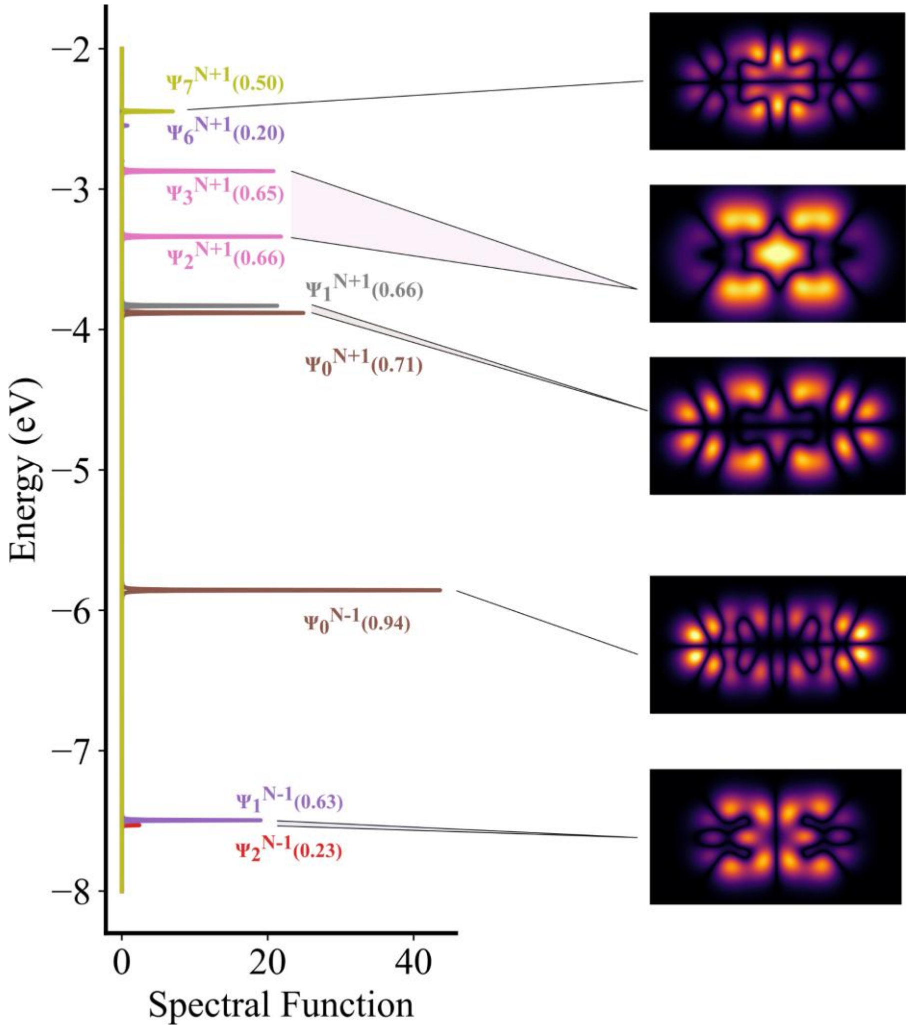

Supplementary Figure 5.11. Calculated spectral function using the Green's function formalism and corresponding simulated dI/dV maps of Dyson orbitals shown in Supplementary Fig. 5.9 (electron removal) and Supplementary Fig. 5.10 (electron addition) for singly positively charged species **ZnPorT**$_2^{•+}$.

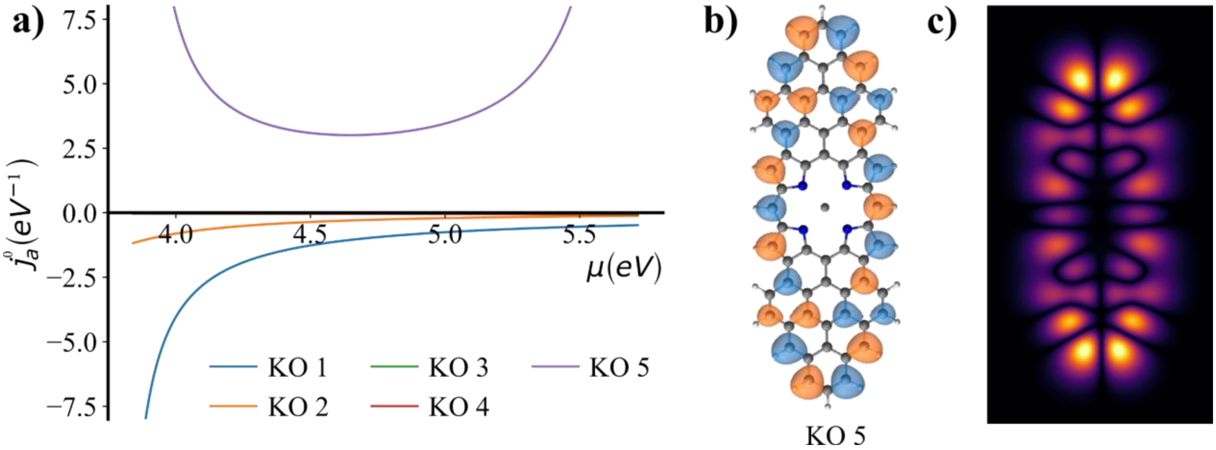

Supplementary Figure 5.12. Results from the multi-orbital Kondo analysis on **ZnPorT$_2$**, used to simulate the Kondo spatial distribution map. (a) Coupling constants were computed as a function of the chemical potential for each Kondo orbital (KO). (b) Orbital isosurface of only KO with non-zero coupling to the conduction electrons of the substrate at a chemical potential of 4.7 eV. (c) Simulated d*I*/d*V* map of antiferromagnetically coupled Kondo orbital.

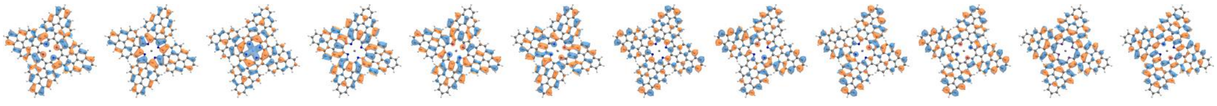

HOMO-5 HOMO-4 HOMO-3 HOMO-2 HOMO-1 HOMO LUMO LUMO+1 LUMO+2 LUMO+3 LUMO+4 LUMO+5

Supplementary Figure 5.13. DFT-PBE orbitals used in CASCI calculation for doubly positive charged species **ZnPorT$_4$$^{2+}$**.

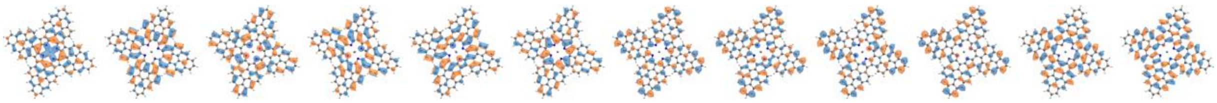

1.99 1.99 1.98 1.97 1.97 1.96 0.04 0.03 0.03 0.03 0.01 0.01

Supplementary Figure 5.14. Multireference natural orbitals obtained from CASCI calculation with the occupation below for doubly positive charged species **ZnPorT$_4$$^{2+}$**.

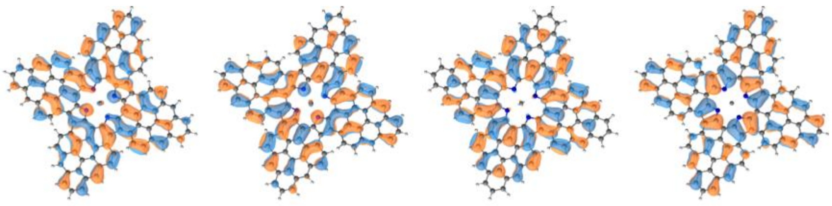

Ψ_0^{N-1}(0.93) Ψ_1^{N-1}(0.93) Ψ_2^{N-1}(0.93) Ψ_3^{N-1}(0.90)

Supplementary Figure 5.15. Dyson orbitals for the process of removal of an electron with the norm of the wavefunction for doubly positive charged species **ZnPorT$_4$$^{2+}$**.

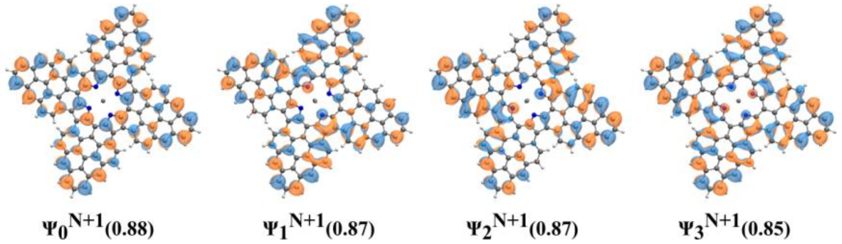

Supplementary Figure 5.16. Dyson orbitals for the process of adding an electron with the norm of the wavefunction for doubly positively charged species **ZnPorT**$_4^{2+}$.

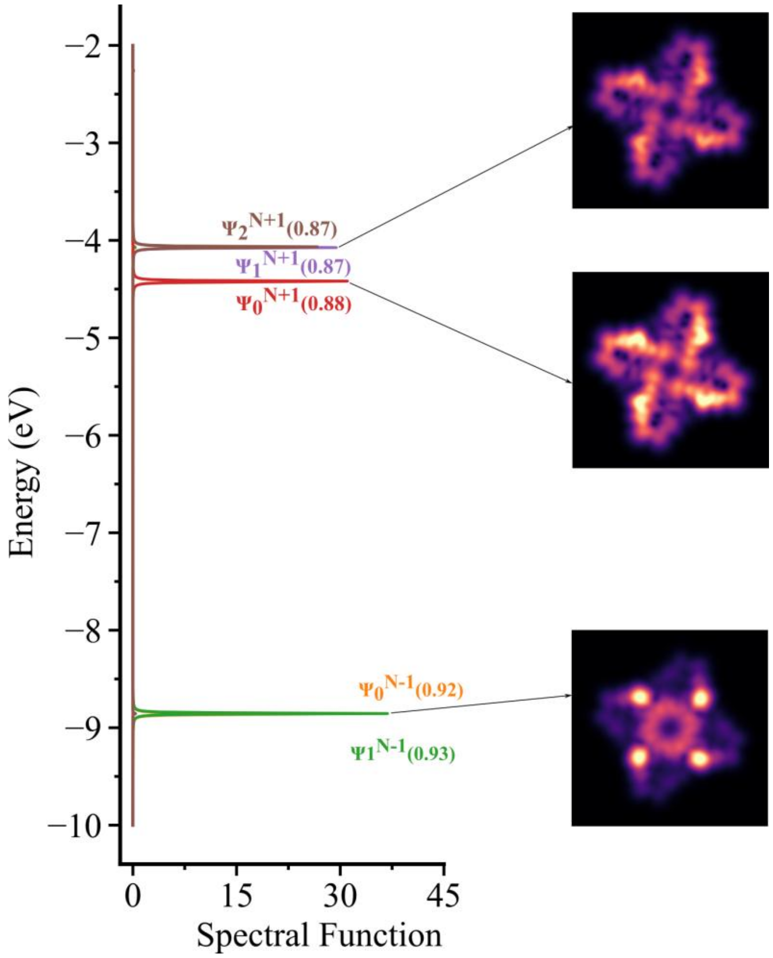

Supplementary Figure 5.17. Calculated spectral function using the Green's function formalism and corresponding simulated d*I*/d*V* maps of Dyson orbitals shown in Supplementary Fig. 5.15 (electron removal) and Supplementary Fig. 5.16 (electron addition) for doubly positive charged species **ZnPorT**$_4^{2+}$.

S6 Charge analysis

Quantifying charge transfer for open-shell adsorbates on substrates using DFT simulations is an inherently challenging tack due to the large system size and the complexity of molecule–metal interfaces. Commonly employed population analysis methods, such as Mulliken population analysis (MPA) and Hirshfeld population analysis (HPA), often yield different and sometimes even contradictory results. In the case of MPA, the calculated charges are highly sensitive to the choice of basis set. HPA, while generally more stable with respect to basis-set variations than MPA, assigns charges based on reference neutral atomic densities. As a result, the effective electron-donating or electron-withdrawing strength of an atom—strongly influenced by its local chemical environment—is not fully captured [40]. More reliable estimates can, in principle, be obtained within Bader's Quantum Theory of Atoms in Molecules (QTAIM) [41]. Bader charge analysis (BCA) provides a real-space, density-based partitioning scheme that is well suited for plane-wave DFT calculations. However, the accuracy of BCA critically depends on the quality and resolution of the real-space charge-density grid. Achieving well-converged Bader charges for large molecule–metal interface systems such as **ZnPorT$_2$**/Au(111) and **ZnPorT$_4$**/Au(111) is therefore computationally demanding. In the present work, we pushed the calculations as far as possible by employing charge densities computed on extremely fine real-space grids, corresponding to a plane-wave cutoff of 1200 Ry for the charge-density expansion. Under these conditions, Bader analysis yields charge transfers of approximately 1.01 e^- for **ZnPorT$_2$** and 1.44 e^- for **ZnPorT$_4$**, indicating a larger charge transfer for **ZnPorT$_4$** compared to **ZnPorT$_2$**. A comparison of the charge-transfer values obtained using different charge analysis schemes for **ZnPorT$_x$** (x = 2, 4) is provided in Supplementary Table 6.1. All three charge analysis methods predict charge transfer from the molecules to the substrates, consistent with experimental observations. We further plotted the electron density difference of both showing that the electron depletion is taking place at the molecule, while the slab underneath the molecule shows electron accumulation (Supplementary Fig. 6.1).

Supplementary Table 6.1 Charge analysis of **ZnPorT$_2$** and **ZnPorT$_4$** using different charge analysis methods.

	Mulliken population analysis	Hirshfeld charges	Bader charge analysis
**ZnPorT$_2$**	1.05e	6.94e	1.01e
**ZnPorT$_4$**	1.39e	10.11e	1.44e

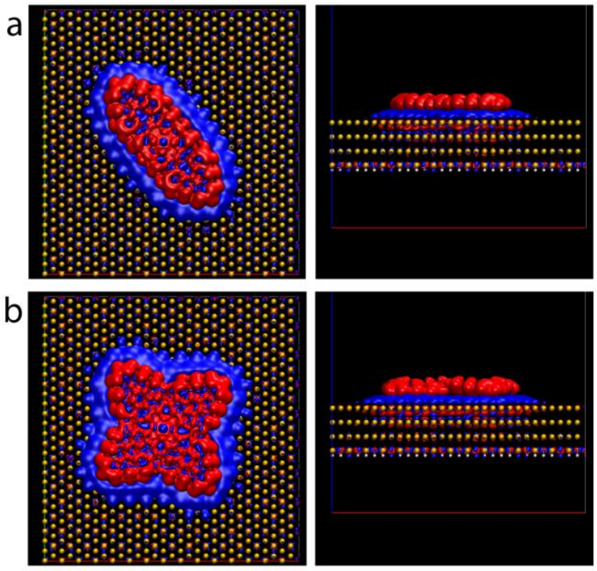

Supplementary Figure 6.1 Electron density difference of a) **ZnPorT$_2$** and b) **ZnPorT$_4$** on Au(111) slab. Color code: red and blue represent the depletion and accumulation of electrons, respectively. Isovalue: 0.0001 e/Å.

S7 References

1. Yakutovich AV, Eimre K, Schütt O *et al.* AiiDAlab – an ecosystem for developing, executing, and sharing scientific workflows. *Comput Mater Sci* 2021; **188**: 110165.
2. Pizzi G, Cepellotti A, Sabatini R *et al.* AiiDA: automated interactive infrastructure and database for computational science. *Comput Mater Sci* 2016; **111**: 218–30.
3. Kühne TD, Iannuzzi M, Del Ben M *et al.* CP2K: An electronic structure and molecular dynamics software package - Quickstep: Efficient and accurate electronic structure calculations. *J Chem Phys* 2020; **152**:1 94103.
4. Hapala P, Kichin G, Wagner C *et al.* Mechanism of high-resolution STM/AFM imaging with functionalized tips. *Phys Rev B* 2014; **90**: 085421.
5. Adamo C, Barone V. Toward reliable density functional methods without adjustable parameters: The PBE0 model. *J Chem Phys* 1999; **110**: 6158–70.
6. Weber V, VandeVondele J, Hutter J *et al.* Direct energy functional minimization under orthogonality constraints. *J Chem Phys* 2008; **128**: 084113.
7. Martyna GJ, Tuckerman ME. A reciprocal space based method for treating long range interactions in ab initio and force-field-based calculations in clusters. *J Chem Phys* 1999; **110**: 2810–21.
8. Goedecker S, Teter M, Hutter J. Separable dual-space Gaussian pseudopotentials. *Phys Rev B* 1996; **54**: 1703–10.
9. VandeVondele J, Hutter J. Gaussian basis sets for accurate calculations on molecular systems in gas and condensed phases. *J Chem Phys* 2007; **127**: 114105.
10. Guidon M, Hutter J, VandeVondele J. Auxiliary density matrix methods for Hartree-Fock exchange calculations. *J Chem Theory Comput* 2010; **6**: 2348–64.
11. Neese F, Wennmohs F, Becker U *et al.* The ORCA quantum chemistry program package. *J Chem Phys* 2020; **152**: 224108.
12. Kollmar C, Sivalingam K, Helmich-Paris B *et al.* A perturbation-based super-CI approach for the orbital optimization of a CASSCF wave function. *J Comput Chem* 2019; **40**: 1463–70.
13. Neese F. An improvement of the resolution of the identity approximation for the formation of the Coulomb matrix. *J Comput Chem* 2003; **24**: 1740–7.
14. Guo Y, Sivalingam K, Valeev EF *et al.* SparseMaps—A systematic infrastructure for reduced-scaling electronic structure methods. III. Linear-scaling multireference domain-based pair natural orbital N-electron valence perturbation theory. *J Chem Phys* 2016; **144**: 094111.

15. Shapiro ND, Rauniyar V, Hamilton GL *et al.* Asymmetric additions to dienes catalysed by a dithiophosphoric acid. *Nature* 2011; **470**: 245–9.

16. Yin J, Rainka MP, Zhang X-X *et al.* A Highly Active Suzuki Catalyst for the Synthesis of Sterically Hindered Biaryls: Novel Ligand Coordination. *J Am Chem Soc* 2002; **124**: 1162–3.

17. Laha JK, Dhanalekshmi S, Taniguchi M *et al.* A Scalable synthesis of *meso*-substituted dipyrromethanes. *Org Process Res Dev* 2003; **7**: 799–812.

18. Lindsey JS, Schreiman IC, Hsu HC *et al.* Rothemund and Adler-Longo reactions revisited: Synthesis of tetraphenylporphyrins under equilibrium conditions. *J Org Chem* 1987; **52**: 827–36.

19. Tohara A, Sato M. An improved synthesis of *meso*-tetraanthrylporphyrin by a kinetically controlled Lindsey reaction. *J Porphyrins Phthalocyanines* 2007; **11**: 513–8.

20. De Oteyza DG, Frederiksen T. Carbon-based nanostructures as a versatile platform for tunable π-magnetism. *J Phys: Condens Matter* 2022; **34**: 443001.

21. Slayden SW, Liebman JF. The Energetics of aromatic hydrocarbons: An experimental thermochemical perspective. *Chem Rev* 2001; **101**: 1541–66.

22. Wu JI, Fernández I, Schleyer P v. R. Description of aromaticity in porphyrinoids. *J Am Chem Soc* 2013; **135**: 315–21.

23. Sun Q, Mateo LM, Robles R *et al.* Inducing open-shell character in porphyrins through surface-assisted phenalenyl π-extension. *J Am Chem S*oc 2020; **142**: 18109–17.

24. Mishra S, Yao X, Chen Q *et al.* Large magnetic exchange coupling in rhombus-shaped nanographenes with zigzag periphery. *Nat Chem* 2021; **13**: 581–6.

25. Jacob D, Fernández-Rossier J. Theory of intermolecular exchange in coupled spin-1/2 nanographenes. *Phys Rev B* 2022; **106**: 205405.

26. Lawrence J, Brandimarte P, Berdonces-Layunta A *et al.* Probing the magnetism of topological end states in 5-armchair graphene nanoribbons. *ACS Nano* 2020; **14**: 4499–508.

27. Mishra S, Beyer D, Berger R *et al.* Topological defect-induced magnetism in a nanographene. *J Am Chem Soc* 2020; **142**: 1147–52.

28. Zheng Y, Li C, Zhao Y *et al.* Engineering of magnetic coupling in nanographene. *Phys Rev Lett* 2020; **124**: 147206.

29. Wang S, Talirz L, Pignedoli CA *et al.* Giant edge state splitting at atomically precise graphene zigzag edges. *Nat Commun* 2016; **7**: 11507.

30. Karan S, Frank T, Preis T *et al.* Interplay of boundary states of graphene nanoribbons with a Kondo impurity. *Phys Rev B* 2022; **105**: 205410.

31. Kinikar A, Englmann TG, Di Giovannantonio M *et al.* Electronic decoupling and hole-doping of graphene nanoribbons on metal substrates by chloride intercalation. *ACS Nano* 2024; **18**: 16622–31.

32. Ou Z, Wang J, Zhang J *et al.* Uncovering the magnetic response of open-shell graphene nanostructures on metallic surfaces at different doping levels. *Sci China Phys Mech Astron* 2024; **67**: 226812.

33. Yamaguchi K, Fukui H, Fueno T. Molecular orbital (MO) theory for magnetically interacting organic compounds. Ab-initio MO calculations of the effective exchange integrals for cyclophane-type carbene dimers. *Chem Lett* 1986; **15**: 625–8.

34. Yamanaka S, Okumura M, Nakano M *et al.* EHF theory of chemical reactions Part 4. UNO CASSCF, UNO CASPT2 and R(U)HF coupled-cluster (CC) wavefunctions. *J Mol Struct* 1994; **310**: 205–18.

35. Ortiz JV. Dyson-orbital concepts for description of electrons in molecules. *J Chem Phys* 2020; **153**: 070902.

36. Kumar M, Soler-Polo D, Lozano M *et al.* Multireference theory of scanning tunneling spectroscopy beyond one-electron molecular orbitals: Can we image molecular orbitals? *J Am Chem Soc* 2025; **147**: 24993–5003.

37. Perdew JP, Burke K, Ernzerhof M. Generalized gradient approximation made simple. *Phys Rev Lett* 1996; **77**: 3865–8.

38. Krejčí O, Hapala P, Ondráček M *et al.* Principles and simulations of high-resolution STM imaging with a flexible tip apex. *Phys Rev B* 2017; **95**: 045407.

39. Calvo-Fernández A, Kumar M, Soler-Polo D *et al.* Theoretical model for multiorbital Kondo screening in strongly correlated molecules with several unpaired electrons. *Phys Rev B* 2024; **110**: 165113.

40. Saha S, Roy RK, Ayers PW. Are the Hirshfeld and Mulliken population analysis schemes consistent with chemical intuition? *Int J Quantum Chem* 2009; **109**: 1790–806.

41. Bader RFW, Bader RFW. Atoms in molecules: A quantum theory. Oxford, New York: Oxford University Press, 1994.
